# Experimental demonstration of corrugated nanolaminate films as reflective light sails

**DOI:** 10.1038/s41467-026-73414-4

**Published:** 2026-05-26

**Authors:** Matthew F. Campbell, Pawan Kumar, Jason Lynch, Ramon Gao, Adam Alfieri, John Brewer, Thomas J. Celenza, Mohsen Azadi, Michael D. Kelzenberg, Eric Stach, Aaswath P. Raman, Harry A. Atwater, Igor Bargatin, Deep Jariwala

**Affiliations:** 1 Department of Mechanical Engineering and Applied Mechanics, University of Pennsylvania, Philadelphia, PA USA; 2 Department of Electrical and Systems Engineering, University of Pennsylvania, Philadelphia, PA USA; 3 Thomas J. Watson Laboratory of Applied Physics, California Institute of Technology, Pasadena, CA USA; 4 Department of Materials Science and Engineering, University of California at Los Angeles, Los Angeles, CA USA; 5 Singh Center for Nanotechnology, University of Pennsylvania, Philadelphia, PA USA; 6 Department of Materials Science and Engineering, University of Pennsylvania, Philadelphia, PA USA

**Keywords:** Metamaterials, Metamaterials

## Abstract

Achieving laser-driven relativistic light sails would represent a tremendous breakthrough for humankind. Numerous sail designs have been proposed, but none satisfy all the stringent optical, mechanical, and mass constraints. Here we demonstrate a class of nanolaminate sails with strong and flexible hexagonally-corrugated microstructures. Our prototypes, fabricated from alumina and molybdenum disulfide using scalable semiconductor processing techniques, feature areal densities of  < 1 g ⋅ m^−2^, achieve experimentally-measured broadband reflectivities of  > 50%, and feature broadband absorptivities of  < 4% with a measurement uncertainty that overlaps with zero - indicative of our sail class’s potential for fast acceleration and ultra-low photon absorption. Moreover, we propose a sail’s maximum achievable relative velocity as a performance benchmark, and analyze optical, mechanical, and mass constraints for our design and others in the literature to highlight the strong potential of our class of sails. Our approach represents a promising step toward plausible relativistic interstellar propulsion.

## Abstract (web summary)

Laser-driven light sails capable of reaching relativistic velocities in space have been proposed a century ago, but their experimental realization remains challenging. Here, the authors report the fabrication of flexible corrugated nanolaminate sails based on alumina and molybdenum disulfide, showing promising mass, optical and mechanical characteristics.

## Introduction

**Fig. 1:**
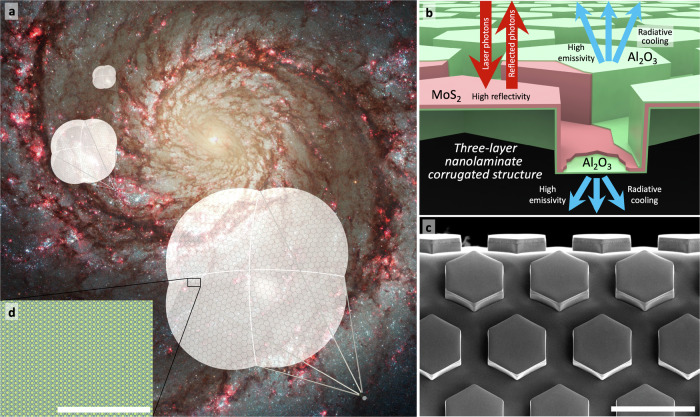
Overview of laser-accelerated light sails. **a** Light sails carry gram-scale payloads at relativistic velocities for interstellar travel. **b** Computer cut-away rendering of sail film showing three-layer nanolaminate corrugated structure. The MoS_2_ provides high reflectivity for acceleration and the flanking Al_2_O_3_ layers yield high emissivity for radiative cooling. The hexagonally corrugated microstructure increases the sail’s bending rigidity, preventing wrinkles and allowing it to maintain its shape as it accelerates. **c** Scanning electron micrograph (SEM) showing prototype film bent away from the camera to emphasize its three-dimensional structure. **d** Micrograph of fully suspended prototype film (Si substrate removed). Scale bars: (**c**) 50 μm, (**d**) 1 mm. Galaxy image obtained from NASA^136^.

One hundred years ago, Friedrich Zander and Konstantin Tsiolkovsky proposed reflecting photons by thin mirror-like sheets as a means of space propulsion^1,2^. Since that time, significant effort has been devoted to materializing their vision in the form of laser-driven light sails: highly reflective, ultra-lightweight membranes that accelerate miniature chip satellites to relativistic velocities^3–15^ (Fig. 1). This design challenge is formidable. Light sails must be highly reflective at both the laser wavelength and at slightly longer wavelengths due to the Doppler shift as the sail’s velocity increases^16^. They must also exhibit essentially zero absorptivity (*α* ≲ 10^−9^) in the Doppler-shifted laser wavelength range and emit efficiently at longer infrared wavelengths (*ε*_*e*_ ≳ 10^−3^) to avoid overheating^17^. Furthermore, light sails must be lightweight yet mechanically robust, with ultra-low areal densities (*ρ*_*a*,*target*_ ≈ 0.1 g ⋅ m^−2^)^6^ and high bending stiffnesses to avoid wrinkles^18^. Many previous designs, though maximizing reflectivity or minimizing areal density, struggled to manage the tradeoffs between all of these requirements simultaneously^11,16–32^.

Therefore, by considering optical/thermal, mechanical, and mass budget constraints concurrently, we have developed and tested corrugated nanolaminate sails. Our films consist of a high-refractive-index multilayer molybdenum disulfide (MoS_2_) core flanked by emissive alumina (Al_2_O_3_) face sheets for thermal management. Below, we show that this low-areal-density combination provides high reflectivity and low absorptivity near the proposed laser wavelength of *λ*_*l*_ = 1.2 μm. Moreover, our sails feature a hexagonally-patterned out-of-plane architected structure, which enhances their bending stiffness to prevent them from wrinkling.

## Results

### Film fabrication

MoS_2_ is an advantageous material for laser-driven light sails because it has a high real index of refraction *n* near *λ*_*l*_ = 1.2 μm and can be fabricated in atomically-perfect sheets that exhibit near-zero infrared absorptivity^6,33^. In order for the high *n*-values to beget high reflectivity, however, the MoS_2_ films must be grown as atomically-smooth sheets with multi-nanometer-scale thicknesses. Achieving such thick smooth films has historically been challenging^34^. We accomplished this using a two-step fabrication approach, in which we first sputtered Mo onto a substrate, being careful to maintain precise control of the grain size and roughness, and subsequently sulfurized it into high-quality MoS_2_ multilayers in an elevated temperature H_2_/H_2_S environment^35^ (see Methods).

**Fig. 2:**
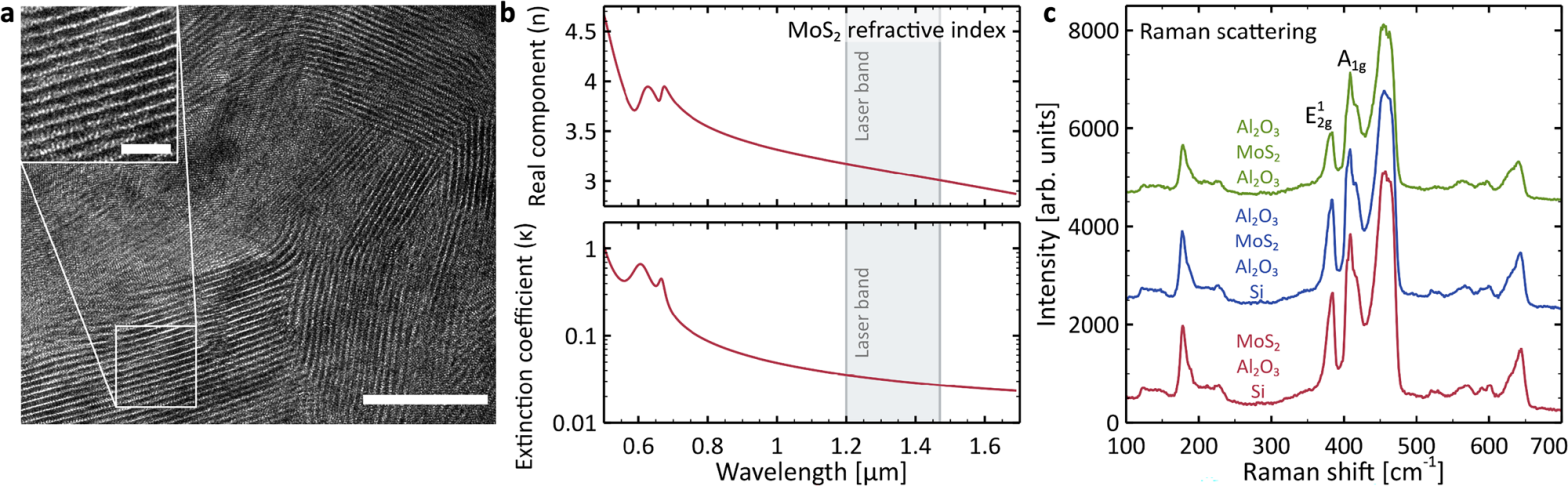
Characterization of thick MoS_2_ films. **a** Transmission electron micrograph (TEM) of a roughly 75-nm-thick sample. **b** Refractive index information ${\mathfrak{n}}=n+{{\rm{i}}}\kappa$ for a MoS_2_ film grown on Al_2_O_3_, obtained *via* spectroscopic ellipsometry. Here and elsewhere, the shaded gray range denotes the laser band, or the Doppler-shifted wavelength range corresponding to a final relative velocity of ${\beta }_{f}=\frac{{v}_{f}}{c}=0.2$ (*v*_*f*_ is the sail velocity and *c* is the speed of light; *λ* = 〈1.2, 1.4697〉 μm). The relative uncertainties of the *n* and *κ* data within the laser band are ± 0.8% and ± 2.6%, respectively (based on a 90% confidence interval for the fit). **c** Raman scattering measurements of a MoS_2_ film on an Al_2_O_3_-coated Si substrate: uncovered (bottom record), with an Al_2_O_3_ top coating (middle record), and with the Si substrate removed (top record). Scale bars: (**a**) 10 nm, inset of (**a**): 2 nm.

Using this approach, we produced thick MoS_2_ films on silicon, silicon dioxide, and alumina substrates and interrogated them to verify their quality (see Methods). Figure 2(a) presents a planar-view transmission electron micrograph (TEM) of a MoS_2_ film transferred from a SiO_2_/Si substrate to a Cu-TEM grid that clearly shows individual layers, indicating that the film has a vertically-oriented multilayer van der Waals structure (see also Supplementary Fig. 7 for cross-sectional TEM images). We augmented this data by conducting atomic force microscopy (AFM) scans of samples on SiO_2_/Si substrates (see Supplementary Fig. 8); our measured root-mean-square (RMS) roughness values of ~ 5 nm over 100 μm^2^ areas indicate that our films are morphologically smooth. We also performed a spectroscopic ellipsometry (SE) analysis on a 60-nm-thick sample (Fig. 2(b)), finding that a graph of the complex index of refraction ${\mathfrak{n}}=n+{{\rm{i}}}\kappa$ versus wavelength shows the same trends as measurements of bulk and monolayer samples in the literature^33,36–38^ (see Supplementary Fig. 17). Our measured extinction coefficient of *κ* < 0.1 is consistent with complete transformation of the Mo into MoS_2_, and also indicates the potential of our films to achieve low optical absorptivity if fabricated to ultra-high purity standards. Furthermore, our Raman scattering measurements (Fig. 2(c)) confirm the formation of a crystalline multilayered MoS_2_ structure while negating any remaining oxide-based compounds.

**Fig. 3:**
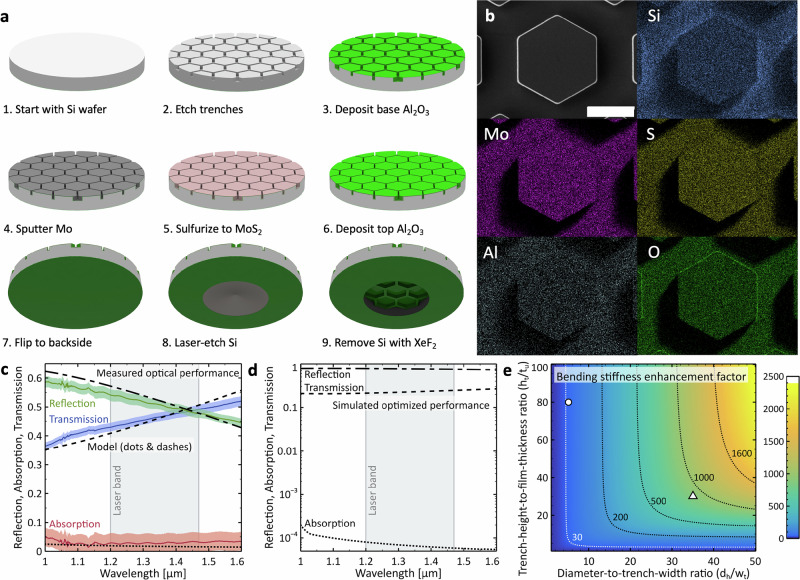
Fabrication and characterization of corrugated Al_2_O_3_-MoS_2_-Al_2_O_3_ films. **a** Fabrication steps for sail film prototypes in the indented trench configuration (see Methods; for information on scaling this process to larger suspended sizes, see Supplementary Note 21). **b** Scanning electron micrograph (SEM) and energy dispersive X-ray spectroscopy (EDS) images of multilayer film on Si substrate. The presence of color in the images corresponds to the presence of the indicated element. See Supplementary Fig. 9 for the associated spectrum. **c** Experimentally-measured normal-incidence reflectivity (*ϱ*_*λ*,⊥_) and transmissivity (*τ*_*λ*,⊥_) spectra for fabricated prototype film, along with resulting absorptivity (*α*_*λ*,⊥_ = 1 − *ϱ*_*λ*,⊥_ − *τ*_*λ*,⊥_) spectrum. Shaded areas for *ϱ*_*λ*,⊥_ and *τ*_*λ*,⊥_ reflect a 3% uncertainty along with the maximum variation among three sets of measurements (see Supplementary Note 9 for a detailed description). Black dash/dot lines show simulated spectra obtained using the transfer-matrix method with complex indices of refraction obtained from ellipsometry measurements (see Methods and Supplementary Note 6). **d** Simulated spectrum for proposed optimized film, calculated using indices of refraction obtained from the literature^38,41^. Shaded gray regions in (**c**) and (**d**) denote the laser band. **e** Calculated bending stiffness enhancement factor of hexagonally corrugated structures relative to their planar (non-corrugated) counterparts^47^ as a function of the ratio of the hexagon diameter to trench width ($\frac{{d}_{h}}{{w}_{t}}$, abscissa) and the ratio of the trench height to the total composite film thickness ($\frac{{h}_{t}}{{t}_{u}}$, ordinate). Circle marks fabricated prototype film and triangle marks feasible proposed optimized film (see Supplementary Notes 3 and 13). Prototype film dimensions: *d*_*h*_ ≈ 77 μm (measured flat-to-flat), *w*_*t*_ ≈ 15 μm, *h*_*t*_ ≈ 10 μm, *t*_*A*,*b*_ ≈ 21 nm (bottom Al_2_O_3_ thickness), *t*_*M*_ ≈ 53 nm (MoS_2_), *t*_*A*,*t*_ ≈ 51 nm (top Al_2_O_3_), *ρ*_*a*,*c*_ ≈ 0.7 g ⋅ m^−2^ (corrugated film areal density). Proposed optimized film dimensions: *d*_*h*_ = 70 μm, *w*_*t*_ = 2 μm, *h*_*t*_ = 3 μm, *t*_*A*,*b*_ = *t*_*A*,*t*_ ≈ 19 nm, *t*_*M*_ ≈ 63 nm, *ρ*_*a*,*c*_ ≈ 0.5 g ⋅ m^−2^. We define the total/unified film thickness to be *t*_*u*_ = *t*_*A*,*b*_ + *t*_*M*_ + *t*_*A*,*t*_. Scale bar: (**b**) 20 μm.

Our thick MoS_2_ films provide favorable optical constants for reflection but must be augmented to achieve sufficient thermal management and mechanical robustness. For thermal management, we sandwiched our multilayer MoS_2_ films between thin alumina layers, creating the film’s characteristic nanolaminate structure (see Fig. 3(a)). The Al_2_O_3_ is minimally absorbing within the laser wavelength range but emits strongly at longer infrared wavelengths^39–41^, allowing the sail to reradiate any incident laser photon energy that it absorbs^18^. Mechanically, Al_2_O_3_ and MoS_2_ are a natural pair; they have well-matched thermal expansion coefficients^42,43^ and can be readily fabricated one upon the other^44,45^ (see Supplementary Notes 1 and 17).

For mechanical robustness, we imparted an out-of-plane corrugated microstructure pattern to our films by sequentially depositing the film layers on Si substrate molds consisting of hexagons separated by either trenches cut into the substrate or ribs protruding from it (see Methods). The out-of-plane “U”-shapes of this pattern greatly increase the bending stiffness of the architected films relative to their non-corrugated counterparts (the bending stiffness of a beam scales with the cube of its effective thickness^46^), much like the vertical section of an “I”-beam increases its rigidity by increasing its bending moment of inertia^47^ (see Fig. 3(e) and Supplementary Note 3).

After forming the corrugated molds and depositing the Al_2_O_3_ and MoS_2_ films thereon, we used a two-step process to suspend the sail prototypes for testing. First, we used laser ablation to remove a majority of the backside of the corrugated Si mold. Second, we gently removed the remaining Si using a gaseous XeF_2_-etching process (see Methods). We fabricated circular suspended prototype films with diameters up to roughly 3 mm using this process, sufficient for the optical testing of this work. To increase this to wafer-scale sizes, front-side substrate etching processes could be performed by cutting small holes in the film^31,48,49^, or roll-to-roll nanoimprint lithography could be used^50,51^ (see Supplementary Note 21).

### Film characterization

Having fabricated both chip-bound and freestanding film prototypes, we interrogated them to understand their composition and optical qualities. Figure 2(c) provides a comparison of the Raman scattering signals for a MoS_2_ film on an Al_2_O_3_-coated Si substrate in three configurations: (1, bottom record) uncovered, (2, middle record) with an Al_2_O_3_ top coating, and (3, top record) with the Si substrate etched away. The prominent and consistent ${\,{{\rm{E}}}}_{{{\rm{2g}}}}^{1}$ and A_1g_ peaks indicate good crystallinity of the MoS_2_ throughout our fabrication process, and the difference in their positions (roughly 25 cm^−1^) is consistent at each step, which signals good MoS_2_ uniformity^36^. The absence of the characteristic peak near 520 cm^−1^ in the top record reflects the fact that the Si mold was completely etched away. Figure 3(b) shows energy dispersive X-ray spectroscopy (EDS) images of a single hexagonal unit cell still attached to its Si substrate, indicating the presence of all relevant elements.

We conducted optical normal-incidence reflectivity (*ϱ*_*λ*,⊥_) and transmissivity (*τ*_*λ*,⊥_) measurements of a suspended prototype film at wavelengths surrounding *λ*_*l*_ = 1.2 μm, shown in Fig. 3(c) (see Methods and Supplementary Note 9). The data, shown with roughly 3% uncertainty, are in good agreement with our thin film optical transfer-matrix method simulations (based on optical constants we measured for MoS_2_ and Al_2_O_3_ using spectroscopic ellipsometry; see Methods) and indicate good broadband reflectivity (*ϱ*_*λ*,⊥_ > 0.5 within our proposed laser band, *λ* = 〈1.2, 1.4697〉 μm). Moreover, the broadband absorptivity of *α*_*λ*,⊥_ = 1 − *ϱ*_*λ*,⊥_ − *τ*_*λ*,⊥_ < 0.04 has an associated uncertainty that overlaps with zero, suggesting the capacity of our class of films to achieve ultra-low photon absorption if optimized and fabricated with fewer imperfections. To our knowledge, our measurements are the lowest experimental broadband absorptivity data for a prototype light sail film. (Note that recently, silicon nitride measurements with lower absorptivities have been reported at single wavelengths for suspended light sail prototypes^13,52^ and over a broader wavelength range for other applications^53^.) Although our transfer-matrix method simulations suggest an absorptivity greater than zero, we emphasize that our experimental results highlight the strong potential of our nanolaminate design.

In addition to being reflective and low-absorbing, our sails have low areal densities. We obtained these values using the measured corrugation pattern dimensions and film thicknesses, together with published material density values^54–56^. Our corrugated films and their planar counterparts have areal densities of *ρ*_*a*,*c*_ ≈ 0.7 g ⋅ m^−2^ and *ρ*_*a*,*p*_ ≈ 0.5 g ⋅ m^−2^, respectively, both of which fall within an order of magnitude of the target set by the Breakthrough Starshot Foundation^6,7,10,12^.

Although corrugated films weigh more than planar films, their structure brings critically important mechanical properties. First, corrugation reduces the films’ contact areas and increases their bending stiffnesses (see Fig. 3(e)), such that they are less sticky, wrinkle less, and can be more readily assembled into working structures^47,57^. Second, the corrugation can be tailored to increase the films’ “stretchiness” to prevent tears at points of concentrated stress, such as those associated with sail support frames or cable tethers^58^ (see Supplementary Figs. 14 and 15). Third, as demonstrated by Davami et al.^47^, the corrugated structure prevents crack propagation by deflecting cracks at the vertical trench or rib walls, which is important given the likelihood of punctures by space dust^59–62^. Fourth, our films’ corrugation allows them to undergo and recover from extreme bending, which could allow fleets of light sails to be tightly folded, carried into low Earth orbit, and deployed^57^. Finally, corrugated microstructures are not limited to Al_2_O_3_ and MoS_2_; other high-performance light sail materials, including Si^22,28,32^, SiO_2_^16^, Si_3_N_4_^17,26,30,31^, and TiO_2_^27^ could also be fabricated in corrugated shapes, providing these benefits across a wide range of sail designs.

A key figure of merit for light sails is their acceleration length *L*, that is, the distance they travel while being illuminated by laser-generated photons until reaching their target relative velocity, typically ${\beta }_{f}=\frac{{v}_{f}}{c}=0.2$, where *v*_*f*_ is the final sail velocity and *c* is the speed of light. This length scales as $L \sim \frac{{m}_{tot}c{v}_{f}^{2}}{2{\Phi }_{l}{\varrho }_{a}}$, where *m*_*tot*_ is the total mass of the sail and payload, *Φ*_*l*_ is the laser output power, and *ϱ*_*a*_ is the sail’s average reflectivity near the laser wavelength^18^. The acceleration length is of practical engineering importance because both the time duration that the laser array must produce photons and the laser array diameter on Earth required to perfectly focus the beam on the sail scale with it (see Supplementary Note 5). We determined the acceleration trajectory for a sailcraft consisting of a *m*_*s*_ = 1 g flat circular sail composed of the film we fabricated (using the experimentally-measured reflectivity of Fig. 3(c)) towing a *m*_*p*_ = 1 g payload (tethers plus probe chip) while illuminated by a constant *Φ*_*l*_ = 100 GW laser array with an output wavelength of *λ*_*l*_ = 1.2 μm. Our calculations predict an acceleration length to *β* = 0.2 of *L* ≈ 15 Gm, a laser-on time of 7 min, and a required laser array diameter of 26 km. These values indicate the practical feasibility of our films as light sails^17,18,21,22^.

## Discussion

**Fig. 4:**
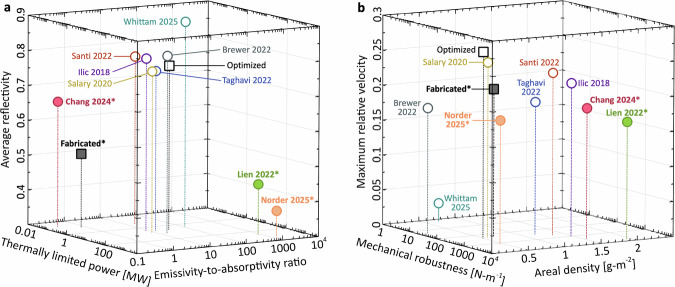
Optical, mechanical, and performance comparison of several light sail films. Sails found in the literature are shown with circles, and our fabricated prototype and proposed optimized films are shown with squares. Solid shapes labeled with bold text and an asterisk symbol (*) denote films that have at least some experimental measurements^26,30,31^ and open shapes denote theoretical calculations^16,17,22,27,28,32^. **a** Comparison of laser-band average reflectivity $\overline{{\varrho }_{\perp }}$, ratio of infrared effective emissivity (at *T* = 1000 K, *λ* = 2 − 14 μm) to laser-band average absorptivity $\frac{{\varepsilon }_{e}}{\overline{{\alpha }_{\perp }}}$, and thermally limited power *Φ*_*s*,*max*_. **b** Comparison of maximum relative velocity *β*_*max*_, areal density *ρ*_*a*_, and membrane mechanical robustness *℘*. The membrane mechanical robustness *℘* quantifies a sail’s ability to resist photon pressure forces, and the maximum relative velocity *β*_*max*_ estimates the fastest speed the sail can attain when constrained by mechanical forces and optical diffraction (see Methods, Supplementary Note 14, and Supplementary Table 2).

We have experimentally demonstrated and optically tested a light sail prototype film that comes within an order of magnitude of the required areal density and exhibits broadband reflectivity > 50%, while simultaneously holding promise for ultra-low absorptivity with continued improvement in fabrication techniques. Figure 4(a) compares our film to other sail prototypes in the literature in terms of their average reflectivity in the Doppler-shifted laser wavelength range (the laser band), their infrared emissivity-to-laser band average absorptivity ratio for thermal management, and the maximum laser power they can tolerate without overheating (see Methods)^16,17,22,26–28,30,31^. Likewise, Fig. 4(b) examines the sails’ acceleration performance, mechanical strength, and areal density values (see also Supplementary Table 2). Our measure of a sail’s mechanical strength is a quantity that we call the membrane mechanical robustness, defined here as the product of a film’s thickness and tensile yield strength; it is indicative of a sail’s resistance to failure/collapse upon incident laser photon pressure. Similarly, our measure of accelerative performance is the maximum relative velocity *β*_*max*_ to which a sail can be accelerated without tearing due to the photon pressure, under the assumption of zero optical absorptivity (i.e., no thermal limit, see Methods). Taken together, Figures 4(a, b) illustrate the significant potential of our class of sail films for use in laser-propelled relativistic interstellar travel. Importantly, our model indicates that our prototype is the only one that could propel a sailcraft to the *β* = 0.2 Starshot goal^6,7,10,12^ based on an experimentally-measured, rather than a theoretically calculated, reflectivity spectrum.

Our prototype sail is one embodiment among a large class of similarly-structured corrugated nanolaminate films. To demonstrate this design class’s potential, under the assumption that future fabrication advances will allow thick, optically perfect, and mechanically strong MoS_2_ layers to be formed, we systematically changed the Al_2_O_3_ and MoS_2_ film thicknesses in order to maximize *β*_*max*_ within the design space (see Methods and Supplementary Note 13). Our models predict that the optimal film, whose simulated optical performance is shown in Fig. 3(d), can achieve *β*_*max*_ ≈ 0.26 (i.e., travel more than 25% faster than the Starshot goal of *β* = 0.2). It accomplishes this by decreasing the Al_2_O_3_ layer thicknesses (~ 19 nm each) to lower the areal density of the corrugated film (~ 0.5 g ⋅ m^−2^) while increasing the MoS_2_ thickness (~ 63 nm) to increase the reflectivity (laser band average: ~ 0.77), all while maintaining the minimum required mechanical robustness to prevent tearing at the maximum laser power. Even higher ultimate speeds could be achieved by throttling the laser power as the sail accelerates^7,18^. Our optimized prototype design achieves a higher *β*_*max*_ value than all other designs in Fig. 4. Though the film proposed by Salary and Mosallaei^22^ comes close, our optimized proposed prototype represents an experimentally-proven class of sails, suggesting the practical viability of our designs.

Although our class of corrugated nanolaminate light sails represents great strides in reflectivity, mass, and mechanical integrity, our films still need to be significantly improved in terms of their absorptivity and emissivity values. In fact, we emphasize that none of the sails represented in Fig. 4 have a sufficiently high ratio of emissivity to absorptivity $(\frac{{\varepsilon }_{e}}{\alpha } \sim 1{0}^{6})$ to survive the strong laser photon fluxes required to achieve relativistic velocities within reasonable acceleration lengths (see Supplementary Notes 10 and 14). This can be seen in the units of the thermally limited power axis of Fig. 4(a), which are in megawatts rather than gigawatts. For this reason, we did not consider thermal effects in our maximum relative velocity calculations (Fig. 4(b)).

We envision multiple routes toward realizing higher quality films with superior thermal performance. To achieve lower laser band absorptivity values, the atomic layer deposited amorphous Al_2_O_3_ could be annealed into crystalline sapphire^63,64^, or the MoS_2_ could be deposited in new ways^65,66^, including atomic layer deposition^67^ or sputtering^68^. Several studies indicate that high-quality Al_2_O_3_ and MoS_2_ films can have very low extinction coefficients^33,39,69,70^. Methods to increase the films’ emissivity could include patterning micron-scale features (larger than the longest wavelength in the laser band) or incorporating different face sheet materials such as SiO_2_^71^, Si_3_N_4_^17,41^, or (Al:)ZnO^72^. More information can be found in Supplementary Note 16.

With regard to our proposed optimized sail design, its absorptivity in the laser band will scale with the extinction coefficients of Al_2_O_3_ and MoS_2_. To explore the possibility that future fabrication advances will allow minimally-absorbing films to be fabricated, we conducted a time-dependent energy balance to estimate the sail temperature during the acceleration phase (up to *β*_*f*_ = 0.2) of our proposed optimized design using published index of refraction values for crystalline alumina^39^ and an extinction coefficient of *κ* = 10^−8^ for MoS_2_, which approaches that measured for monolayers^33^ (see Supplementary Note 15). Even with a laser output power of 100 GW, our simulation indicated that, given these extinction coefficients, the sail temperature would not exceed the MoS_2_ high-vacuum sublimation point of about 1000 K^73^. This suggests that continuing improvements to film microfabrication processes will make our sail design viable for relativistic space flight.

Related questions remain. Further research is needed to quantify the impact of temperature on thin-film absorption coefficients to predict thermal runaway scenarios^62,74^ and to explore the importance of two-photon absorption events for high photon fluxes^75,76^. In addition, more work is needed to address concerns of beam-riding stability, which could be related to both the film’s nano-architecture and its macroscopic shape^11,19,22,23,28,77–84^. These topics are beyond the scope of this work, because our purpose is to investigate the fabrication and optical properties of nanolaminate Al_2_O_3_-MoS_2_ films (but see Supplementary Notes 17 and 19).

Our corrugated nanolaminate films have significant potential beyond relativistic interstellar travel. A more immediate application is intra-solar system transport for wafer-scale spacecraft^29,83^. For instance, thermal constraints aside, a 1 GW laser array with a diameter of 10 km could accelerate a 10-g sailcraft employing a flat circular sail with our fabricated prototype’s reflectivity (Fig. 3(c)), areal density, and mechanical robustness to 1% of the speed of light (*β* = 0.01) in under 3 h, allowing it to reach Jupiter from Earth in less than 4 days. Such rapid transit would fundamentally alter humankind’s approach to exploring our neighboring planets and sun, allowing us to take measurements with cutting-edge instruments and rapidly respond to phenomena such as sunspots, storms, and geological activity^85,86^. Similarly, fleets of sailcraft could be sent to pass through the focal point of the solar gravitational lens, located roughly 542 AU from the Sun, to sequentially image a distant planet with km-scale resolution^30,87–89^.

We envision other uses for our design, as well. Our films could be employed as ultralightweight optical elements for orbiting laser-based deep space optical communication (DSOC) equipment^90^, offering high-bandwidth data transmission without the atmospheric interference and shadowing of ground-based telescopes. Similarly, arrays of our films could be placed at the inner Lagrangian point (L1) to deflect sunlight and cool the Earth, in the event of excessive warming and climate change^91^. Finally, the relatively high emissivity of our designs could make them useful for passive radiative cooling of Earth-or-space-based equipment^92^.

In summary, we created a class of light sail films that makes interstellar travel plausible at relativistic speeds. These sails feature a nanolaminate structure consisting of high-quality multilayer MoS_2_ films flanked by thin Al_2_O_3_ face sheets. This combination provides high laser-band reflectivity (> 50%) and low absorptivity at a laser output wavelength of 1.2 μm, while offering strong emission at longer infrared wavelengths for thermal management. Our films also feature microscale out-of-plane corrugation, which makes them less prone to wrinkling. Importantly, these films’ areal densities are within an order of magnitude of the target areal density of 0.1 g ⋅ m^−2^, positioning them for practical relativistic spaceflight. Moreover, we anticipate that future fabrication advances will lead to completely in-plane-aligned, near-epitaxial quality multilayer MoS_2_ films, which will increase the reflectivity of these sails and reduce their mass. Our reflective, strong, and lightweight nanolaminate corrugated films represent a promising approach towards the realization of Zander and Tsiolkovsky’s vision of photon-driven relativistic interstellar space travel.

## Methods

### Fabrication

#### MoS_2_ films

We fabricated our MoS_2_ films *via* a two-step process, in which we first deposited molybdenum onto a substrate and subsequently sulfurized it into high-quality MoS_2_. We slowly deposited the Mo onto silicon, silicon dioxide, or sapphire substrates using direct current sputtering (Denton Explorer 14, 25 W, 3 mTorr pressure, rate  ≈ 1  ${{\rm{nm}}}\cdot {\min }^{-1}$) in film thicknesses of roughly 15 nm. Importantly, our low deposition pressure helped to ensure highly directional sputtering, allowing uniform and conformal coverage on the vertical corrugated surfaces of our molds (trench or rib walls; see Fig. 3)^93^. We used atomic force microscopy (see below) to measure the thicknesses of the deposited films by examining the step height created *via* shadowing by the polyimide tape used to secure the substrates in the sputtering tool. For the subsequent sulfurization process, we used a horizontal tube furnace chemical vapor deposition system (Structured Materials Industries, Inc.). After placing the samples in the furnace at room temperature, we heated the tube to 750^∘^C in the span of 15 min in a H_2_/Ar environment (volumetric flow rates: 10 sccm/100 sccm, total pressure: 10 Torr). Upon the tube reaching 750^∘^C, we introduced H_2_S gas (volumetric flow rate: 25 sccm) while maintaining the same H_2_ and Ar flow rates and the same total pressure of 10 Torr. We maintained the temperature, pressure, and gas flow rate for 6 h, at which point we cooled the samples by turning off the heater power and stopping the flows of H_2_ and H_2_S (Ar continued to flow at 100 sccm at a total pressure of 10 Torr during the cooling step.) The resulting films had thicknesses of roughly 60–70 nm, as measured and confirmed *via* atomic force microscopy and ellipsometry.

#### Corrugated nanolaminate films

Our prototype fabrication process can be divided into three parts: (1) etching the shape of the corrugated Si mold; (2) depositing the films to form the nanolaminate stack; and (3) removing the Si mold to create a free-standing film. These steps are summarized in Fig. 3(a) and in Supplementary Fig. 1.

We formed the corrugated molds out of 200-μm-thick double-side-polished Si wafers (p(B)-doped, resistivity 1–10 Ω, University Wafer). We spin-coated an adhesion layer of SurPass 3000 (3000 ${{\rm{rev}}}\cdot {\min }^{-1}$ for 1 min) followed by a 2.5 μm layer of S1818 photoresist (3000 ${{\rm{rev}}}\cdot {\min }^{-1}$ for 1 min), and performed a soft bake of the wafer on a hot plate (115^∘^C for 1 min). We then exposed a hexagonal pattern (70 mJ ⋅ cm^−2^, vacuum contact, SUSS MicroTec MA6 Gen3 Mask Aligner), developed the photoresist (AZ 300 MIF for 1.5 min, with a brief deionized H_2_O rinse and N_2_ dry), and baked the wafer on a hot plate again (115^∘^C for 10 min). We used both darkfield and brightfield photomasks, which, combined with our positive-tone photoresist, allowed us to produce Si molds with indented trenches and protruding ribs, respectively. The hexagon diameter *d*_*h*_ (flat-to-flat) and trench/rib width (*w*_*t*_ or *w*_*r*_) are important design parameters. For the purposes of this study, to avoid tears and folds that are possible when handling in air, we selected low diameter-to-trench/rib-width ratios. There is a practical lower limit to this ratio, however; designs should at least select *d*_*h*_≥3*w*_*t*_ (the same holds for *w*_*r*_) to prevent film creases at vertical hexagon walls^57^. An example of this can be seen in Fig. 1(c), where we selected *d*_*h*_ ≈ 36 μm and *w*_*t*_ ≈ 15 μm to allow the film to bend and show its three-dimensional architecture. For future designs to be handled in vacuum environments, significant improvements in stiffness could be realized by using thinner trenches/ribs (lower *w*_*t*_ or *w*_*r*_) or larger diameter hexagons (higher *d*_*h*_); see Fig. 3(e).

Continuing in our fabrication process, we used deep reactive ion etching (DRIE) to remove the exposed Si areas (hexagon edges to form indented trenches or hexagon areas to form protruding ribs) to a depth of roughly 10 μm (as verified using stylus profilometry with a KLA Tencor P7 2D machine). We then stripped the photoresist using sonication (70^∘^C in KL Remover 1000 for 10 min) and O_2_ plasma ashing (1 h, 300 W, 100 sccm O_2_, Anatech SCE-108 Barrel Asher). In some cases, we found that, due to their high aspect ratios, some of the protruding ribs cracked during the sonication process. A simple workaround was to replace the sonication step with a longer quiescent soak (an alternative could also be to forgo the liquid-cleaning step altogether^94^). The wafers designed with trenches were not subject to cracking, however. Finally, we cleaved the wafer into 1.5 cm-side-length squares.

We deposited the film layers sequentially as follows. First, we used atomic layer deposition (ALD) at 250^∘^C to coat the chips with alumina (Al_2_O_3_), using precursors water and trimethylaluminium (Cambridge Nanotech Savannah S-200 reactor). ALD is a conformal technique, such that the vertical sides of the trenches/ribs of the Si mold, as well as the horizontal areas, were coated. Importantly, we positioned the chips on standoffs in the chamber, such that both their front and back sides were coated, completely encapsulating the Si. This alumina coating typically had a thickness of 20–50 nm, as controlled by the number of deposition cycles and measured using a spectroscopic ellipsometer (either Woollam V-VASE or Filmetrics F40) on a Si test chip placed in the chamber during the deposition. Next, we formed the MoS_2_ layer using the process outlined above, which involved first conformally sputtering a Mo film (Denton Explorer14 Magnetron Sputterer) and subsequently sulfurizing it in a high-temperature tube furnace. Thirdly, we used ALD a second time to conformally coat the front and backsides of the chips with Al_2_O_3_, again at 250^∘^C using the precursors and reactor listed above.

Lastly, we removed the Si substrate in the area of interest, usually a 0.5–3 mm diameter circle in the center of the chip, to reveal a fully-suspended corrugated nanolaminate film. This process involved two steps. First, we rastered a 532-nm laser (IPG IX-280-DXF) over the backside of the chips to ablate away the Al_2_O_3_ coating and a majority of the Si substrate. We carefully controlled the power and cycle time such that the etch depth was deepest (more material removed) in the center of the area and shallower at the perimeter. This promoted etching of the area from the center towards the perimeter in the subsequent XeF_2_ step, which protected the suspended film against tears. We also used a N_2_ purge to partially suppress the thermal formation of SiO_2_ dust in the ablation region (note that backside DRIE could potentially be used instead of laser ablation^95^). Next, we used gaseous XeF_2_ etching to remove the remaining backside Si in the area of interest (SPTS Xactix). We performed etching in cycles to prevent overheating, where each cycle consisted of a 75 s long etch in 3 Torr undiluted XeF_2_ followed by a 3 s vacuum rest. Importantly, we designed and 3D-printed a miniature fixture (FormLabs Form3 printer, standard resin, highest resolution) to fully enclose each chip as it was etched (see Supplementary Fig. 6). The fixture held the chips in a vertical orientation and contained small (750 μm diameter) holes to expose both sides of the chip to gas at equal pressures, which we found to be important in preventing the thin released films from tearing when venting the chamber back to atmospheric pressure after the etching process was complete. XeF_2_ etching is highly selective to Si over Al_2_O_3_, allowing us to easily remove the Si substrate in the area where the alumina had been laser-etched away. Some lateral Si etching also occurred during the XeF_2_ process, which slightly increased the area of the suspended film over that opened in the backside by the laser-ablation step. We also remark that the pinhole-free nature of ALD is important in preserving the MoS_2_ during the XeF_2_ process (XeF_2_ etches MoS_2_), because the MoS_2_ was completely and conformally encapsulated by the Al_2_O_3_.

Finally, we note that the above process enabled us to produce corrugated films suspended on Si substrate frames, which were ideal for optical characterization purposes. However, the circular diameter of the largest suspended film we created was approximately 3 mm (the same size as that created by Chang et al.^30^); this method would be sub-optimal for producing the large-area films required to construct a light sail. We suggest that one route to producing larger films would be to cut holes in the film and use vapor XeF_2_ etching to remove the substrate Si from the front (this strategy, with SF_6_ etching, was used by Norder et al.^31^). We have explored this successfully using alumina-only films^48,49^. Another route could be roll-to-roll nanoimprint lithography processing techniques conducted directly in the vacuum of low-Earth orbit^50,51,96,97^. More details can be found in Supplementary Note 21.

### Characterization

#### Atomic force microscopy

We measured the thicknesses of our Mo and MoS_2_ films and characterized the roughness of our MoS_2_ films using an atomic force microscope in tapping mode (AIST-NT Co.). We processed the images using Gwyddion software^98^, using the plane level and level rows functions to remove any non-flat background.

#### Spectroscopic ellipsometry

We performed spectroscopic ellipsometry measurements using a J. A. Woollam W-VASE ellipsometer at wavelengths of roughly 0.4–1.7 μm. We used the CompleteEase software package to fit the data using a multi-Lorentz oscillator method to minimize the root mean square error^99^.

#### Raman scattering

We used a confocal micro-Raman spectroscopy system (LabRAM HR Evolution, HORIBA Instruments, Inc.) to measure all the Raman scattering characteristics of our multi-layered MoS_2_ films. We used a 633 nm laser to excite the sample while keeping a 1800 lines ⋅ mm^−1^ grating to obtain high spectral width resolution. We used a 3.2% neutral density filter to keep the laser excitation power within a safe range to prevent local heating/modulation during the sample measurement. We used the same measurement conditions for Raman imaging, achieving a very high spatial resolution through the use of 200 nm ⋅ px^−1^ steps (governed by a Märzhäuser Wetzlar X-Y stage).

#### Transmission electron micrography

We used a JEOL F200 system to acquire high-resolution transmission electron micrographs (TEM). The instrument was equipped with a 200 kV cold-field emission electron source and two detectors (Gatan, Inc.), and we used Gatan DigitalMicrograph 3.5 software to acquire images. We prepared our samples according to two different methods. For planar views, we used the wet-chemical transfer ("scooping”) technique^100^ to port films grown on Si/SiO_2_ substrates directly to TEM grids. For cross-sectional images, we prepared samples using the Xe plasma focused ion beam approach^101^ and subsequently transferred them to half-grids using the in situ liftoff technique^102^.

#### Scanning electron micrography and energy dispersive X-ray spectroscopy

We acquired our scanning electron micrographs (SEM) and energy dispersive X-ray images using a TESCAN S8000X machine. In SEM mode we used an electron beam energy of 5 keV and an in-lens detector to capture high-resolution pictures.

#### Laser reflection and transmission

Briefly, we focused tunable, chopped, monochromatic, linearly polarized laser light onto our fully suspended nanolaminate film samples (< 10-μm-diameter spot size, centered within a single hexagon unit cell) using a near-infrared objective (20×, numerical aperture *NA* = 0.4, Mitutoyo). Note that this optic collects photons at angles ranging from normal incidence to roughly 23.6^∘^; we simulated the reflectivity and absorptivity within this range to confirm that the angular-dependence of these data is minimal (see Supplementary Fig. 27). We collected the reflected and transmitted light using Ge photodetectors, whose signals we amplified and measured using lock-in amplifiers. We normalized the reflectivity measurements using the reflection from a template-stripped, flat Au sample on Si, whose wavelength-dependent reflectivity we calculated based on literature-obtained indices of refraction^103,104^. To account for source intensity fluctuations, we normalized all experiments by a simultaneous reference measurement of the source beam. Additional information, including a schematic diagram and data processing methods, is provided in Supplementary Note 9.

### Simulations

#### Optical comparison of films

We simulated the optical performance of our sails, as well as that of other published sails, using the transfer-matrix method^18,105^ with optical constants that we either measured directly, extrapolated from our direct measurements (in the case of MoS_2_ in the infrared), or obtained from the literature^38,40,41,106–110^. Additional details, including the optical data that we measured, are included in Supplementary Note 8.

#### Thermally limited power comparison of films

We estimated the maximum power that each film could tolerate up to its thermal limit according to ${\Phi }_{s,max}=2\frac{{\varepsilon }_{e}}{\overline{{\alpha }_{\perp }}}{A}_{\perp }\sigma {T}_{max}^{4}$. Here, *ε*_*e*_ is the effective emissivity at *T* = 1000 K (calculated in the wavelength range *λ* = 2 − 14 μm, see Supplementary Note 10), $\overline{{\alpha }_{\perp }}$ is the laser-band average absorptivity, *A*_⊥_ is the area of the sail perpendicular to the laser beam, *σ* is the Stefan-Boltzmann constant, and *T*_*max*_ is the limiting temperature of the sail^73,111–116^. We estimated the absorptivity for each sail using ${\alpha }_{\lambda,\perp }={\sum }_{i=1}^{n}\frac{4\pi {\kappa }_{\lambda,i}{t}_{f,i}{F}_{i}}{\lambda }$, where *κ*_*λ*,*i*_ is the extinction coefficient of the *i*^th^ layer (*n* total layers) at wavelength *λ*, *t*_*f*,*i*_ is the *i*^th^ layer’s thickness, and *F*_*i*_ is the layer’s material fill factor, estimated using the given geometric pattern of the sail. In our calculations, we used a consistent temperature of *T* = 1000 K to calculate the effective emissivity, which is a reasonable simplification given the other uncertainties in this calculation, such as the temperature dependence of the indices of refraction of the sail materials.

#### Mechanical comparison of films

We used the areal density values provided in each article^16,17,22,26–28,30–32^, if available, or calculated them from the specified film thicknesses using material densities available in the literature^54–56,117–121^. The membrane mechanical robustness, here denoted *℘*, quantifies the ability of a sail film to resist the membrane stresses that occur due to the reflected photon pressure as it accelerates. We calculated these values according to $\wp={\sum }_{i=1}^{n}{G}_{i}{t}_{f,i}{\sigma }_{y,i}$, where *t*_*f*,*i*_ is the *i*^th^ layer’s thickness (*n* total layers), *σ*_*y*,*i*_ is the tensile yield stress of the material’s *i*^th^ layer^56,122–134^, and *G*_*i*_ are geometrical parameters that account for the presence of holes or other architected features in the film layers (see Supplementary Note 11). Our measure reflects the fact that a sail will tear if the stress it experiences is greater than its tensile yield stress, and that the stress the film experiences is inversely proportional to its thickness^18,46^. For our designs, we conservatively set *G* = 1 to examine the robustness of the film in a planar (non-corrugated) state, although the hexagonal structure can be designed to increase compliance to in-plane stresses, thereby increasing the practical membrane mechanical robustness^58^. Values for the films’ membrane mechanical robustness range from about 1 N ⋅ m^−1^ to 10 kN ⋅ m^−1^, and that which we calculated for our fabricated prototype film is 184 N ⋅ m^−1^.

#### Accelerative comparison of films

The maximum relative velocity, denoted *β*_*max*_, achievable by a light sail is a benchmark for its practical accelerative performance. In our simulations, we allowed *β*_*max*_ to depend on each sail’s reflectivity, areal density, and mechanical robustness, but did not consider thermal constraints due to the relatively high absorptivity values of the films. To calculate the *β*_*max*_ values, we simulated the acceleration of a *m*_*p*_ = 1 g payload (tethers plus probe) towed by a *m*_*s*_ = 1 g spherically-curved circular sail with equal radius of curvature and diameter (*s*_*s*_ = *d*_*s*_) having the areal density, mechanical robustness, and spectral reflectivity provided in or derived from each corresponding article^16,17,22,26–28,30–32^. Although we allowed the reflectivity to change with the Doppler-shifted wavelength, we did not consider the impact of the sail curvature on reflectivity and instead assumed that all laser photons were reflected directly backward. For each simulation, we iteratively determined the maximum constant-output laser power that the sail could sustain without tearing due to the photon pressure, up to a maximum of ${\Phi }_{l}^{max}=100$ GW^18^. We allowed each sail to accelerate until the distance it traveled *L* reached the diffraction-limited distance to which the laser output light from a *d*_*l*,*E*_ = 30 km diameter laser array on Earth could be focused; this distance scales as $L \sim \frac{{d}_{l,E}{d}_{s}}{2{\lambda }_{l}}$, where *λ*_*l*_ is the laser output wavelength and *d*_*s*_ is the sail diameter^18^. Additional details can be found in Supplementary Notes 12 and 14.

#### Optimization of corrugated nanolaminate design

To determine the film thicknesses that maximized *β*_*max*_ within our design space, we numerically simulated a range of combinations of Al_2_O_3_ and MoS_2_ thicknesses (*t*_*A*_ and *t*_*M*_, respectively, with identical top and bottom alumina thicknesses). Anticipating future improvements in microfabrication, we used literature data for the indices of refraction of MoS_2_ and Al_2_O_3_^38,41^ rather than the optical properties that we measured in-house, and we adopted a tensile yield strength for MoS_2_ that is 10% of the single-layer crystalline value^131^; our estimate is reasonable given comparable data for a similar material, MoSe_2_^135^. For simplicity, we selected a single hexagonal corrugation pattern for this optimization, with hexagon diameter *d*_*h*_ = 70 μm, trench width *w*_*t*_ = 2 μm, and trench height *h*_*t*_ = 3 μm. More details are available in Supplementary Note 13.

#### Thermal performance of sail

We simulated the thermal performance of our proposed optimized film by solving the energy equation for laser photon energy absorbed and thermal energy radiated away. We did this by stepping through the acceleration phase from *β* = 0 to *β* = 0.2 and balancing the energy with a steady-state assumption, which is reasonable given the small thermal inertia (i.e., low mass) of the sail compared to the enormous incoming laser power. We obtained the required optical properties from Munkhbat et al.^38^, Lingart, Petrov, and Tikhonova^39^, and Querry^40^, except, as mentioned in the text, we specified the extinction coefficient of MoS_2_ to be *κ* = 10^−8^ at wavelengths equal to and longer than the laser wavelength. Additional details can be found in Supplementary Note 15.

## Supplementary information

Supplementary Information

Transparent Peer Review file

## Data Availability

Relevant data supporting the key findings of this study are available within the article and the Supplementary Information file. All raw data generated during the current study are available from the corresponding authors upon request.

## Code availability

Code supporting this study was written for MATLAB R2024b and/or Python and is available at https://github.com/souperman1985/starshot.
