# Supplementary material for: Experimental demonstration of corrugated nanolaminate films as reflective light sails
Source: Nat Commun. 2026 May 26;17:6874. doi: 10.1038/s41467-026-73414-4 (PMC13389247; doi:10.1038/s41467-026-73414-4)
Supplement: Supplementary file 1 — Supplementary Information [file 41467_2026_73414_MOESM1_ESM.pdf]

Supplementary Information: Experimental demonstration of corrugated nanolaminate films as reflective light sails

Matthew F. Campbell[‡1], Pawan Kumar[‡2], Jason Lynch[2], Ramon Gao[3], Adam Alfieri[2], John Brewer[4], Thomas J. Celenza[1], Mohsen Azadi[1,5], Michael D. Kelzenberg[3], Eric Stach[5,6], Aaswath P. Raman[4], Harry A. Atwater[3], Igor Bargatin[*1], and Deep Jariwala[†2]

[‡]*These authors contributed equally to this work.*
[1]*Department of Mechanical Engineering and Applied Mechanics, University of Pennsylvania, Philadelphia PA, USA 19104*
[2]*Department of Electrical and Systems Engineering, University of Pennsylvania, Philadelphia PA, USA 19104*
[3]*Thomas J. Watson Laboratory of Applied Physics, California Institute of Technology, Pasadena CA, USA 91125*
[4]*Department of Materials Science and Engineering, University of California at Los Angeles, Los Angeles CA, USA 90095*
[5]*Singh Center for Nanotechnology, University of Pennsylvania, Philadelphia PA, USA 19104*
[6]*Department of Materials Science and Engineering, University of Pennsylvania, Philadelphia PA, USA 19104*

05-07-2026

Supplementary Figures

[*]Corresponding author. Email: bargatin@seas.upenn.edu
[†]Corresponding author. Email: dmj@seas.upenn.edu

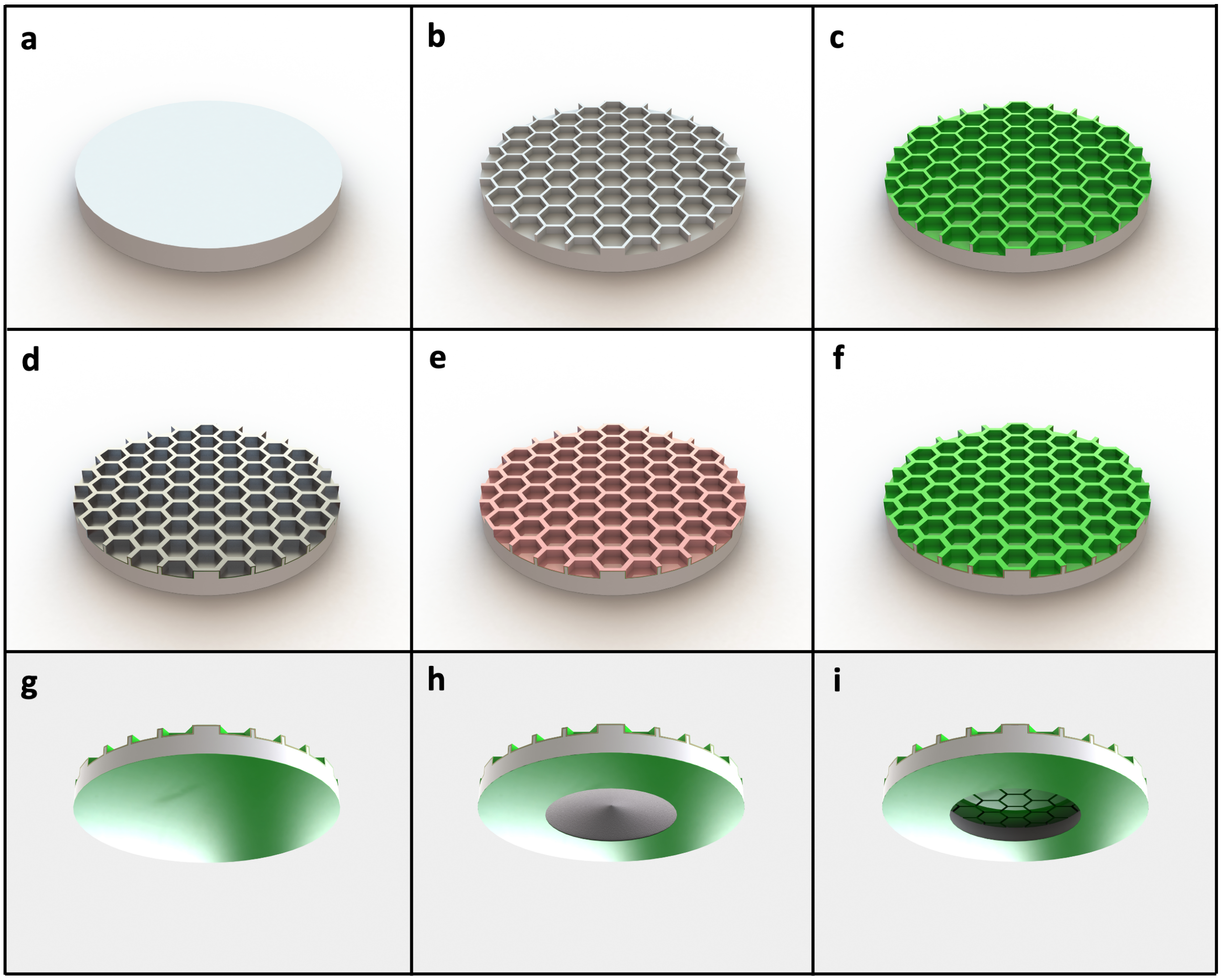

Supplementary Figure 1. | Fabrication steps for sail film prototypes in the protruding rib configuration. See also Figure 3(a) in the main article. (**a**) Begin with a plain 200-μm-thick double-side-polished Si wafer. (**b**) Form hexagon shapes/ribs using photolithography and deep reactive ion etching. (**c**) Use atomic layer deposition to produce the lower conformal Al_2O_3 film. (**d**) Conformally sputter Mo. (**e**) Sulfurize the Mo in a high-temperature tube furnace. (**f**) Use atomic layer deposition to produce the upper conformal Al_2O_3 film. (**g**) Invert the wafer. Note that the backside was coated in Al_2O_3 in steps (**c**) and (**f**). (**h**) Use laser micromachining to cut through the backside Al_2O_3 and remove a majority of the Si substrate. (**i**) Use XeF_2 gaseous etching to remove the rest of the Si mold. The film is now suspended.

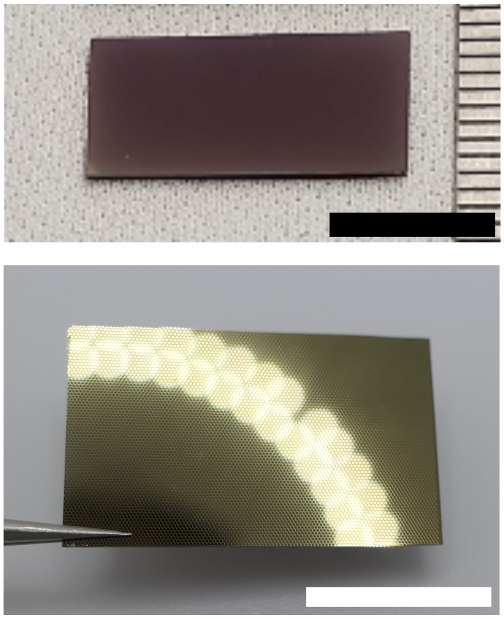

Supplementary Figure 2. | Photographs of Si chips with films attached. (**a**) 45-nm-thick sample of MoS_2 on flat (non-corrugated) substrate. (**b**) Prototype composite film on trench-corrugated Si chip (held on the left by tweezers). The corrugation in this particular embodiment is faintly visible to the naked eye. Sample dimensions: $d_h \approx 154$ µm, $w_t \approx 30$ µm, $h_t \approx 10$ µm, $t_{A,b} \approx 21$ nm, $t_M \approx 60$ nm, $t_{A,t} \approx 51$ nm. Scale bars: (**a**) 10 mm, (**b**) 10 mm.

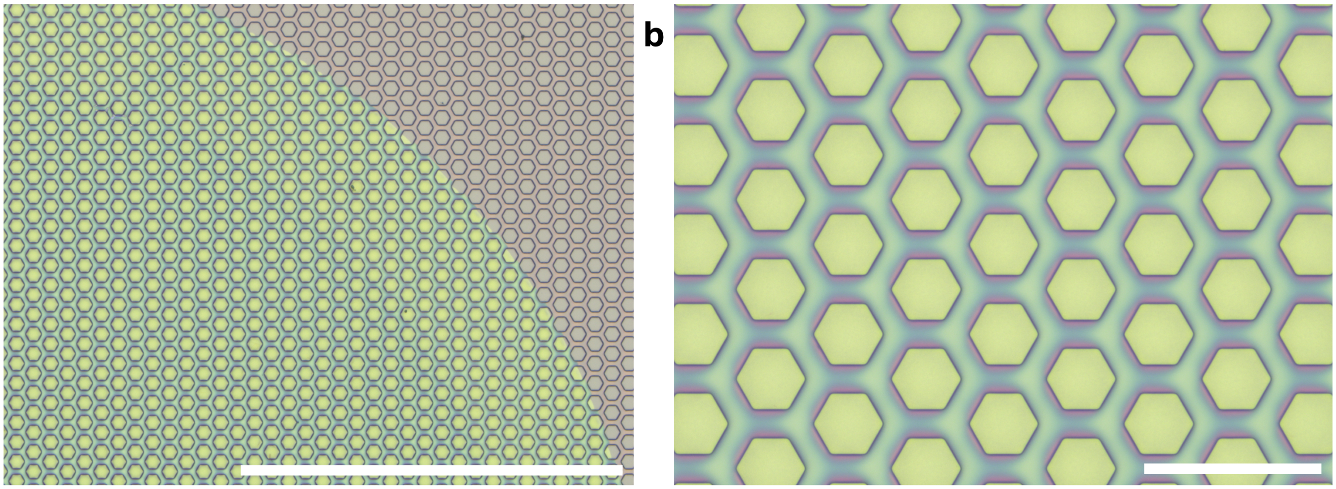

Supplementary Figure 3. | Additional micrographs of the fully suspended prototype film (indented trenches configuration) shown in Figure 1(b) of the main article. (**a**) Perimeter of suspended region. Red area is on Si substrate and green area has substrate removed. (**b**) Enlarged view of suspended area. Sample dimensions: $d_h \approx 36$ µm, $w_t \approx 15$ µm, $h_t \approx 10$ µm, $t_{A,b} \approx 15$ nm, $t_M \approx 75$ nm, $t_{A,t} \approx 53$ nm. Scale bars: (**a**) 1000 µm and (**b**) 100 µm.

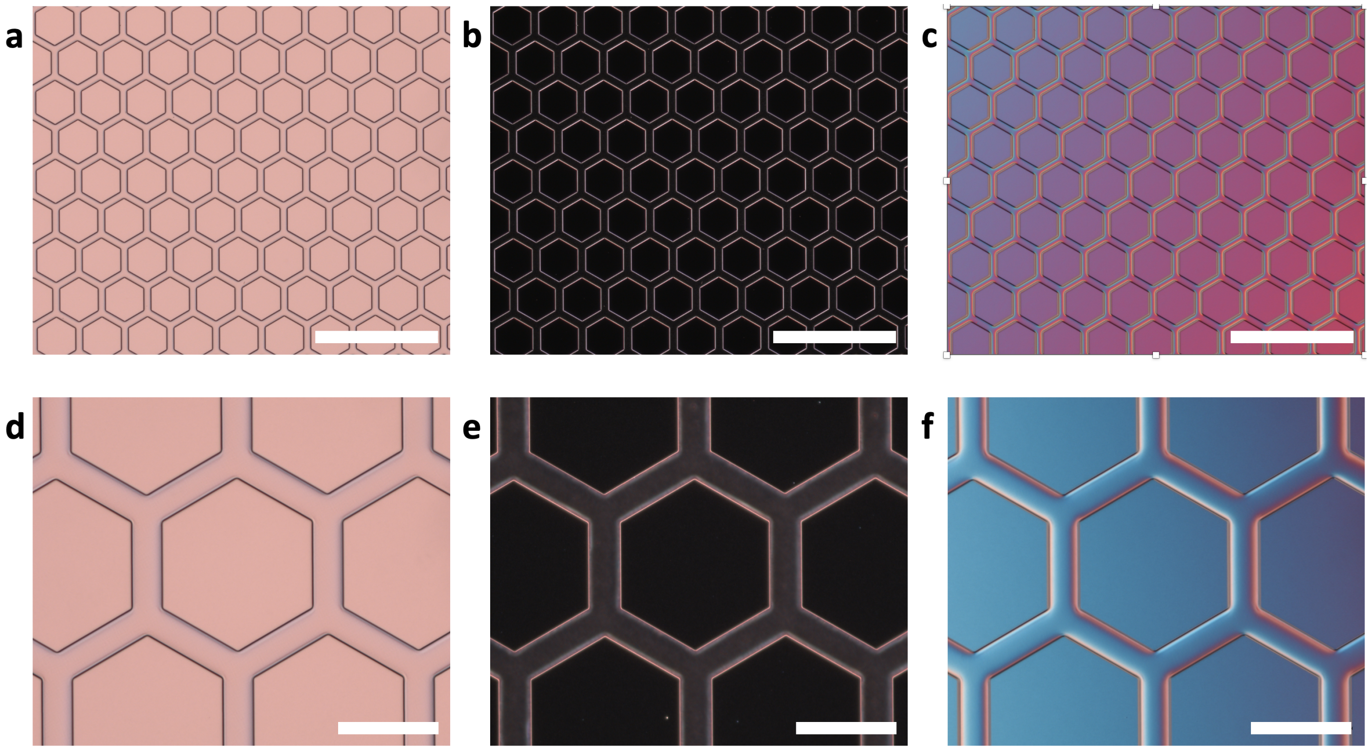

Supplementary Figure 4. | Micrographs of a corrugated composite film on a Si substrate. This is the same sample shown in Supplementary Figure 2(b). The pattern features hexagonal trenches etched down into the Si. (**a**, **d**): Brightfield images. (**b**, **e**): Darkfield images. (**c**, **f**): Reflected differential image contrast (DIC) images. Images obtained on a Zeiss Axio Imager M2m Microscope. Scale bars: (**a**, **b**, **c**) 500 μm and (**d**, **e**, **f**) 100 μm.

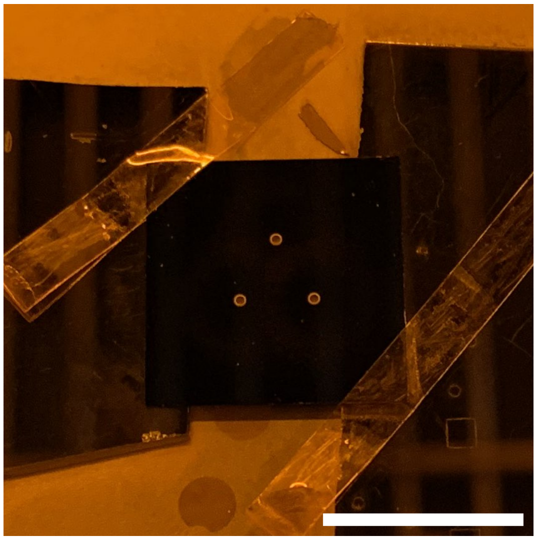

Supplementary Figure 5. | Results of laser etching. Photograph shows 12-mm square Si chip with three 500 μm laser-drilled holes in its back side. Scale bar: 10 mm.

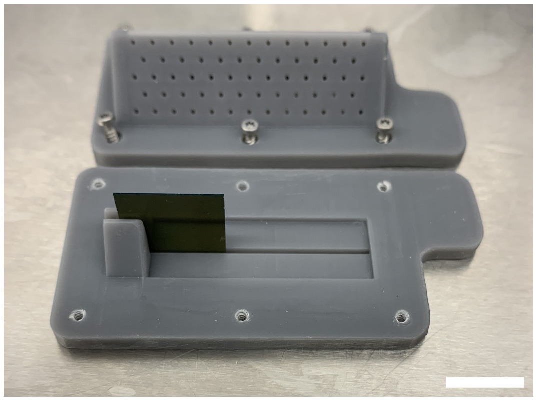

Supplementary Figure 6. | Preparation for gaseous etching. Photograph shows fixture used to hold chips during XeF_2 etching. The vertical orientation of the chip ensures that gas encounters both of its sides at equal pressures. Scale bar: 10 mm.

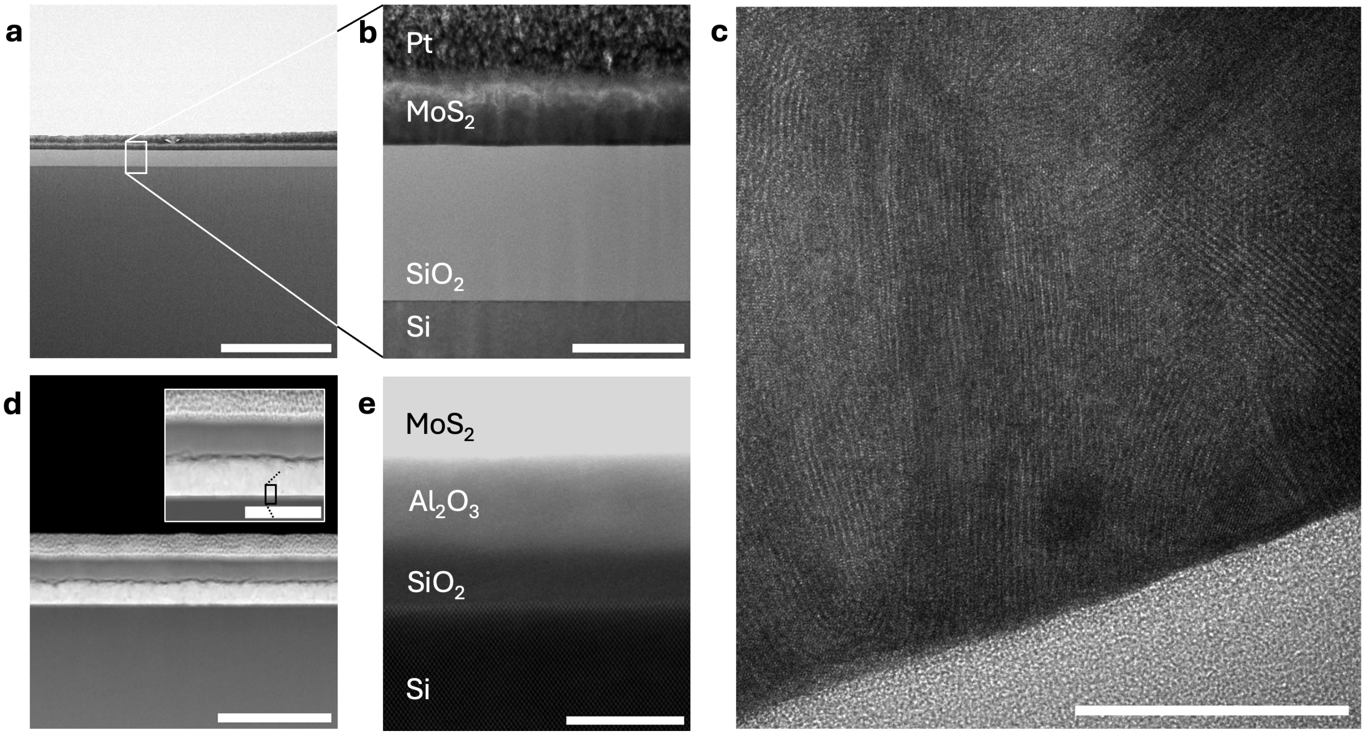

Supplementary Figure 7. | Cross-sectional transmission electron microscopy (TEM) images of MoS_2 films. Film thickness is about 75-nm. (**a**) Micrograph of MoS_2 on SiO_2/Si substrate. (**b**) Enlargement of panel (**a**) with layers labeled. The Pt is used as a protective layer during the microscopy process. (**c**) Micrograph showing layered structure of MoS_2. (**d**) Micrograph obtained by imaging MoS_2 directly on Al_2O_3 ribs of hexagonal mold pattern. Inset shows enlargement of important layers. (**e**) Further enlargement from the inset of panel (**d**) as indicated by black rectangle. The native oxide (SiO_2) and Si mold substrate can be seen. Scale bars: (**a**) 2 μm, (**b**) 200 nm, (**c**) 20 nm, (**d**) 500 nm, inset of (d): 200 nm, (**e**) 10 nm.

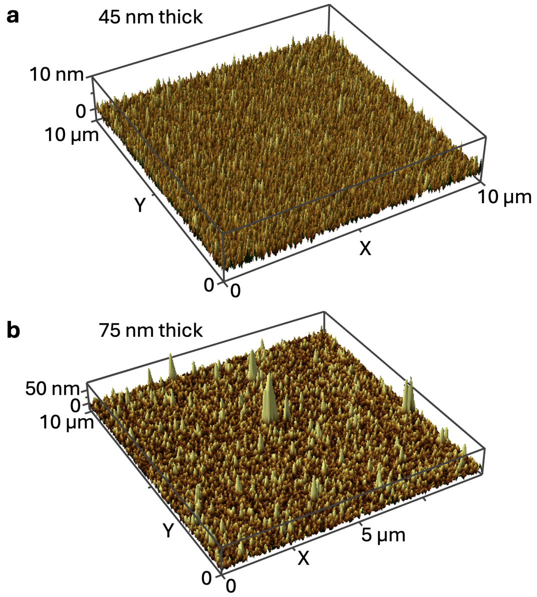

Supplementary Figure 8. | Atomic force microscopy (AFM) scans of MoS_2 samples on SiO_2/Si substrates. (**a**) 45 nm thick sample with root-mean-square (RMS) roughness ~ 2 nm. (**b**) 75 nm thick sample with RMS roughness ~ 5 nm.

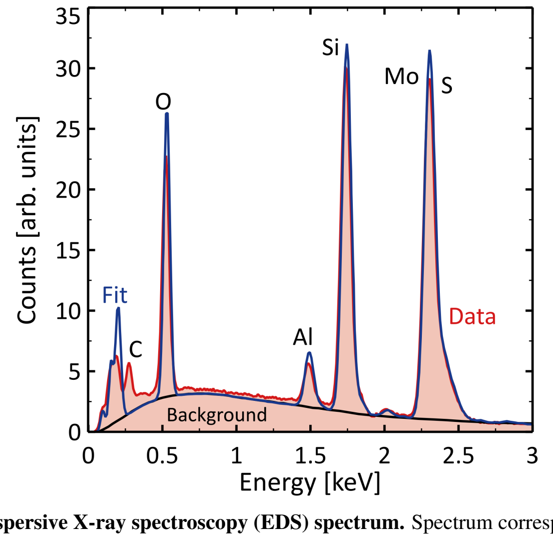

Supplementary Figure 9. | Energy dispersive X-ray spectroscopy (EDS) spectrum. Spectrum corresponds to Figure 3(b) in the main article.

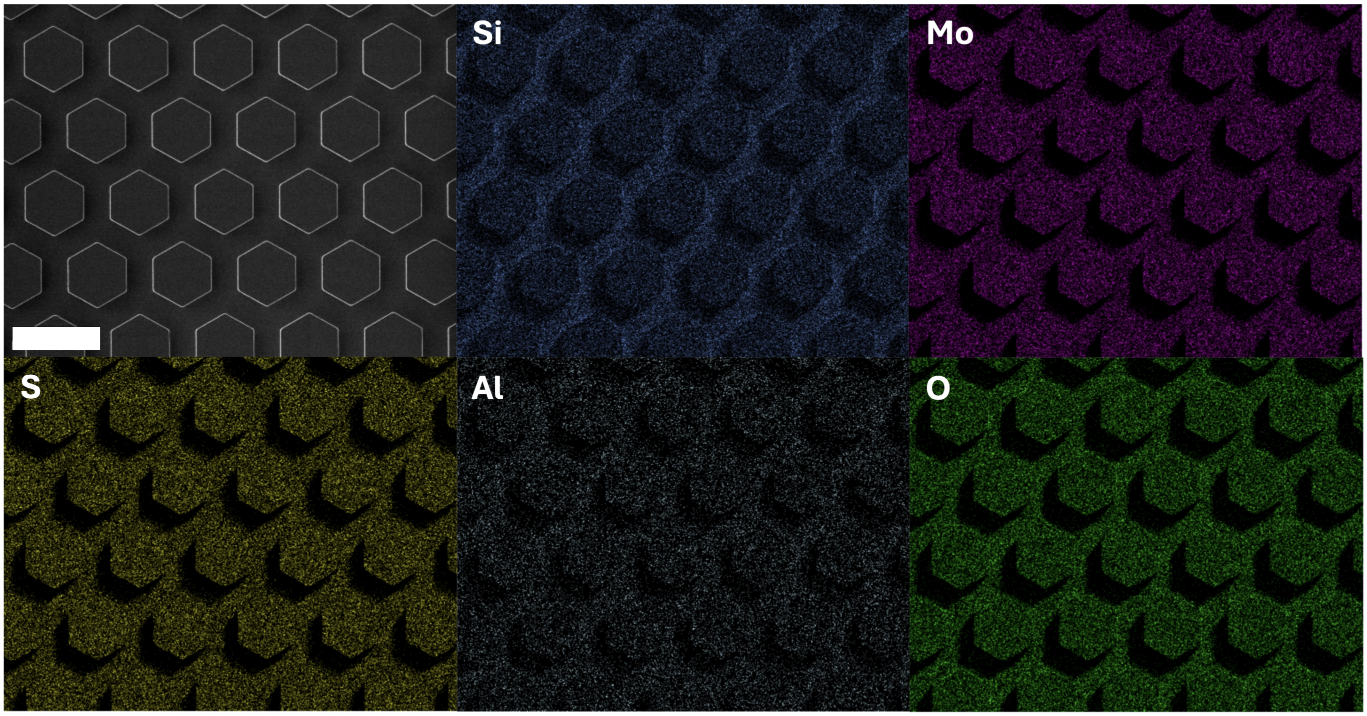

Supplementary Figure 10. | SEM and EDS images obtained normal to the film surface. Depicted is a corrugated nanolaminate film on Si substrate etched in the indented trench configuration, showing elemental composition in several unit cells. See also Supplementary Figure 11. Scale bar: 50 μm.

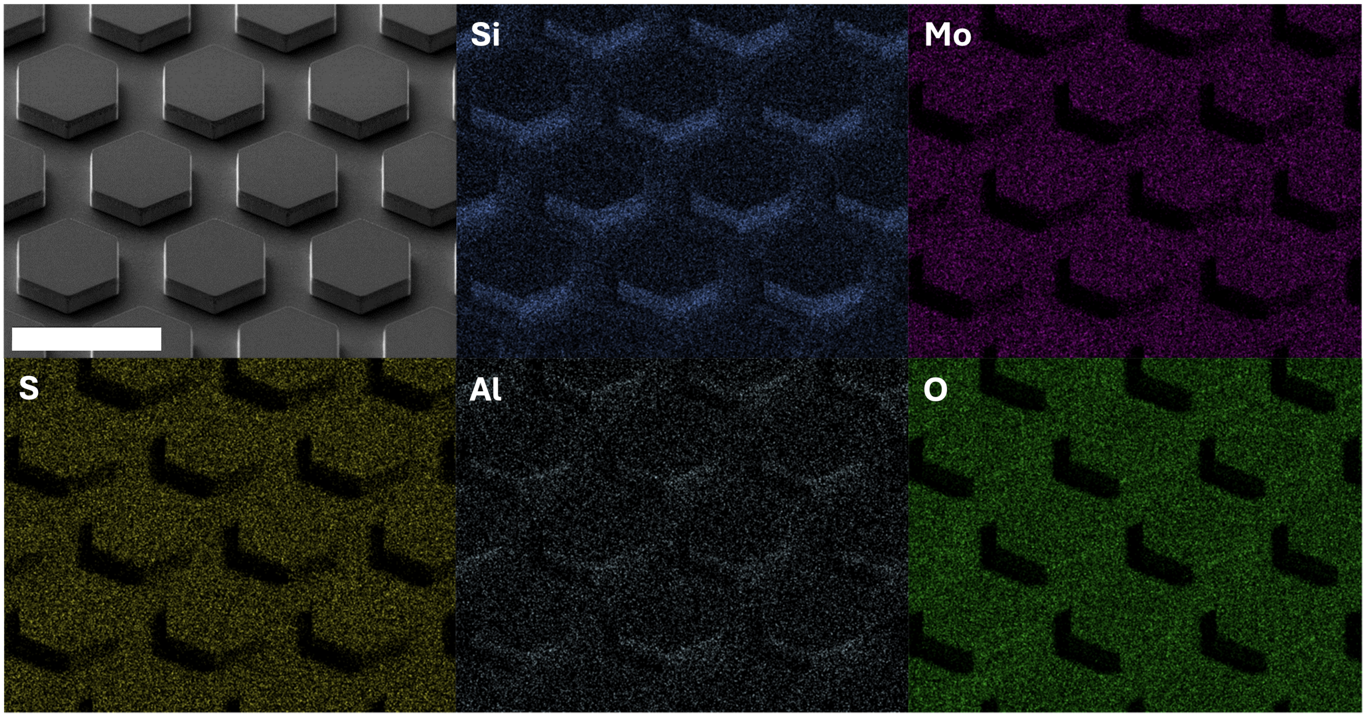

Supplementary Figure 11. | SEM and EDS images obtained at 45° to the film surface. See also Supplementary Figure 10. Scale bar: 50 μm.

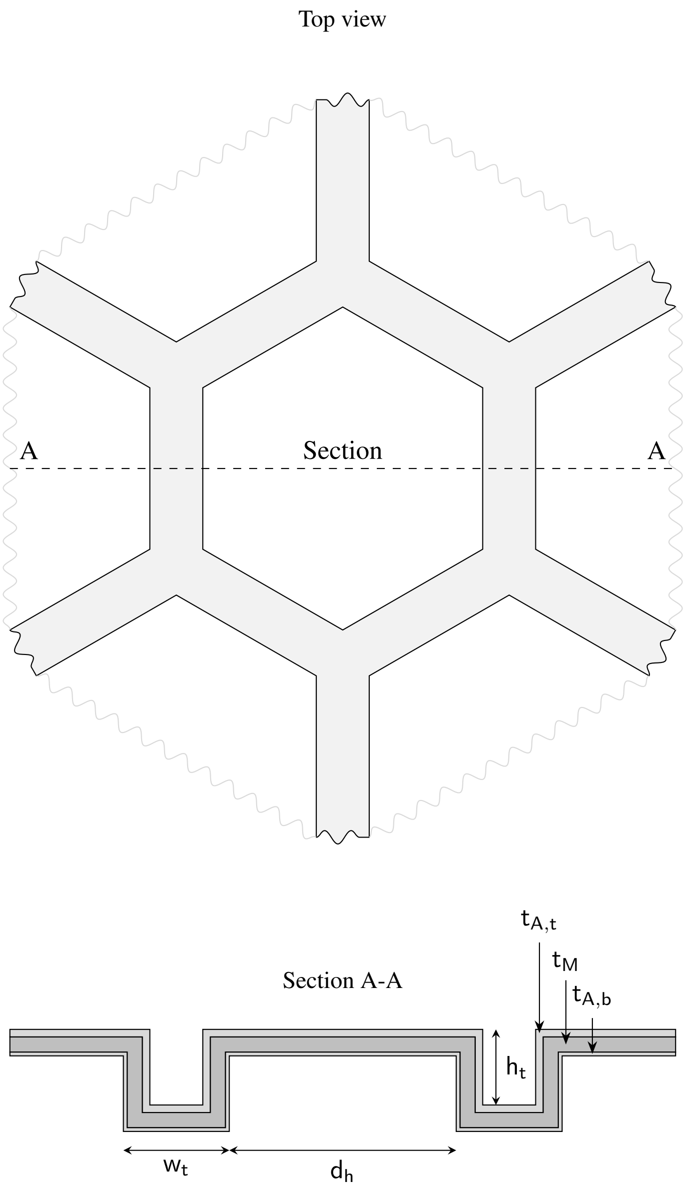

Supplementary Figure 12. | Schematic diagram of a hexagonally-corrugated film in the indented trench configuration. Upper graphic presents a top view; lower graphic provides a section view showing the film thicknesses $t_{A,b}$ (bottom), t_M (middle), and $t_{A,t}$ (top), with thickness ratios 1:4:2. We have exaggerated the dimensions to provide clarity, and, in the top view, shaded the lower trench regions. The hexagon diameter d_h, trench height h_t, and trench width w_t are defined based on the dimensions of the Si mold.

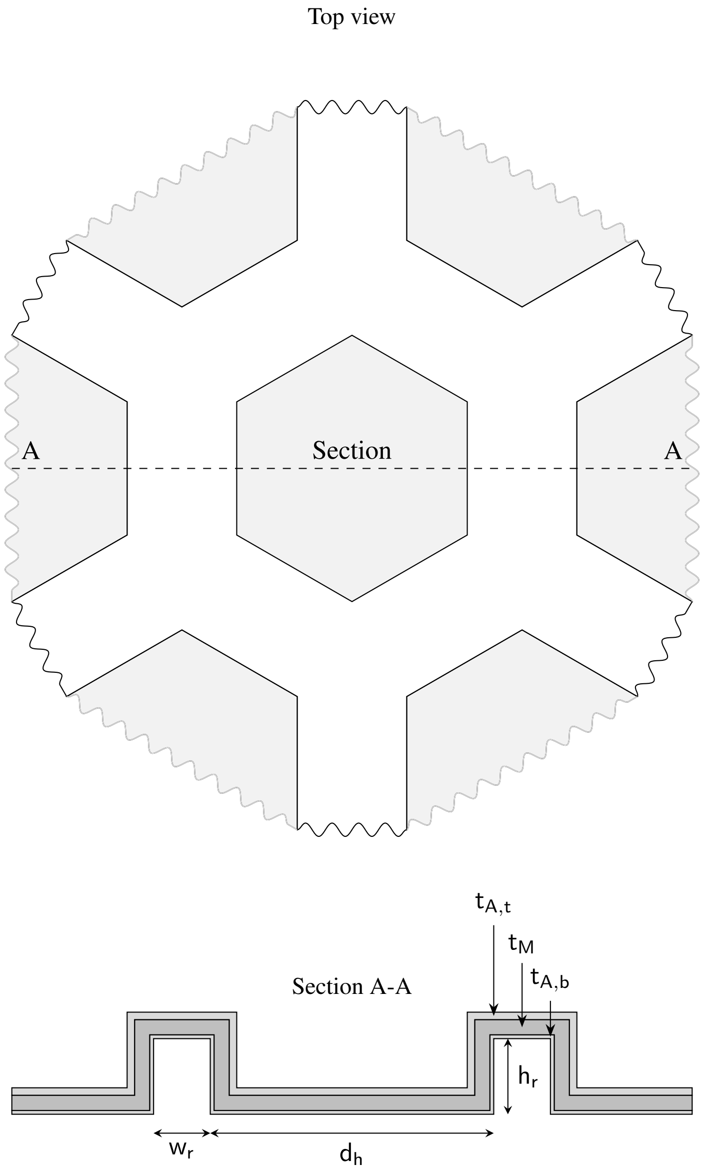

Supplementary Figure 13. | Schematic diagram of a hexagonally-corrugated film in the protruding rib configuration. Upper graphic presents a top view; lower graphic provides a section view showing the film thicknesses $t_{A,b}$ (bottom), t_M (middle), and $t_{A,t}$ (top), with thickness ratios 1:4:2. We have exaggerated the dimensions to provide clarity, and, in the top view, shaded the lower hexagon regions. The hexagon diameter d_h, rib height h_r, and rib width w_r are defined based on the dimensions of the Si mold.

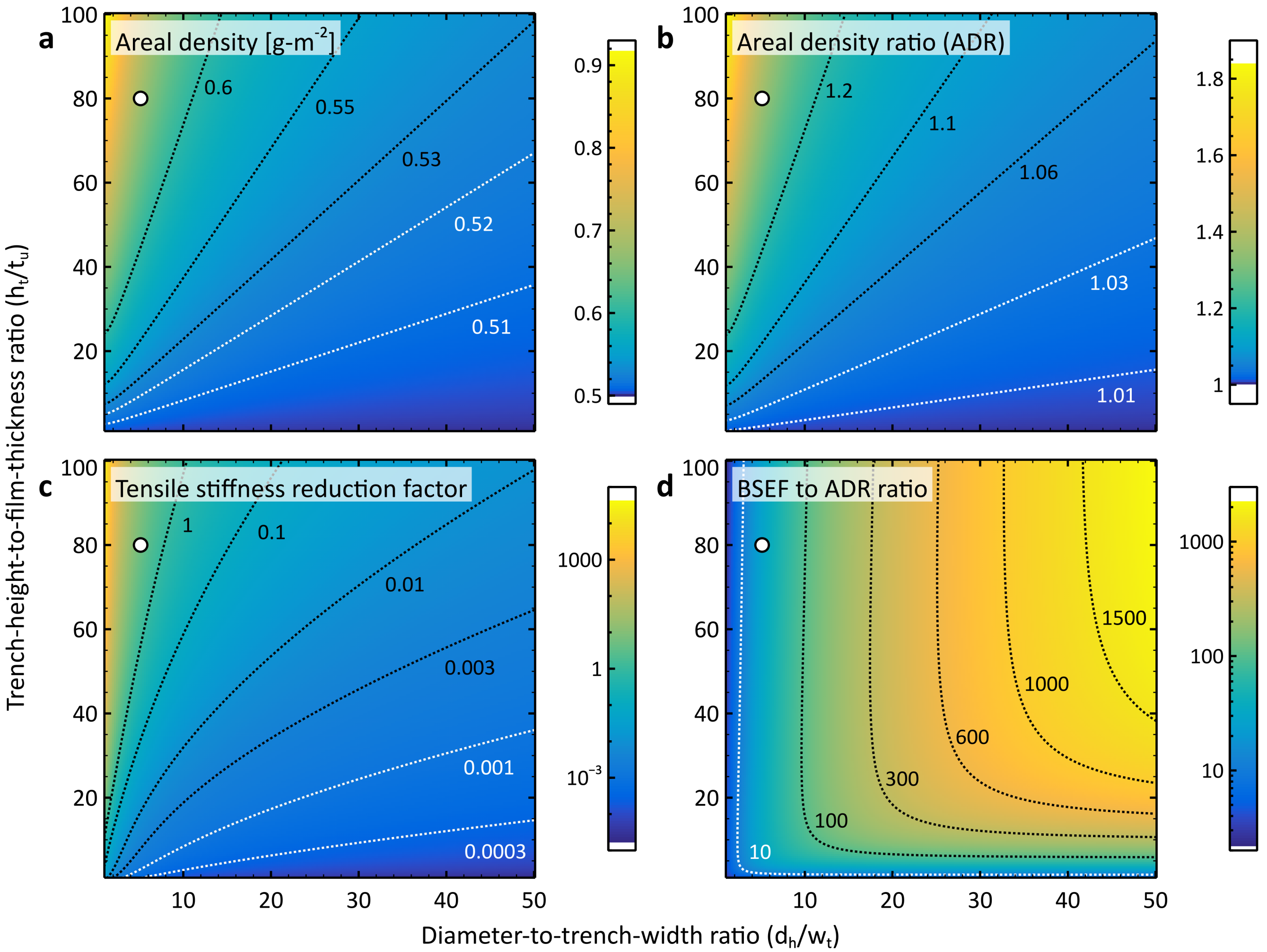

Supplementary Figure 14. | **Mechanical property calculations for fabricated prototype film.** Panel (**a**) shows the corrugated areal density $\rho_{a,c,t}$ for hexagonally corrugated films (indented trench configuration) with $t_{A,b} \approx 21$ nm (bottom Al_2O_3 thickness), $t_M \approx 53$ nm (MoS_2), and $t_{A,t} \approx 51$ nm (top Al_2O_3), and panel (**b**) shows the areal density ratio (ADR): the ratio of the areal density of a corrugated film to that of a flat/planar film with the same nanolaminate thicknesses (areal density $\rho_{a,p} \approx 0.5\ \mathrm{g{\cdot}m^{-2}}$). Panel (**c**) shows the tensile stiffness reduction factor $\mathbb{T}$ and panel (**d**) provides the ratio of the bending stiffness enhancement factor $\mathbb{B}$ (Figure 3(e) in the main article) to the areal density ratio (panel (b) of this figure). The fabricated prototype design, featuring $d_h \approx 77$ µm, $w_t \approx 15$ µm, $h_t \approx 10$ µm, $\rho_{a,c,t} \approx 0.7\ \mathrm{g{\cdot}m^{-2}}$, $\mathbb{B} \approx 38$, and $\mathbb{T} \approx 2.4$, is shown with the white circle. In this case, the value $\mathbb{T} > 1$ indicates no reduction in the tensile stiffness relative to a planar (non-corrugated) film. See also Supplementary Figure 15.

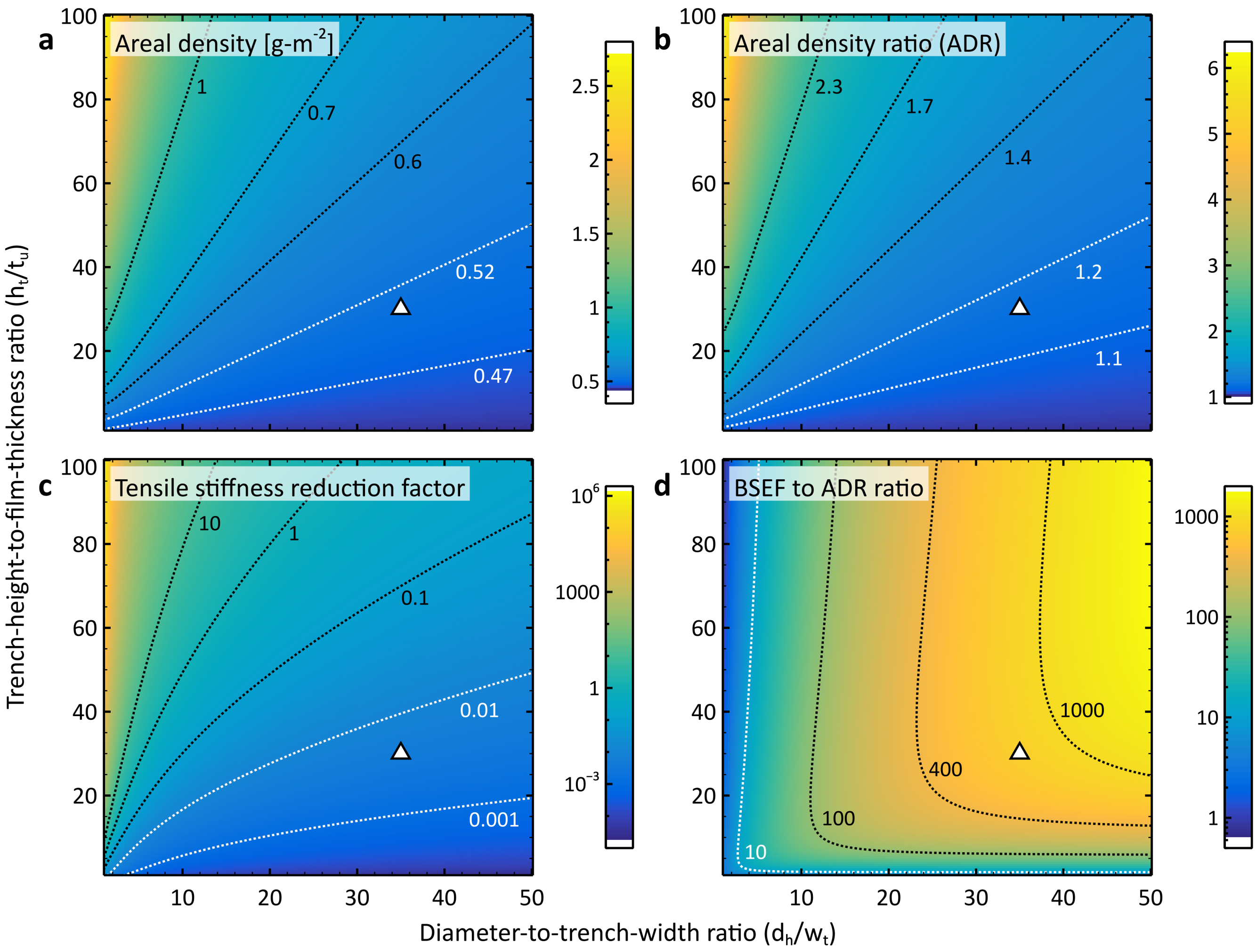

Supplementary Figure 15. | Mechanical property calculations for proposed optimized film. Panel (**a**) shows the corrugated areal density $\rho_{a,c,t}$ for hexagonally corrugated films (indented trench configuration) with $t_{A,b} = t_{A,t} \approx 19$ nm and $t_M \approx 63$ nm, and panel (**b**) shows the areal density ratio (ADR): the ratio of the areal density of a corrugated film to that of a flat/planar film with the same nanolaminate thicknesses (areal density $\rho_{a,p} \approx 0.4\ \mathrm{g \cdot m^{-2}}$). Panel (**c**) shows the tensile stiffness reduction factor $\mathbb{T}$ and panel (**d**) provides the ratio of the bending stiffness enhancement factor $\mathbb{B}$ (Figure 3(e) in the main article) to the areal density ratio (panel (b) of this figure). The proposed optimized design, featuring $d_h = 70$ µm, $w_t = 2$ µm, $h_t = 3$ µm, $\rho_{a,c,t} \approx 0.5\ \mathrm{g \cdot m^{-2}}$, $\mathbb{B} \approx 890$, and $\mathbb{T} \approx 0.0033$, is shown with the white triangle. See also Supplementary Figure 14.

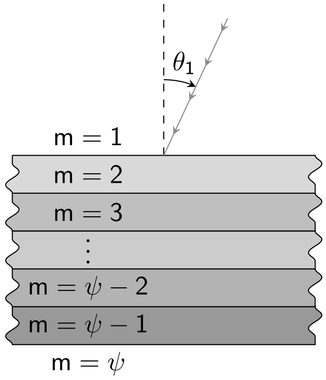

Supplementary Figure 16. | Film layers in the transfer-matrix method. The film is shown with ψ layers, including the space on both sides of the structure. Each mth layer has a complex index of refraction $\mathfrak{n}_m = n_m + \mathrm{i}\kappa_m$, a thickness t_m, and an angle of incidence (the polar angle) relative to the normal direction θ_m. In the case of a sail in the vacuum of space, the index of refraction of the first and ψ^{th} layers is $\mathfrak{n}_1 = n_1 + \mathrm{i}\kappa_1 = \mathfrak{n}_\psi = n_\psi + \mathrm{i}\kappa_\psi = 1 + 0\mathrm{i}$ and the thicknesses t_1 and t_ψ are semi-infinite. The azimuthal angle ϕ (not shown) quantifies the rotation about the vertical dashed line.

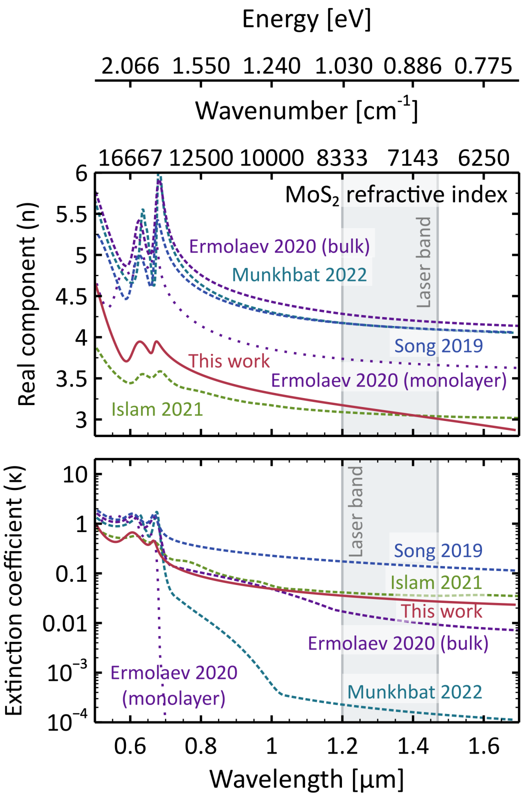

Supplementary Figure 17. | Index of refraction measurements for MoS_2 films in the literature. Comparison is provided of the measured index of refraction $\mathfrak{n} = n + i\kappa$ for our MoS_2 film with those reported by Song *et al.* [1], Ermolaev *et al.* [2], Islam *et al.* [3], and Munkhbat *et al.* [4]. The bulk sample of Ermolaev *et al.* [2] was roughly $1\ \mathrm{mm}$ thick. Here and elsewhere, the shaded gray range denotes the Doppler-shifted wavelength range corresponding to a final relative velocity of $\beta_f = 0.2$: $\lambda = \langle 1.2, 1.4697 \rangle$ µm.

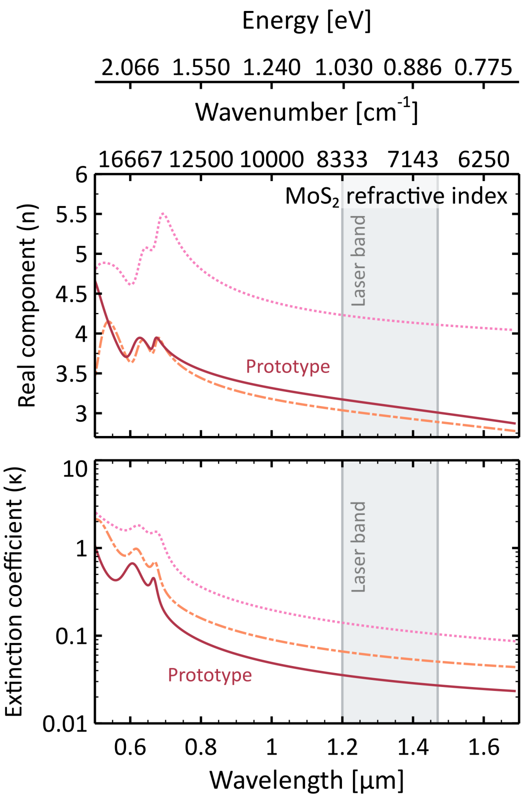

Supplementary Figure 18. | Index of refraction measurements for three MoS_2 films fabricated in-house. The record labeled "Prototype" is also shown in Supplementary Figure 17.

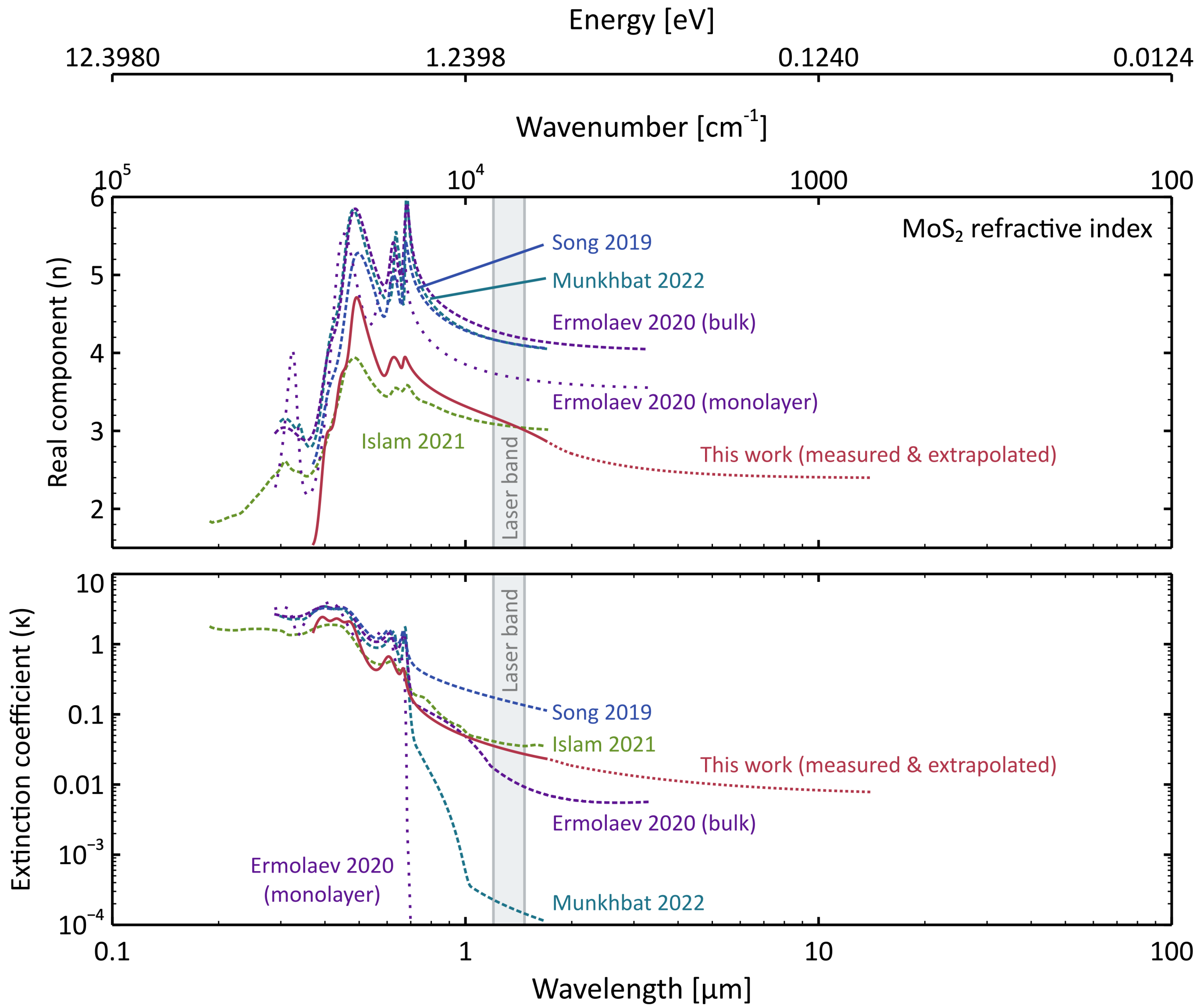

Supplementary Figure 19. | Index of refraction of MoS_2 films over an extended wavelength range. Data are obtained from Song *et al.* [1], Ermolaev *et al.* [2], Islam *et al.* [3], and Munkhbat *et al.* [4]. The extrapolation of our measured data is shown in fine dots.

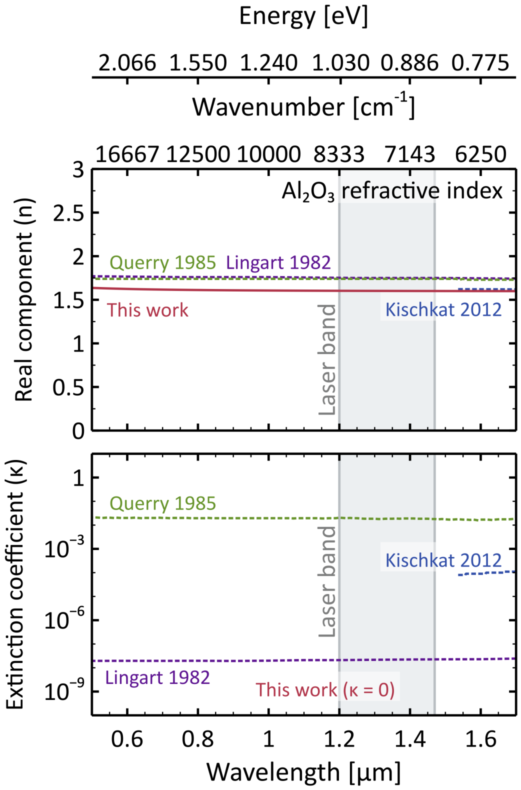

Supplementary Figure 20. | Index of refraction measurements for Al_2O_3 films in the literature. Comparison is provided of the measured index of refraction $\mathfrak{n} = n + i\kappa$ for our Al_2O_3 film with those reported by Lingart, Petrov, and Tikhonova [5] at ($T = 300$ K), Querry [6], and Kischkat *et al.* [7]. Our measurements did not reveal any extinction from the alumina ($\kappa = 0$).

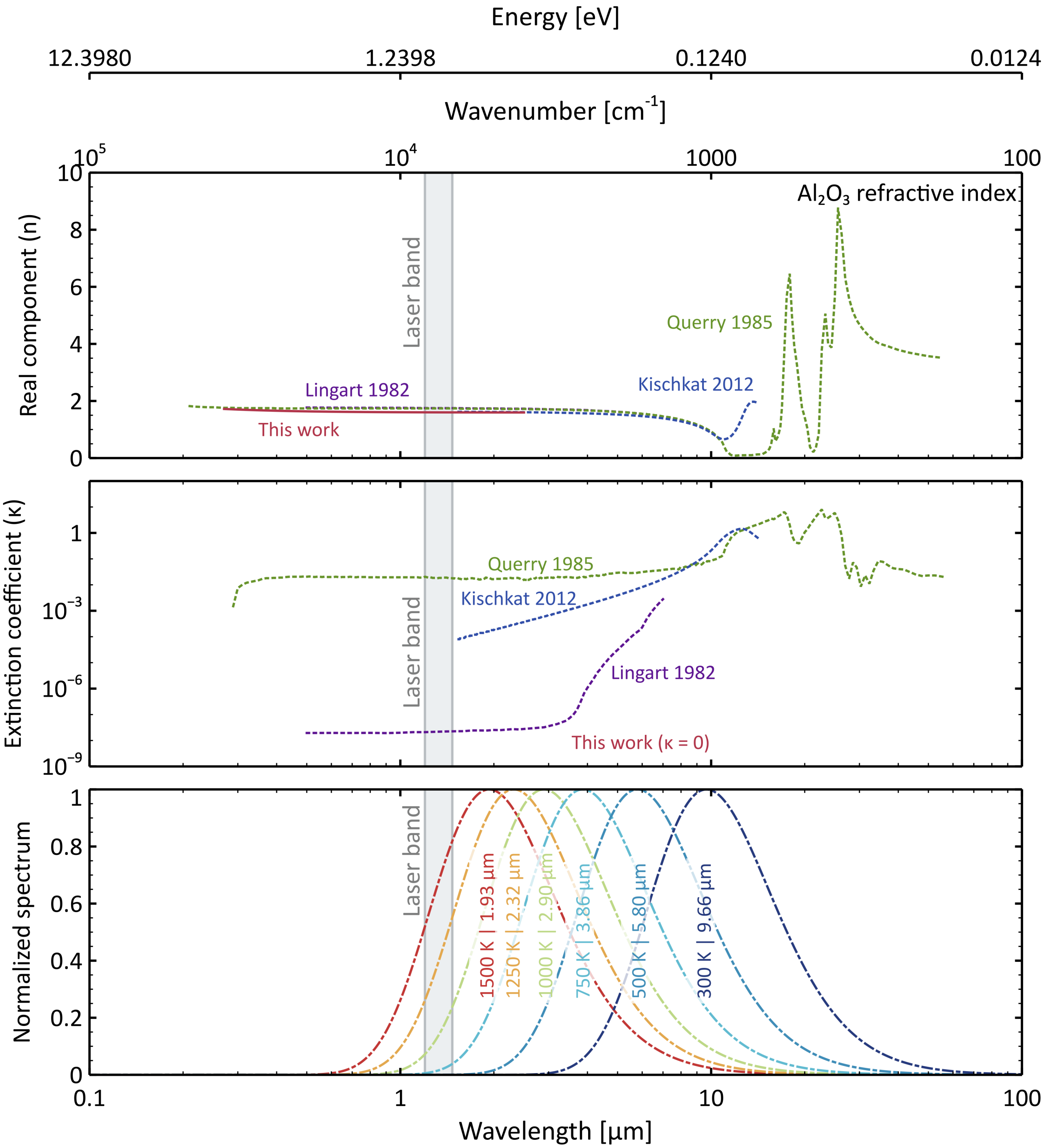

Supplementary Figure 21. | Index of refraction of Al_2O_3 films over an extended wavelength range. Top and middle: Index of refraction. Comparison is given between our measurements and those reported by Lingart, Petrov, and Tikhonova [5], Querry [6], and Kischkat *et al.* [7]. Our measurements did not reveal any extinction from the alumina ($\kappa = 0$). Bottom: Normalized Planck black-body emission spectrum parametrized by wavelength (Equation 84) at several temperatures. The peak wavelength in each distribution according to Wien's displacement law is provided as well. Note that the peaks would have different positions (at longer wavelengths) if the distribution was parameterized by frequency [8]. Though the peaks at higher temperatures correspond to wavelengths where the extinction coefficient of Al_2O_3 is not as large, the overall exitance can still be significant because the peak of the black-body radiation curve increases dramatically in magnitude (curves in this plot are normalized to unity).

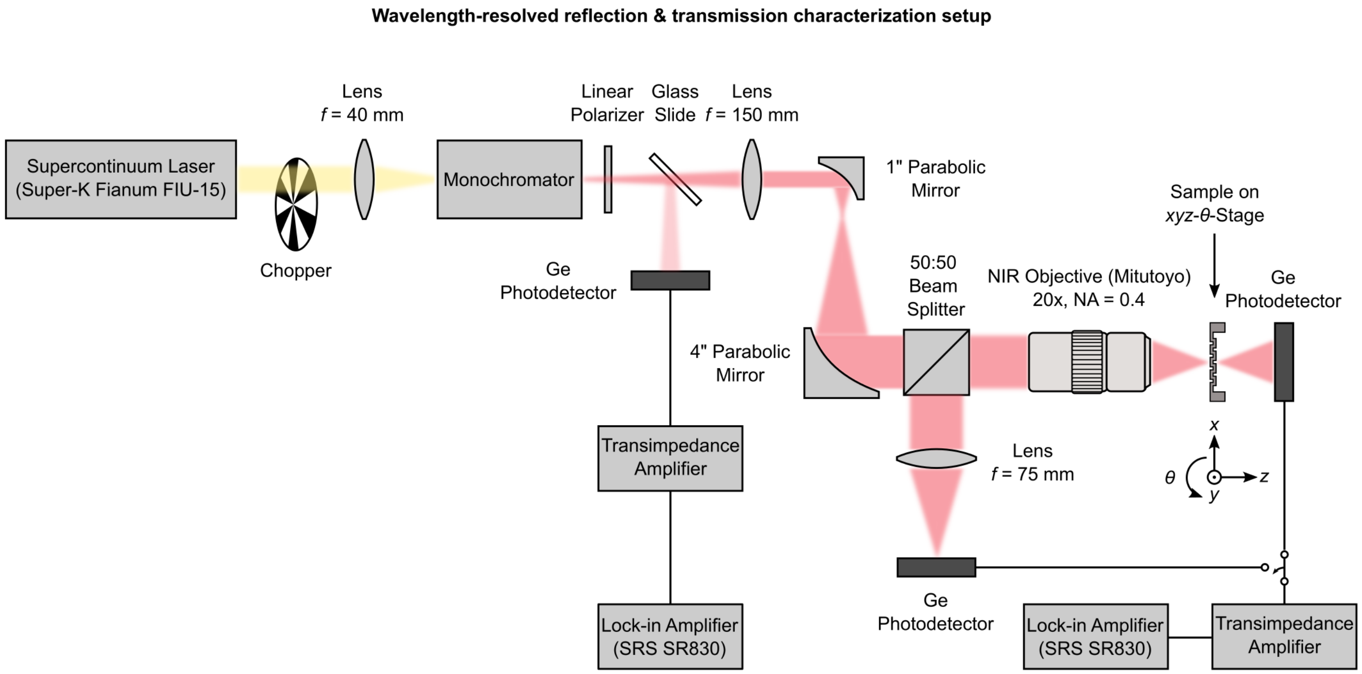

Supplementary Figure 22. | Schematic diagram showing experimental laser setup for reflection and transmission measurements.

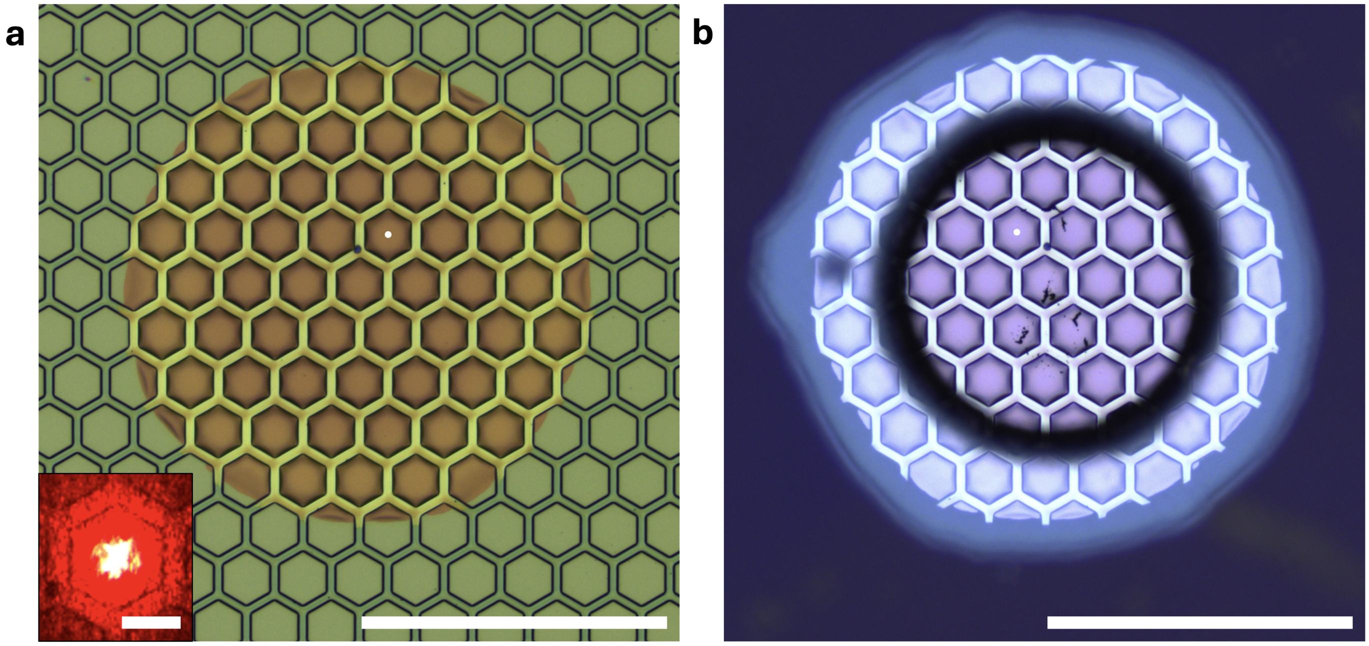

Supplementary Figure 23. | Micrographs showing suspended film used for laser measurements. (**a**) Front/top side, showing indented trenches. Inset: laser spot, focused on the center of a single hexagon. (**b**) Back/bottom side. The small white dots show the same hexagon illuminated with the laser spot in the inset. The dark residue on the backside is likely residual SiO_2 debris created in the laser etching fabrication step. The backside image shows evidence of lateral etching that occurred during the XeF_2 release fabrication step, in that the original laser-drilled hole in the backside had a diameter of roughly 500 µm, but the final suspended diameter is closer to 770 µm. Fabricated prototype film dimensions: $d_h \approx 77$ µm, $w_t \approx 15$ µm, $h_t \approx 10$ µm, $t_{A,b} \approx 21$ nm (bottom Al_2O_3 thickness), $t_M \approx 53$ nm (MoS_2), $t_{A,t} \approx 51$ nm (top Al_2O_3), $\rho_{a,c,t} \approx 0.7\ \mathrm{g{\cdot}m^{-2}}$ (corrugated film areal density). Scale bars: (**a**) 500 µm, inset of (a): 50 µm, (**b**) 500 µm.

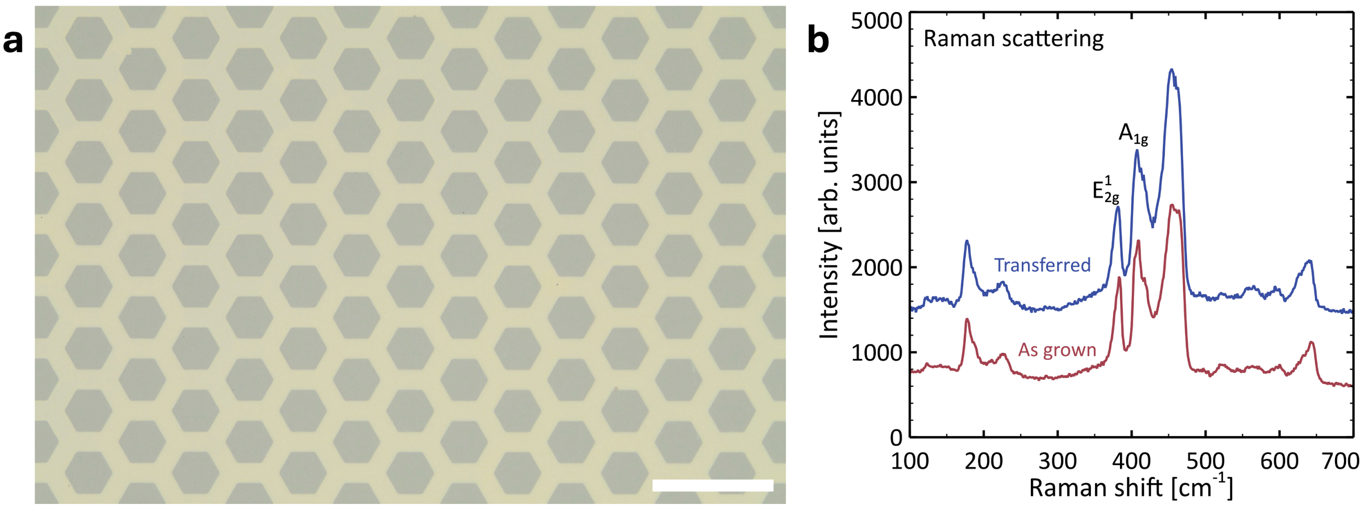

Supplementary Figure 24. | Visualization and characterization of wet chemical transfer prototype film. (**a**) Optical micrograph showing prototype film on Si substrate (indented trench configuration) with MoS_2 film (originally grown on a flat substrate) wet chemical transferred on top [9]. (**b**) Raman scattering measurements of a MoS_2 film on a Si substrate and after transfer to a Al_2O_3-coated corrugated Si substrate. Sample dimensions: $d_h \approx 36$ µm, $w_t \approx 15$ µm, $h_t \approx 10$ µm. Scale bar: (**a**) 100 µm.

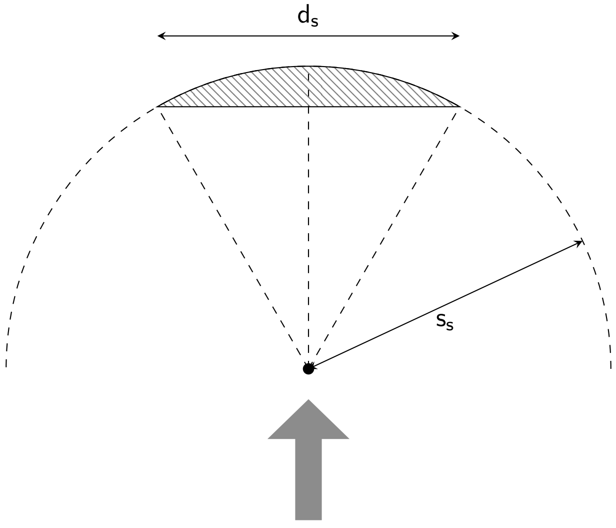

Supplementary Figure 25. | Schematic diagram of a spherically curved circular sail. The perspective provided is a side view, and the sail is shaded. The sail's diameter is equal to its spherical radius of curvature: $d_s = s_s$.

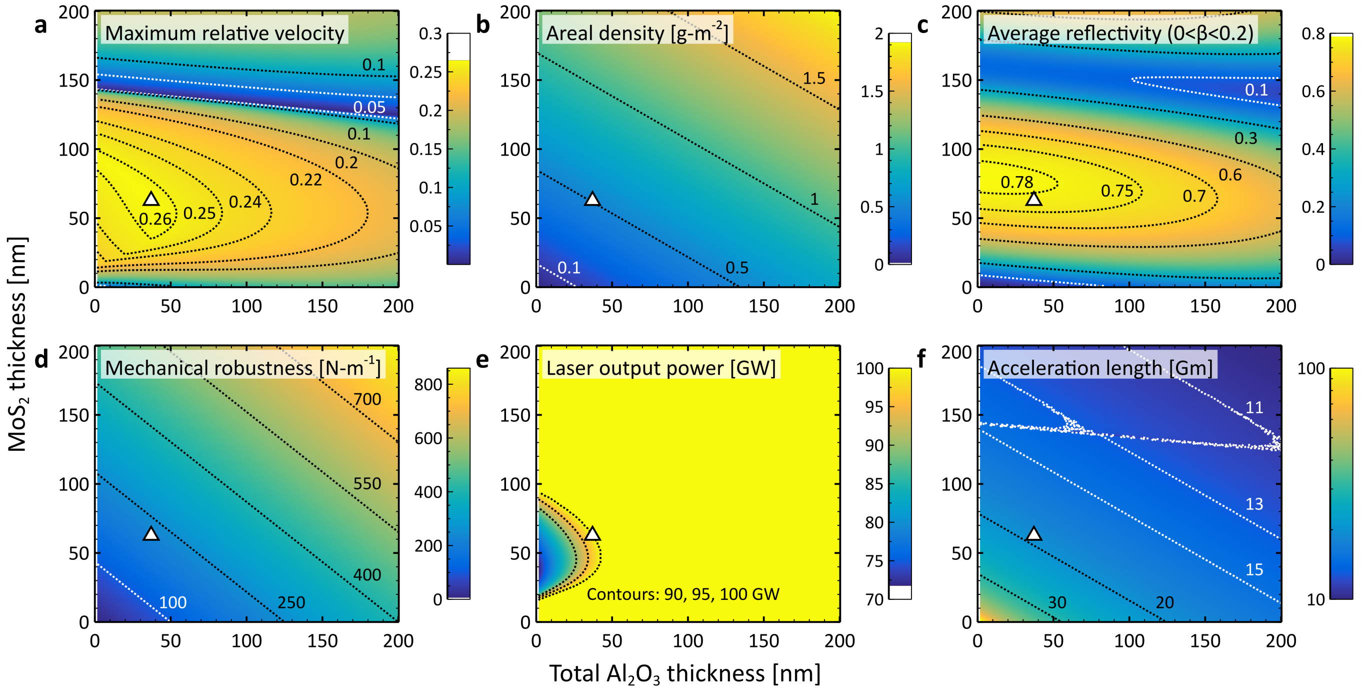

Supplementary Figure 26. | Contour plots showing thickness optimization process for the proposed optimized sail design. Film is designed with the indented trench corrugation using parameters $d_h = 70$ µm, $w_t = 2$ µm, and $h_t = 3$ µm. Note that the abscissa is given in terms of the total Al_2O_3 thickness, *i.e.*, $t_{A,b} + t_{A,t}$, and that we have constrained the optimization to having equal top and bottom Al_2O_3 thickness, *i.e.*, $t_{A,b} = t_{A,t}$. (**a**) Maximum achievable relative velocity (β_{max}). (**b**) Corrugated areal density ($\rho_{a,c,t}$). (**c**) laser band average ($\beta = 0$ to $\beta = 0.2$) reflectivity $\overline{\varrho_{\perp}}$. Note that, for designs achieving $\beta > 0.2$, reflectivity values corresponding to $\beta > 0.2$ are not included in this average. (**d**) Membrane mechanical robustness $\wp$. (**e**) Maximum sustainable constant laser output power Φ_l. (**f**) Acceleration length L. In all panels, the proposed optimized design, featuring Al_2O_3 thickness $t_{A,b} = t_{A,t} \approx 19$ nm and MoS_2 thickness $t_M \approx 63$ nm), is shown with the white triangle.

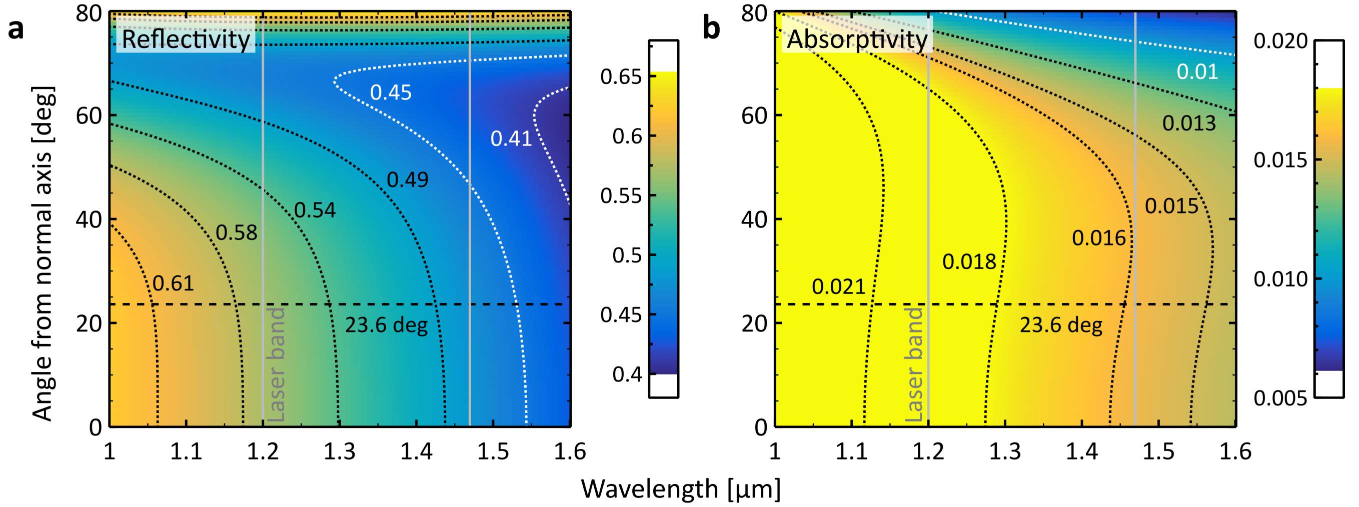

Supplementary Figure 27. | Angular dependence of reflectivity and absorptivity for the fabricated prototype film. Contour plots provide simulations of the (**a**) reflectivity and (**b**) absorptivity for the fabricated prototype film, with thicknesses $t_{A,b} \approx 21$ nm (bottom Al_2O_3 thickness), $t_M \approx 53$ nm (MoS_2), and $t_{A,t} \approx 51$ nm (top Al_2O_3). The simulations do not account for the film's hexagonal corrugation. The vertical gray lines denote the Doppler-shifted wavelength range corresponding to a final relative velocity of $\beta_f = 0.2$: $\lambda = \langle 1.2, 1.4697 \rangle$ µm. The horizontal dashed line corresponds to the maximum angular range accepted by the $NA = 0.4$ numerical aperture in the laser experiments ($0 \leq \theta \leq 23.6°$; see Supplementary Note 9). Simulations shown use measured indices of refraction for Al_2O_3 and MoS_2 (see Supplementary Note 8).

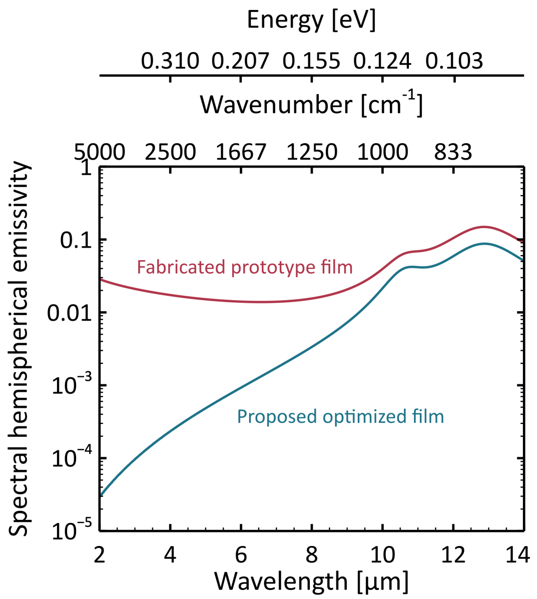

Supplementary Figure 28. | Calculations of the spectral hemispherical emissivity of the fabricated prototype and proposed optimized films. See Equation 82.

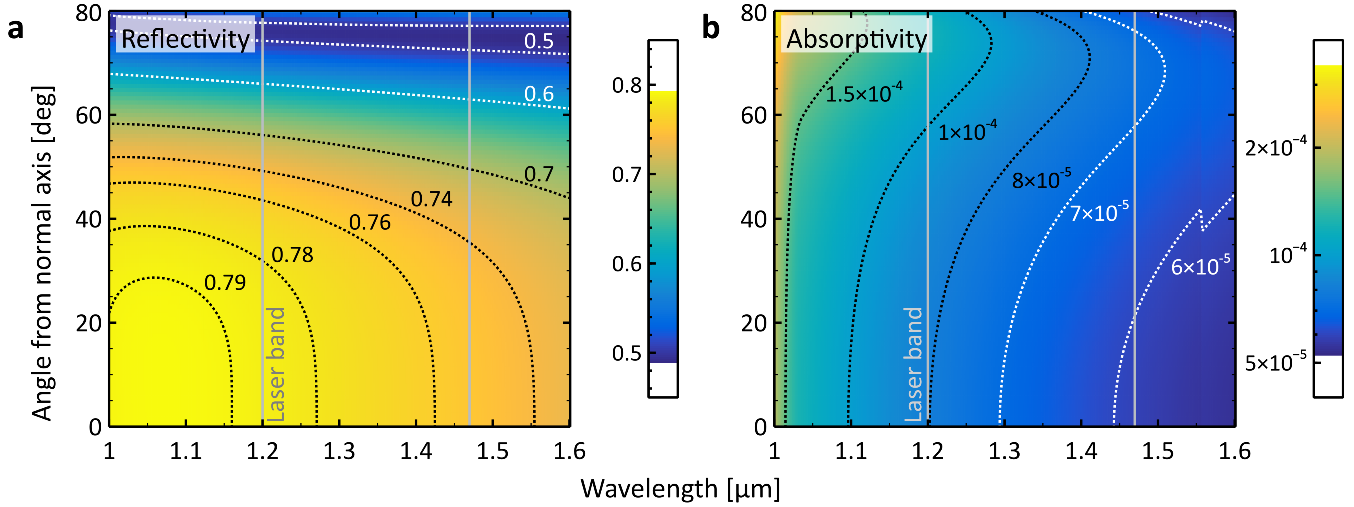

Supplementary Figure 29. | Angular dependence of reflectivity and absorptivity for the proposed optimized film. Contour plots provide simulations of the (**a**) reflectivity and (**b**) absorptivity for the proposed optimized film, with thicknesses $t_{A,b} = t_{A,t} \approx 19$ nm and $t_M \approx 63$ nm. The simulations do not account for the film's hexagonal corrugation. The vertical gray lines denote the Doppler-shifted wavelength range corresponding to a final relative velocity of $\beta_f = 0.2$: $\lambda = \langle 1.2, 1.4697 \rangle$ μm. Simulations use literature index of refraction data [4, 7]. The discontinuity near $\lambda = 1.55$ is related to extrapolating the Kischkat *et al.* [7] data. See also Supplementary Figure 27.

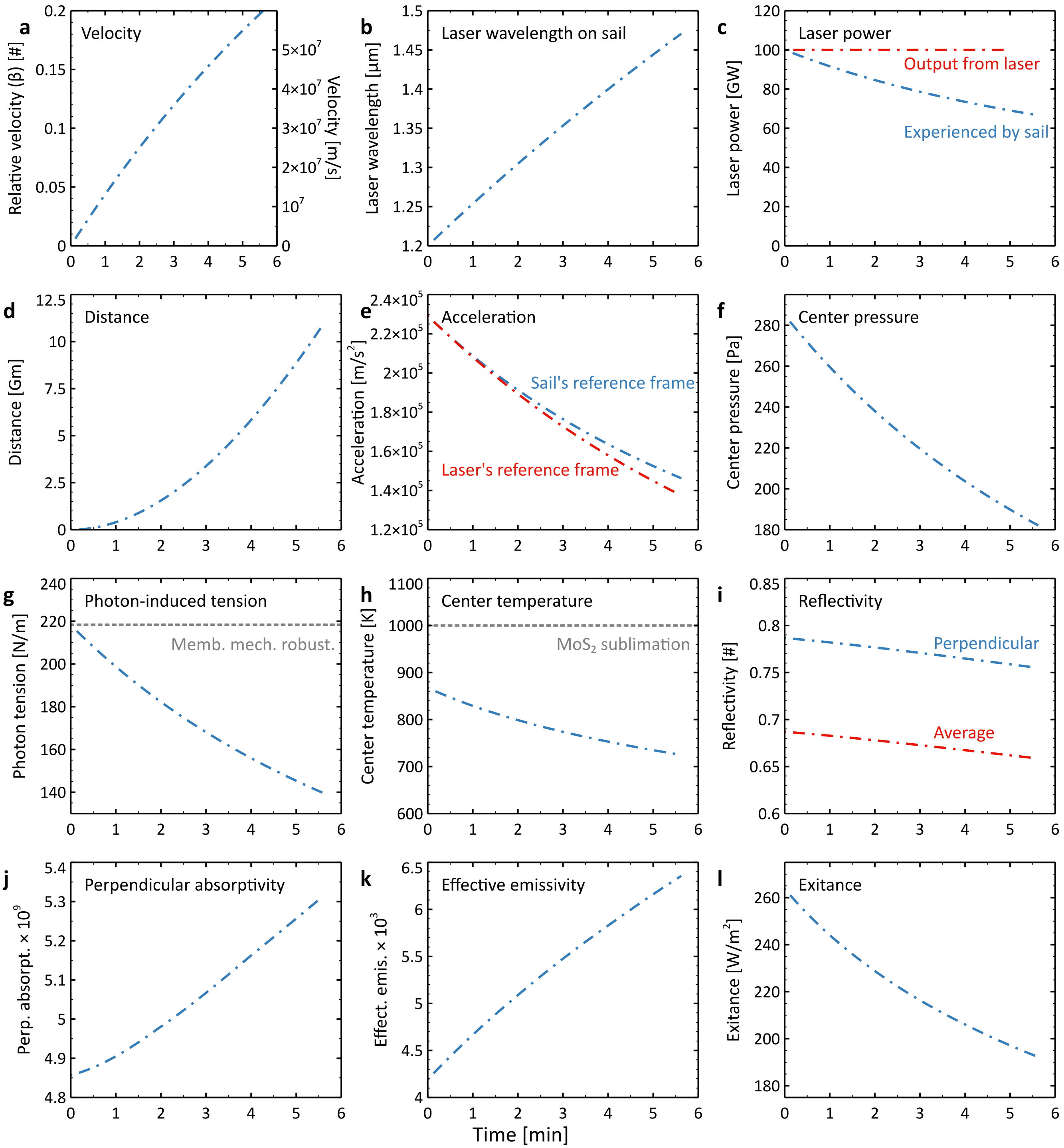

Supplementary Figure 30. | Calculations for the acceleration of our proposed optimized sail design. The simulation is for a spherically-curved circular sail configuration with total sailcraft mass $m_{tot} = 2$ g, sail mass $m_s = 1$ g, equal spherical radius of curvature and diameter ($d_s = s_s$), and optimal optical coefficients for minimal absorptivity. Panels provide show the (**a**) the sailcraft's relative velocity β and laser reference frame velocity v, (**b**) Doppler-shifted laser wavelength λ_s, (**c**) laser output power Φ_l (constant) and power incident on the sail $\Phi_{s,\beta}$, (**d**) distance of the sailcraft from Earth D, (**e**) acceleration in the laser's reference frame $a_{s,l}$ and in the sail's reference frame $a_{s,s}$, (**f**) center-of-sail photon pressure P, (**g**) photon-induced tension Υ, (**h**) center-of-sail temperature T, (**i**) average reflectivity $\varrho_{\beta,a}$ and perpendicular reflectivity $\varrho_{\beta,\perp}$, (**j**) perpendicular (normal) absorptivity $\alpha_{\beta,\perp}$, (**k**) effective emissivity ε_e, and (**l**) exitance $\mathcal{E}_s$. The calculation yields an acceleration distance of $L \approx 11$ Gm, a maximum sail temperature of $T_s \approx 868$ K, an acceleration time of $t_a = 5.6$ min, and a laser-on time of $t_l = 5.0$ min. The proposed optimized film consists of a Al_2O_3-MoS_2-Al_2O_3 composite ($t_{A,b} \approx 19$ nm (bottom Al_2O_3 thickness), $t_M \approx 63$ nm (MoS_2), $t_{A,t} \approx 19$ nm (top Al_2O_3)) with a hexagonally corrugated indented trench structure ($d_h = 70$ µm, $w_t = 2$ µm, $h_t = 3$ µm; see Supplementary Figure 12).

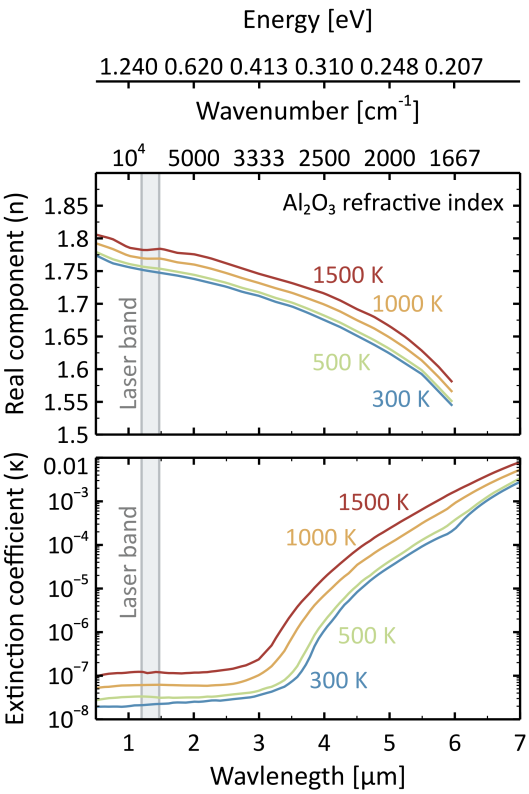

Supplementary Figure 31. | Index of refraction of sapphire (Al_2O_3) as a function of temperature. Data are obtained from Lingart, Petrov, and Tikhonova [5].

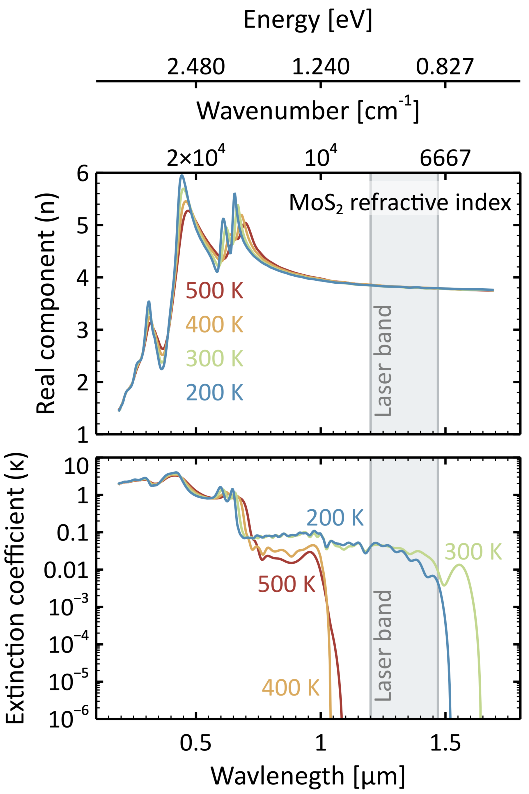

Supplementary Figure 32. | Index of refraction of MoS_2 as a function of temperature. Data are obtained from Liu *et al.* [10].

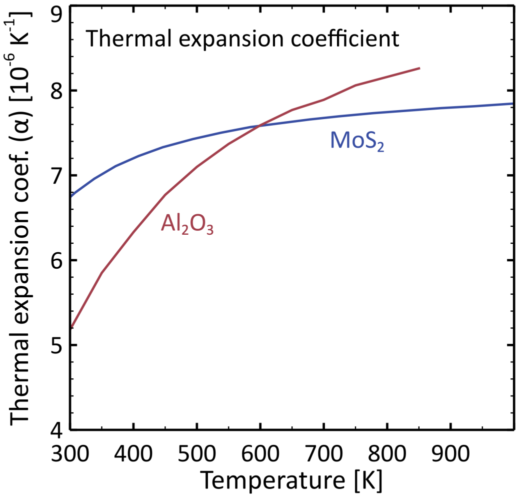

Supplementary Figure 33. | Thermal expansion coefficients of Al_2O_3 and MoS_2 as a function of temperature. Data are obtained from Hayashi, Watanabe, and Inaba [11] (Al_2O_3) and Huang, Gong, and Zeng [12] (MoS_2).

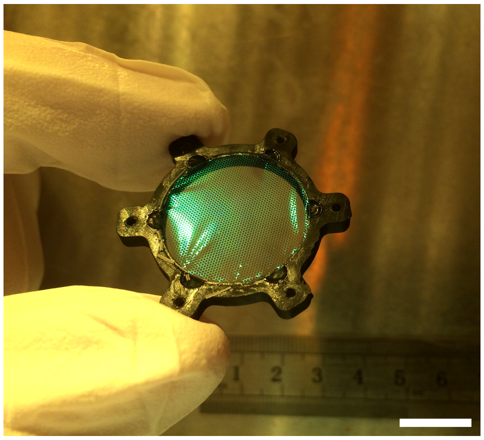

Supplementary Figure 34. | Demonstration of front-side through-hole etching for releasing large-area structures. Hexagonally corrugated Al_2O_3 film with holes that allowed for front-side XeF_2 etching of the Si substrate, attached to a circular 3D-printed frame. Film diameter is 21 mm. The wrinkles originate from imperfect attachment to the circular frame. Scale bar: 10 mm.

Supplementary Tables

Supplementary Table 1. | Select properties for the materials examined in this study. The yield stress values are specific to tensile loading. The properties of MoS_2 listed here are for its thin-film polycrystalline form, which is applicable to our fabricated prototype films. For our optimized design, we assumed that a tensile yield stress of 10% of the monolayer crystalline value will be achievable using future fabrication advances, *i.e.*, $\sigma_y = 2.3$ GPa [13].

Material	Density ρ $\mathrm{kg{\cdot}m^{-3}}$	Yield stress σ_y GPa	Maximum temperature T_{max} K	Young's modulus E GPa	Poisson's ratio ν
Al_2O_3	3200 [14, 15]	2 [16, 17]	2320 [18]	170 [15, 19, 20]	0.24 [16, 20]
MoS_2	5060 [21]	0.75 [21, 22]	1000 [23]	15 [21]	0.25 [21, 24–26]
PDMS	980 [27]	0.005 [28]	620 [29]	-	-
Si	2330 [30]	2 [31–34]	1470 [35]	-	-
Si_3N_4	3200 [36]	14 [37]	1670 [38]	-	-
SiO_2	2030 [39]	1.5 [37, 40]	1450 [41]	-	-
TiO_2	3700 [42]	1.1 [43, 44]	670 [45]	-	-

Supplementary Table 2. | Tabulated data of Figure 4 in the main article. Data included are information on the laser band average ($\beta = 0$ to $\beta = 0.2$) reflectivity $\overline{\varrho_\perp}$, laser band average absorptivity $\overline{\alpha_\perp}$, infrared effective emissivity ε_e (at $T = 1000$ K, $\lambda = 2 - 14$ µm), emissivity-to-absorptivity ratio, thermally-limited power $\Phi_{s,max}$, maximum relative velocity β_{max}, maximum power used in the β_{max} simulation, membrane mechanical robustness $\wp$, areal density ρ_a, laser output wavelength λ_l, perpendicular-to-laser sail area $A_\perp$, and maximum acceleration length to which the laser light can be perfectly focused on the sail L_{max}. Experimentally-measured values are noted with an asterisk symbol ($*$). Note that the units of the thermally limited power $\Phi_{s,max}$ are [MW] whereas those of the power used for the β_{max} simulations are [GW]. Text colors in *citation* column correspond to colors in Figure 4 in the main article.

Citation	Description	$\overline{\varrho_\perp}$	$\overline{\alpha_\perp}$	ε_e	$\frac{\varepsilon_e}{\overline{\alpha_\perp}}$	$\Phi_{s,max}$ (thermal limit) [MW]	β_{max}	Φ_l for β_{max} [GW]	$\wp$ [$\mathrm{N \cdot m^{-1}}$]	ρ_a [$\mathrm{g \cdot m^{-2}}$]	λ_l [µm]	$A_\perp$ [$\mathrm{m^2}$]	L_{max} [Gm]
Ilic, Went, and Atwater (2018) [46]	SiO_2 layers with gaps	0.79	0.0053	0.047	8.9	2.8	0.22	100	1077	1.46	1.2	0.64	11.3
Salary and Mosallaei (2020) [47]	Si disks on Si-SiO_2 bilayer	0.76	0.00038	0.0030	8.0	6.9	0.25	100	268	0.54	1.3	1.74	17.2
Brewer *et al.* (2022) [48]	Si_3N_4-MoS_2-Si_3N_4; holes	0.81	0.000041	0.00063	15	12	0.18	18	21	0.13	1.2	7.2	37.9
Lien *et al.* (2022) [49]	Si_3N_4; holes	0.44$*$	0.000077	0.063	823	348	0.17	100	5892	1.94	1.064	0.48	11.0
Santi *et al.* (2022) [50]	TiO_2-SiO_2-TiO_2	0.78	0.0020	0.032	16	0.26	0.23	100	571	1.31	1.064	0.71	13.4
Taghavi and Mosallaei (2022) [51]	Si disks on Si-SiO_2 bilayer	0.76	0.00038	0.0047	12	4.9	0.19	79	296	1.18	1.3	0.79	11.6
Chang *et al.* (2024) [52]	Si_3N_4 with holes on Si	0.66$*$	0.076$*$	0.017	0.23	0.074	0.19	100	2353	1.54	1.3	0.60	10.1
Norder *et al.* (2025) [53]	Si_3N_4 with holes	0.38$*$	0.000046	0.057	1259	1369	0.18	100	1260	0.45	1.55	2.05	15.6
Whittam *et al.* (2025) [54]	Si/SiO_2 nanospheres in PDMS	0.89	0.00022	0.025	113	2.6	0.026	0.51	1.63	0.69	1.0	1.34	19.6
Fabricated prototype film (this study)	Corrugated Al_2O_3-MoS_2-Al_2O_3	0.51$*$	0.029$*$	0.023	0.78	0.12	0.21	100	184	0.68	1.2	1.37	16.5
Proposed optimized film (this study)	Corrugated Al_2O_3-MoS_2-Al_2O_3	0.77	0.00014	0.0038	27	5.6	0.26	100	218	0.51	1.2	1.84	19.1

Supplementary Note 1: Additional information on film fabrication

Here we present supplementary details concerning our film fabrication process. Supplementary Figure 1 summarizes the fabrication steps for the protruding ribs film configuration (see also Figure 3(a) in the main article). Supplementary Figure 2 shows photographs of films on Si chips. Supplementary Figure 3 provides more images of the fully suspended prototype (indented trenches configuration) shown in Figure 1(b) in the main article. Supplementary Figure 4 provides micrographs of the corrugated film shown in Supplementary Figure 2(b). The focal point for these images was the tops of the hexagons rather than the bottoms of the trenches. Supplementary Figure 5 shows a silicon chip with three 500 µm laser-drilled holes in its back side. Finally, Supplementary Figure 6 shows the fixture that we used during XeF_2 etching to promote equal gas pressure on both sides of the chips, protecting the films against excessive force when venting the etching chamber.

Alumina and molybdenum disulphide are remarkably compatible for thin-film growth. Numerous studies have grown MoS_2 on Al_2O_3 [55–57] and likewise have grown Al_2O_3 on MoS_2 [58–66], reporting good adhesion between the two films. We speculate that Mo−O bonds form between the base Al_2O_3 film and the MoS_2 film during the sputtering and sulfurization processes, promoting adhesion. During the second Al_2O_3 deposition to form the upper alumina layer, the trimethylaluminum (TMA) precursor may react with undercoordinated Mo or S and thus promote adhesion.

Supplementary Note 2: Additional information on film characterization

Supplementary Figure 7 presents several cross-sectional transmission electron microscopy (TEM) images of sample MoS_2 films. For these images, we prepared samples using the Xe plasma focused ion beam approach [67] and subsequently transferred them to half-grids using the *in situ* liftoff technique [68]. The orientation of the grains is determined by various factors that include strain, transformation rate, and defects present during the sulfurization process [69–71].

In Supplementary Figure 7(e) a native oxide (SiO_2) is visible. In our experience, this oxide is removed during the gaseous XeF_2 etching process by trace HF formed as the XeF_2 reacts with residual water vapor in the chamber: $2\,XeF_2 + 2\,H_2O \longrightarrow 2\,Xe + O_2 + 4\,HF$. It may also be removed through other processes [72, 73]. We later experimented with removing this native oxide prior to the first atomic layer deposition step of Al_2O_3 by dipping the corrugated Si wafer mold in 49% HF for about 1 min ($SiO_2 + 6\,HF \longrightarrow H_2SiF_6 + 2\,H_2O$) and then quickly moving it to the deposition tool to avoid re-forming the native oxide (transfer time typically <3 min). This is a reliable way to ensure that no SiO_2 remains on the films after they are suspended.

Supplementary Figure 8 presents atomic force microscopy (AFM) scans of 45 nm-thick and 75 nm-thick MoS_2 samples on SiO_2/Si substrates.

Supplementary Figure 9 provides the spectrum obtained in the energy dispersive X-ray spectroscopy (EDS) imaging corresponding to Figure 3(b) in the main article. The elemental C peak is likely due to hydrocarbons adsorbed to the sample surface and is common to most EDS spectra. Supplementary Figures 10 and 11 are similar to Figure 3(b) in the main article, except they provide normal and 45° perspective views of several unit cells, respectively.

Supplementary Note 3: Mechanics of corrugated films

Here we derive the areal density of a composite hexagonally-corrugated film and explain how we calculate the enhanced bending stiffness and reduced tensile stiffness that hexagonal corrugation brings to films. Supplementary Figures 12 and 13 show the film dimensions relevant to this work in the indented trenches and protruding ribs configurations, respectively. The important dimensions are the hexagon diameter d_h, trench width w_t (or rib width w_r), trench height h_t (or rib height h_r), and film thicknesses $t_{A,b}$ (bottom), t_M (middle), and $t_{A,t}$ (top). For simplicity, we define and measure d_h, w_t (and w_r), and h_t (and h_r) based on the Si mold's dimensions. Also, the side length of the hexagons a_h can be calculated by

$$a_h = \frac{d_h}{\sqrt{3}} \tag{1}$$

We calculate the areal density for non-corrugated (flat, planar) films $\rho_{a,p}$ using

$$\rho_{a,p} = \rho_A \left(t_{A,b} + t_{A,t}\right) + \rho_M t_M \tag{2}$$

Here, $\rho_A = 3200\ \mathrm{kg \cdot m^{-3}}$ is the density of Al_2O_3 [15] and $\rho_M = 5060\ \mathrm{kg \cdot m^{-3}}$ is the density of MoS_2 [21] (see Supplementary Table 1). Calculating the areal density for corrugated films, $\rho_{a,c,t}$ or $\rho_{a,c,r}$, is more involved. We obtain these values by finding the ratio of the mass of a unit cell M_{uc} to its planar area A_{uc}. For the indented trench configuration this is written

$$\rho_{a,c,t} = \frac{M_{uc,t}}{A_{uc,t}} \tag{3}$$

and for the protruding ribs films this is written

$$\rho_{a,c,r} = \frac{M_{uc,r}}{A_{uc,r}}. \tag{4}$$

In the case of indented trenches, the unit cell mass is given by

$$
\begin{aligned}
M_{uc} = \frac{\sqrt{3}}{2}\rho_A \Bigg(& t_{A,b}\left(d_h + w_t\right)^2 \\
& + h_t\left(\left(d_h + 2t_{A,b}\right)^2 - d_h^2\right)\Bigg) \\
+ \frac{\sqrt{3}}{2}\rho_M \Bigg(& t_M\left(d_h + w_t\right)^2 \\
& + h_t\left(\left(d_h + 2\left(t_{A,b} + t_M\right)\right)^2 - \left(d_h + 2t_{A,b}\right)^2\right)\Bigg) \\
+ \frac{\sqrt{3}}{2}\rho_A \Bigg(& t_{A,t}\left(d_h + w_t\right)^2 \\
& + h_t\Big(\left(d_h + 2\left(t_{A,b} + t_M + t_{A,t}\right)\right)^2 \\
& \quad - \left(d_h + 2\left(t_{A,b} + t_M\right)\right)^2\Big)\Bigg)
\end{aligned} \tag{5}
$$

and in the case of protruding ribs, the unit cell mass is

$$
\begin{aligned}
M_{uc} = \frac{\sqrt{3}}{2}\rho_A \Bigg(& t_{A,b}\left(d_h + w_r\right)^2 \\
& + h_r\left(d_h^2 - \left(d_h - 2t_{A,b}\right)^2\right)\Bigg) \\
+ \frac{\sqrt{3}}{2}\rho_M \Bigg(& t_M\left(d_h + w_r\right)^2 \\
& + h_r\left(\left(d_h - 2t_{A,b}\right)^2 - \left(d_h - 2\left(t_{A,b} + t_M\right)\right)^2\right)\Bigg) \\
+ \frac{\sqrt{3}}{2}\rho_A \Bigg(& t_{A,t}\left(d_h + w_r\right)^2 \\
& + h_r\Big(\left(d_h - 2\left(t_{A,b} + t_M\right)\right)^2 \\
& \quad - \left(d_h - 2\left(t_{A,b} + t_M + t_{A,t}\right)\right)^2\Big)\Bigg)
\end{aligned} \tag{6}
$$

In Equations 5 and 6, the first term in each parenthetical group (multiplied by the film thickness) computes the mass associated with the hexagon bases and bottoms of the trenches (or tops of the ribs), and the second term (multiplied by the rib or trench height) determines the mass of the vertical sections of the trenches (ribs). The area of the unit cell is given by

$$A_{uc,t} = \frac{\sqrt{3}}{2}\left(d_h + w_t\right)^2 \tag{7}$$

and

$$A_{uc,r} = \frac{\sqrt{3}}{2}\left(d_h + w_r\right)^2 \tag{8}$$

for the trench and rib configurations, respectively. We will henceforth write the equations in terms of the indented trench configuration using w_t and h_t, but these two variables can be substituted for w_r and h_r, respectively, for the protruding ribs configuration in the remaining expressions.

The indented trench or protruding rib areas increase the bending stiffness of the film relative to its planar (non-corrugated) counterpart. Davami *et al.* [74] quantified this performance using the bending stiffness enhancement factor $\mathbb{B}$, defined here as the ratio of the bending stiffness for a corrugated film K_c to that of a planar film, K_p. Thus defined, corrugated films with $\mathbb{B} > 1$ are more stiff in bending than their planar versions.

$$\mathbb{B} = \frac{K_c}{K_p} \tag{9}$$

The original derivation provided by Davami *et al.* [74] concerned a single-layer film, whereas our films are three-layer composites. A rigorous treatment of the bending properties would ideally account for the impact of the three layers independently [75, 76]. However, the purpose of this analysis is to illustrate the general improvement that hexagonal corrugation provides to light sail films, rather than to obtain the numerically exact performance enhancement. Therefore, instead of re-deriving the Davami *et al.* equations for composite films, we instead for expediency calculate here the equivalent single-layer film properties for our three-layer films, and then proceed to use the Davami *et al.* expressions as-is with these single-layer metrics. The required effective unified film properties are the thickness, Young's modulus, and Poisson's ratio, and we obtain these using standard mixing rules [77–79].

The unified thickness t_u of the composite film is sum of the thicknesses of the individual layers.

$$t_u = t_{A,b} + t_M + t_{A,t} \tag{10}$$

We calculate the unified Young's modulus E_u using a slight modification of the traditional Voigt equation that accounts for the Poisson effect [77].

$$
\begin{aligned}
E_u = & \left(f_A E_A + f_M E_M\right) \\
& + \frac{f_A f_M E_A E_M \left(\nu_A - \nu_M\right)^2}{f_A E_A \left(1 - \nu_M^2\right) + f_M E_M \left(1 - \nu_A^2\right)}
\end{aligned} \tag{11}
$$

Here, $\nu_A = 0.24$ and $\nu_M = 0.25$ are the Poisson's ratios for Al_2O_3 [16, 20] and MoS_2 [21, 24–26], respectively, and $E_A = 170$ GPa [15, 19, 20] and $E_M = 15$ GPa [21] are the corresponding Young's moduli (see Supplementary Table 1). The terms f_A and f_M are the fractional thicknesses of the Al_2O_3 and MoS_2 materials, respectively, defined as

$$f_A = \frac{t_{A,b} + t_{A,t}}{t_u} \tag{12}$$

and

$$f_M = \frac{t_M}{t_u}. \tag{13}$$

Similarly, we calculate the unified Poisson's ratio ν_u value using [79]

$$\nu_u = f_A \nu_A + f_M \nu_M. \tag{14}$$

Returning to Equation 9, the K_p term corresponds to the bending stiffness of a planar (non-corrugated) plate with the same thickness, Young's modulus, and Poisson's ratio as the composite, namely t_u, E_u, and ν_u, respectively. This can be calculated by

$$K_p = \frac{E_u t_u^3}{12\left(1 - \nu_u^2\right)} \tag{15}$$

The effective corrugated bending stiffness can be obtained by taking into account the relative area and stiffness of the trench (rib) intersections and the main region of the hexagonal unit cell (see Davami *et al.* [74]), according to

$$K_c = \frac{A_{uc,t}}{\frac{A_m}{K_m} + \frac{A_i}{K_p}}. \tag{16}$$

In this expression, the area of the unit cell $A_{uc,t}$ is given in Equation 7, A_i is the area of the rib intersections, given by

$$A_i = \frac{\sqrt{3}}{2} w_t^2, \tag{17}$$

and A_m is the area of the main region of the unit cell, obtained by subtraction:

$$A_m = A_{uc,t} - A_i. \tag{18}$$

The parameter K_p in Equation 16 corresponds to the stiffness of the intersections of the trenches (ribs). In this treatment, we consider the vertical hexagon walls to be associated with the areas of the bases of the hexagons, rather than these trench/rib intersections. This is strictly true for the rib-based design (Supplementary Figure 13) and approximately correct for the trench design (Supplementary Figure 12). Thus, since we do not consider the intersection areas to contain any vertical components, the associated stiffness is identical to that of a flat plate (Equation 15).

The parameter K_m in Equation 16 corresponds to the stiffness of the main area of the unit cell, which we associate with the vertical trench (rib) walls. These greatly enhance the stiffness of the main area, as can be seen by comparing Equation 15 to Equation 19, below.

$$K_m = \frac{E_u}{12\left(1-\nu_u^2\right)} \left(t_u^3 + 3 t_u h_t^2 + \frac{2 t_u h_t^3}{a_h + w_t} \right). \tag{19}$$

Jiao *et al.* [80] later demonstrated that hexagonal corrugation reduces the tensile stiffness, or increases the "stretchiness," of films relative to their non-corrugated counterparts. In this work, we define the ratio of the tensile stiffness of a corrugated plate S_c to that of a planar (non-corrugated) plate S_p as the tensile stiffness reduction factor, $\mathbb{T}$. Specifically, corrugated films with $\mathbb{T} < 1$ have lower tensile stiffness, or are more stretchy, than their planar relatives. As with $\mathbb{B}$ (Equation 9), we employ the unified thickness and mechanical properties derived *via* mixing rules from our composite sail film in the following equations.

$$\mathbb{T} = \frac{S_c}{S_p} \tag{20}$$

According to the formulation of Jiao *et al.* [80], the tensile stiffness of a planar plate can be calculated by

$$S_p = \frac{E_u t_u}{1-\nu_u^2}, \tag{21}$$

where E_u is obtained in Equation 11. The tensile stiffness of a hexagonally-corrugated plate can be obtained through

$$S_c = \frac{E_e h_t}{1-\nu_u^2}, \tag{22}$$

where E_e is the effective Young's modulus for the corrugated film. We obtain the effective Young's modulus according to

$$E_e = \frac{E_u}{1-\nu_e^2} \left(\alpha_c \frac{I_c}{t_u d_h^3} + \beta_c \frac{h_t^4 w_t^4}{t_u^2 d_h^5 \left(2h_t + w_t\right)} \right) \tag{23}$$

wherein I_c is the moment of inertia of the U-shaped trenches (ribs), ν_e is the effective Poisson's ratio of the corrugated film, and $\alpha_c = 0.234$ and $\beta_c = 0.062$ are empirical parameters derived from numerical plate stretching simulations [80].

$$I_c = \frac{1}{12} t_u w_t^3 + \frac{1}{6} h_t t_u^3 + \frac{1}{2} h_t t_u w_t^2 \tag{24}$$

$$\nu_e = \frac{\Lambda_c - 36 I_c}{\Lambda_c + 108 I_c} \tag{25}$$

In Equation 25, Λ_c is given by

$$\Lambda_c = t_u \left(2h_t + w_t\right) \left(d_h^2 + 3\left(2.4 + 1.5\nu_u\right) w_t^2\right). \tag{26}$$

We used these equations to predict $\rho_{a,c,t}$, $\mathbb{B}$, and $\mathbb{T}$ as a function of $\frac{d_h}{w_t}$ and $\frac{h_t}{t_u}$ for hexagonally-corrugated Al_2O_3-MoS_2-Al_2O_3 films. The results for $\mathbb{B}$ are shown in Figure 3(e) in the main article, and those of $\rho_{a,c,t}$ and $\mathbb{T}$ are shown in Supplementary Figures 14 and 15 for design families corresponding to our fabricated prototype film and for our proposed optimized film, respectively (separate plots are required to display $\rho_{a,c,t}$ and $\mathbb{T}$ for the two films because of their different compositions and thicknesses; however, in the case of $\mathbb{B}$ in Figure 3(e) of the main article, we could combine the plots for the fabricated prototype and proposed optimized films since the Young's modulus cancels out in Equation 9 and the Poisson ratios of Al_2O_3 and MoS_2 are very similar).

Examining Figure 3(e) in the main article, we may observe that, for sufficiently high $\frac{h_t}{t_u}$, $\mathbb{B}$ can be increased by increasing $\frac{d_h}{w_t}$. Likewise, for high $\frac{d_h}{w_t}$, $\mathbb{B}$ can be increased by increasing $\frac{h_t}{t_u}$. For hexagonal patterns with $\frac{h_t}{t_u} \approx \frac{d_h}{w_t}$, $\mathbb{B}$ can be increased by increasing both ratios. Our proposed optimized design features $\mathbb{B} \approx 900$, which we achieved by increasing $\frac{d_h}{w_t}$ relative to our fabricated prototype film. Simultaneously, our proposed optimized design features smaller trench heights h_t, which, due to the shape of the $\mathbb{B}$ contours, maintains a high bending stiffness while decreasing the film's areal density (see Supplementary Figure 15).

Viewing Supplementary Figures 14(a) and 15(a), we observe that "stretchier" designs (lower $\mathbb{T}$), which are less likely to tear under high strain, feature high $\frac{d_h}{w_t}$ and moderate $\frac{h_t}{t_u}$. Such designs also correspond to lower areal density values (Supplementary Figures 14(b) and 15(b)), which generally lead to faster light sail acceleration. When $\frac{h_t}{t_u}$ ratios become very low, however, $\mathbb{B}$ values decline (Figure 3(e) in the main article), making the sail more less robust. Our proposed optimized sail corrugation pattern navigates this trade-off.

It can be useful to estimate the equivalent film thickness and material Young's modulus required to achieve the bending and tensile stiffness values in a non-corrugated (flat) plate that are equivalent to those of a corrugated plate. We refer to these as t_{eq} and E_{eq}, and

obtain them using the corrugated plate bending and tensile stiffness values, K_c and S_c, respectively, in Equations 15 and 21, respectively.

$$t_{eq} = \sqrt{\frac{12K_c}{S_c}} \tag{27}$$

$$E_{eq} = \sqrt{\frac{S_c^3 \left(1 - \nu^2\right)^2}{12K_c}} \tag{28}$$

In Equation 28, the Poisson's ratio ν refers to that of the equivalent flat film, which we assume to be similar to that of the corrugated film ν_e. For both our fabricated prototype and proposed optimized hexagonal pattern designs, $t_{eq} > t_u$ and $E_{eq} < E_u$, reflecting the benefits of corrugation in terms of mass savings and material selection. In other words, a non-corrugated (flat) film with the same bending stiffness and tensile "stretchiness" as our corrugated films would need to be thicker and be made from materials with lower Young's moduli.

Finally, to avoid the possibility of folds or creases in hexagonally corrugated films, we mention the so-called "no-straight-line rule," [81]. This guideline states that kinks are less likely when it is impossible to draw a straight line through the pattern without contacting vertical sidewalls. The mathematical interpretation of this statement is that $d_h \geq 3w_t$. Notice that the film in Figure 1(c) features $d_h \approx 36$ µm and $w_t \approx 15$ µm, which allowed us to bend the film to show its corrugation (*i.e.*, $d_h < 3w_t$, breaking the rule).

Supplementary Note 4: Explanation of mechanical properties

Here we explain the values of the mechanical properties that used in our calculations. Whenever possible, we obtained values directly from published works. However, in some cases where data were not available, we derived or inferred these values.

All density values shown in Supplementary Table 1 are for microfabricated thin films, with the exception of polydimethylsiloxane (PDMS), for which we report the bulk density, and Si, for which we report the crystalline density.

We obtained the tensile yield stress for Al_2O_3 using

$$\sigma_y = E\epsilon_y, \tag{29}$$

where ϵ_y is the tensile yield strain. Jen, Bertrand, and George reported critical tensile strains for atomic layer deposition-based Al_2O_3 films ranging from 0.0052 to 0.024 for thicknesses ranging from 80 nm to 5 nm (respectively) [17]. Selecting an intermediate yield strain, together with a Young's modulus of $E = 170$ GPa, obtained for thin atomic layer deposition-based films [15, 19, 20], provided us with a conservative tensile yield stress of $\sigma_y = 2$ GPa. We used the melting point of alumina as its thermal limit, $T_{max} = 2320$ K, because of its high vacuum heating stability [18].

We also derived the tensile yield stress for MoS_2 using Equation 29, with $E = 15$ GPa [21] and $\epsilon_y = 0.05$ [22]. These values are taken for polycrystalline MoS_2 fabricated using a process similar to that which we used. However, as discussed in Sections 13 and 14.12, for the proposed optimized design, we assumed that future fabrication advances would allow MoS_2 to be deposited in thick crystalline layers that would absorb minimally in the laser band. For the associated acceleration calculations, we conservatively assumed that these thicker layers could achieve 10% of the perfect monolayer crystalline tensile yield strength [13], giving a value of $\sigma_y = 2.3$ GPa. Recent measurements of the thickness-dependence of the Young's modulus of $MoSe_2$, which is similar to MoS_2, suggest this is a reasonable assumption [82]. Note that the Young's modulus cancels out of the bending and tensile stiffness enhancement factor ratios (Equations 9 and 20), thus making these calculations agnostic to our selection of polycrystalline or crystalline properties for MoS_2. For the thermal limit of MoS_2 we chose $T_{max} = 1000$ K, which is the point at which it begins to sublimate in a vacuum [23].

For polydimethylsiloxane (PDMS), tensile yield stress values are usually in the range 3.5-5.2 MPa; we selected a round number at the upper end of this range, namely $\sigma_y = 5$ MPa [28]. Thermal breakdown of PDMS occurs over a range of temperatures, often beginning at roughly 620 K, which we used as its thermal limit here [29].

For both crystalline and polycrystalline Si, tensile yield stresses of about 1-3 GPa have been reported [31–34]. We selected $\sigma_y = 2$ GPa as an intermediate value. Although the melting point of Si is 1690 K [83], it begins to sublime at around 1470 K [35], which we thus selected for its thermal limit. Note that recent studies have suggested that, due to its increasing absorption coefficients with temperature, a limiting temperature for Si for laser propulsion applications could be as low as 500 K [84]. However, since similar information is not presently available for the other materials considered in this study, we have adopted limits based on melting/sublimation/decomposition points.

For SiO_2, tensile yield stress values in the range 0.6-1.9 GPa have been reported; we selected $\sigma_y = 1.5$ GPa [37, 40]. SiO_2 melts at 1980 K [85] but begins to chemically break down around 1450 K [41], so we used the latter as our thermal limit.

For Si_3N_4, we used Equation 29 and the results of Yoshioka *et al.* [37] to derive $\sigma_y = 14$ GPa. In terms of the thermal limit, Si_3N_4 melts at about 2170 K [85] but decomposes at 1670 K [38], so we used the latter as its limiting temperature.

Finally, for TiO_2, we were unable to find a tensile yield stress in the literature. However, using Equation 29 with a Young's modulus of 151 GPa [43] and a yield strain of 0.0075 [44], we obtained $\sigma_y = 1.1$ GPa. TiO_2 is known to be unstable when heated under vacuum conditions; although its melting point is 2110 K [86], it begins to decompose around 670 K [45]. We used the latter temperature as its thermal limit.

Supplementary Note 5: Relativistic acceleration of light sails

Here we outline the equations used to determine the acceleration, distance, time, and laser output energy throughout the laser-illumination phase of a relativistic light sail's journey. These equations are largely obtained from the Supporting Information for Campbell *et al.* [87], where a full derivation can be found. They

are similar to those found in Parkin [88], Kulkarni, Lubin, and Zhang [89], Ilic, Went, and Atwater [46], Fűzfa, Dhelonga-Biarufu, and Welcomme [90], and Pegoraro, Livi, and Macchi [91].

5.1. *Acceleration*

Laser photons arriving in the accelerating sail's frame of reference will have wavelengths λ that are Doppler-shifted relative to their initial values, according to

$$\lambda_s = \lambda_l \gamma \left(1 + \beta\right), \tag{30}$$

where

$$\gamma = \frac{1}{\sqrt{1-\beta^2}} \tag{31}$$

is the Lorentz factor,

$$\beta = \frac{v}{c} \tag{32}$$

is the relative velocity, v is the sail velocity in the laser's reference frame, c is the speed of light, and subscripts s and l denote the sail and laser reference frames, respectively.

It is more straightforward to describe the acceleration of the sail in terms of β rather than in terms of time, because the reference frames of the sail and laser must be related through relativity. The acceleration of the sail in the Earth-bound laser array's frame of reference can be written

$$a_{s,l} = \frac{2\varrho_{\beta,a}\Phi_{l,\beta}}{m_{tot}c\gamma^3}\left(\frac{1-\beta}{1+\beta}\right), \tag{33}$$

and in the sail's frame of reference (the proper acceleration) is

$$a_{s,s} = \frac{2\varrho_{\beta,a}\Phi_{l,\beta}}{m_{tot}c}\left(\frac{1-\beta}{1+\beta}\right). \tag{34}$$

In these expressions, $\varrho_{\beta,a}$ is the average reflectivity of the sail film when the sail's relative velocity is β (an average is required if the sail has a curved shape, rather than a flat shape [87], see Supplementary Note 7.2), $\Phi_{l,\beta}$ is the output power of the laser (subscript β indicates that we could choose to allow this output power to change as the sail accelerates), and m_{tot} is the total rest (*i.e.*, proper, invariant, intrinsic) mass of the sailcraft (including sail, payload, and any connecting tethers). We emphasize that, because of the Doppler shift of the wavelength of the incident photons, the reflectivity of the sail changes as it accelerates. Since $\gamma > 1$ for $\beta > 0$, $a_{s,s} > a_{s,l}$, *i.e.*, the sail locally experiences a stronger acceleration than what the laser observes from its fixed vantage point.

5.2. *Power*

The laser power experienced by the sail $\Phi_{s,\beta}$ is related to that outputted by the laser according to

$$\Phi_{s,\beta} = \Phi_{l,\beta}\left(\frac{1-\beta}{1+\beta}\right). \tag{35}$$

Thus, for $\beta > 0$, the power experienced by the sail is less than that outputted by the laser. This decrease is associated with the red-shift of the photons in the sail's frame of reference and with the decrease in photon flux as the sail accelerates away from the incoming laser light. Note that $\Phi_{l,\beta}$ is not the laser output power at the instant in time that the sail's relative velocity is β; rather, it is the power required to have been outputted by the laser at some previous time (see Equation 40) such that those photons will reach the sail when its relative velocity is β. Also, for the sake of clarity, $\Phi_{l,\beta}$ is a constant in the case of constant laser output power.

5.3. *Distance*

As explained in the Supporting Information for Campbell *et al.* [87], the distance traveled by the sail when its relative velocity has reached β can be expressed as [46, 92]

$$D = \frac{m_{tot}c^3}{2}\int_0^\beta \frac{\beta\gamma}{\varrho_{\beta,a}\Phi_{l,\beta}(1-\beta)^2}d\beta. \tag{36}$$

We define the acceleration length L as the distance traveled by the sail while being illuminated by laser-generated photons as it accelerates to a final velocity of $v_f = \frac{1}{5}c$:

$$L = D|_{\beta=\beta_f=0.2} \tag{37}$$

5.4. *Time*

The time t required for the sail to achieve a relative velocity of β, as counted in the laser's frame of reference, is given by

$$t = \frac{m_{tot}c^2}{2}\int_0^\beta \frac{\gamma}{\varrho_{\beta,a}\Phi_{l,\beta}(1-\beta)^2}d\beta. \tag{38}$$

Thus, we define the acceleration time of the sail as

$$t_a = t|_{\beta=\beta_f=0.2}. \tag{39}$$

However, photons require a finite amount of time to travel from the laser to the accelerating sail. Thus, in order for a photon to reach the sail at time t, it must be released at an earlier time [88, 89, 93]

$$t_p = t - \frac{D}{c}. \tag{40}$$

Notice that $t_p < t$ for $\beta > 0$. The photon release time can be expressed in terms of β in the form

$$t_p = \frac{m_{tot}c^2}{2}\int_0^\beta \frac{\gamma}{\varrho_{\beta,a}\Phi_{l,\beta}(1-\beta)}d\beta, \tag{41}$$

and the total time duration for which the laser array must emit photons t_l can be expressed as

$$t_l = t_p|_{\beta=\beta_f=0.2}. \tag{42}$$

5.5. *Laser array size*

It is useful to estimate the required areal size of the Earth-bound laser array. Optimal light sail acceleration occurs when the laser spot diameter $d_{l,s}$ is centered and focused on, and does not exceed, the light sail diameter d_s until the sail has reached its final relative

velocity of $\beta = \beta_f$ [89]. The spot size is limited by diffraction to be approximately

$$d_{l,s} = 2\lambda_l \frac{D}{d_{l,E}}, \tag{43}$$

where $d_{l,E}$ is the laser array diameter on Earth. Thus, for $\beta = \beta_f$ at the optimal condition $d_{l,s} = d_s$,

$$d_{l,E} = 2\lambda_l \frac{L}{d_s}. \tag{44}$$

5.6. *Pressure*

The incident laser light will cause radiation pressure on the sail [94]. The pressure will vary with position on the sail, but for circular curved sails will likely have its maximum at the sail's radial center [87]. This maximum pressure is given by

$$P = \frac{8\varrho_{\beta,\perp}\Phi_{l,\beta}}{\pi c d_s^2}\left(\frac{1-\beta}{1+\beta}\right). \tag{45}$$

Here, $\varrho_{\beta,\perp}$ is the relative-velocity-dependent perpendicular (normal-to-surface) reflectivity of the sail and d_s is the sail's circular diameter. For a flat (uncurved) two-dimensional-isotropic sail perfectly aligned to the incident photons, $\varrho_{\beta,\perp}$ is the same everywhere on the surface. For a curved sail, $\varrho_{\beta,\perp}$ is the normal-to-surface reflectivity at the sail's radial center. We can observe that, if the laser output power and reflectivity are held constant, the pressure experienced by the sail will decrease throughout the acceleration phase (as β increases) due the Doppler shift and photon flux effects mentioned above.

5.7. *Thermal balance*

The light sail will absorb a small fraction of the incident laser photons, and will thus adopt an elevated temperature at which the energy it absorbs equals its radiant exitance. The peak temperature, likely occurring at the point where the sail film is perpendicular to the incident photons (*i.e.*, at the radial center of a curved sail), can be determined implicitly through an energy balance of the form [46–48, 87]

$$\frac{\alpha_{\beta,\perp}\Phi_{l,\beta}}{A_\perp}\left(\frac{1-\beta}{1+\beta}\right) = \int_{\lambda_1}^{\lambda_2} \frac{4\pi h c^2 \varepsilon_\lambda}{\lambda^5\left(\exp\left(\frac{hc}{\lambda k_B T}\right)-1\right)} d\lambda. \tag{46}$$

Here, $\alpha_{\beta,\perp}$ is the perpendicular-to-sail (normal) absorptivity of the sail film when the sail's relative velocity is β, ε_λ is the hemispherical spectral emissivity for the sail film at wavelength λ, h is Planck's constant, k_B is Boltzmann's constant, T is the sail's temperature at its radial center, and

$$A_\perp = \pi\left(\frac{d_s}{2}\right)^2 \tag{47}$$

is the perpendicular-to-laser beam area of the sail. The integral is shown to occur between two wavelengths $\langle\lambda_1, \lambda_2\rangle$ for which spectral information for the sail film is available and a majority of the emission is likely to take place according to Wien's displacement law [8].

We note that, in principle, the optical coefficients $\alpha_{\beta,\perp}$ and ε_λ are temperature-dependent. Due to a lack of experimental data over a sufficiently wide wavelength range at elevated temperatures, we have opted to adopt only room-temperature ($T = 300$ K) optical information (see Supplementary Note 8). We consider that this simplification is useful and reasonable given the other large uncertainties present in these simulations. However, future studies would greatly benefit from high-temperature optical properties (see Supplementary Note 17).

Supplementary Note 6:

Transfer-matrix method

The following section is copied, with minor editorial changes, from the Supporting Information for Campbell et al. [87].

Similarly to others [3, 46], we used the transfer-matrix method [87, 95, 96] to obtain optical properties (reflectivity and absorptivity/emissivity) for the thin sail films used in this work. Consider the structure shown in Supplementary Figure 16, which consists of ψ layers (including the space on both sides), where each mth layer has a thickness t_m, an angle of incidence relative to the normal direction θ_m, and a complex index of refraction $\mathfrak{n}_m = n_m + \mathrm{i}\kappa_m$ (here n is the real component, κ is the extinction coefficient, $\mathrm{i} = \sqrt{-1}$, and n and κ depend on the light wavelength λ). In the case of a light sail in the vacuum of space, the index of refraction of the first and ψ^{th} layers is $\mathfrak{n}_1 = n_1 + \mathrm{i}\kappa_1 = \mathfrak{n}_\psi = n_\psi + \mathrm{i}\kappa_\psi = 1 + 0\mathrm{i}$ and the thicknesses t_1 and t_ψ are semi-infinite. The value of θ_1 is equal to the angle of incidence upon the (possibly curved) sail, and the remaining complex angles ($2 \leq m \leq \psi$) [96, 97] can be calculated according to

$$\theta_m = \Re\left(\arcsin\left(\frac{\mathfrak{n}_{m-1}}{\mathfrak{n}_m}\sin(\theta_{m-1})\right)\right) - \mathrm{i}\left|\Im\left(\arcsin\left(\frac{\mathfrak{n}_{m-1}}{\mathfrak{n}_m}\sin(\theta_{m-1})\right)\right)\right|, \tag{48}$$

where $\Re$ and $\Im$ denote the real and imaginary components of a complex number, respectively, and the vertical bars $||$ signify the absolute value. Since, according to Kirchhoff's law of radiation, emissivity is equal to absorptivity at a given wavelength for a body in thermal equilibrium, for emission calculations the angle of exitance can be thought of as the angle of incidence for absorption.

The Fresnel equations for perpendicular ($\mathtt{s}$) and parallel ($\mathtt{p}$) polarizations for reflection (r) and transmission (t) can be used to

obtain the reflection and transmission coefficients in each layer:

$$r_{\mathrm{s},m} = \frac{\mathfrak{n}_m \cos(\theta_m) - \mathfrak{n}_{m+1} \cos(\theta_{m+1})}{\mathfrak{n}_m \cos(\theta_m) + \mathfrak{n}_{m+1} \cos(\theta_{m+1})} \tag{49}$$

$$t_{\mathrm{s},m} = \frac{2\mathfrak{n}_m \cos(\theta_m)}{\mathfrak{n}_m \cos(\theta_m) + \mathfrak{n}_{m+1} \cos(\theta_{m+1})} \tag{50}$$

$$r_{\mathrm{p},m} = \frac{\mathfrak{n}_m \cos(\theta_{m+1}) - \mathfrak{n}_{m+1} \cos(\theta_m)}{\mathfrak{n}_m \cos(\theta_{m+1}) + \mathfrak{n}_{m+1} \cos(\theta_m)} \tag{51}$$

$$t_{\mathrm{p},m} = \frac{2\mathfrak{n}_m \cos(\theta_m)}{\mathfrak{n}_m \cos(\theta_{m+1}) + \mathfrak{n}_{m+1} \cos(\theta_m)}. \tag{52}$$

In Equations 49-52, $1 \leq m \leq \psi - 1$. The phase shift factors Δ_m can be calculated using

$$\Delta_m = 2\pi \frac{t_m}{\lambda} \mathfrak{n}_m \cos(\theta_m) \tag{53}$$

for $2 \leq m \leq \psi - 1$. Transfer matrices for each polarization and layer can be formed according to

$$\mathbf{T}_{\mathrm{s},m} = \begin{bmatrix} \frac{1}{t_{\mathrm{s},m}} \exp(-\mathrm{i}\Delta_m) & \frac{r_{\mathrm{s},m}}{t_{\mathrm{s},m}} \exp(\mathrm{i}\Delta_m) \\ \frac{r_{\mathrm{s},m}}{t_{\mathrm{s},m}} \exp(-\mathrm{i}\Delta_m) & \frac{1}{t_{\mathrm{s},m}} \exp(\mathrm{i}\Delta_m) \end{bmatrix} \tag{54}$$

$$\mathbf{T}_{\mathrm{p},m} = \begin{bmatrix} \frac{1}{t_{\mathrm{p},m}} \exp(-\mathrm{i}\Delta_m) & \frac{r_{\mathrm{p},m}}{t_{\mathrm{p},m}} \exp(\mathrm{i}\Delta_m) \\ \frac{r_{\mathrm{p},m}}{t_{\mathrm{p},m}} \exp(-\mathrm{i}\Delta_m) & \frac{1}{t_{\mathrm{p},m}} \exp(\mathrm{i}\Delta_m) \end{bmatrix} \tag{55}$$

for $2 \leq m \leq \psi - 1$, and

$$\mathbf{T}_{\mathrm{s},\psi} = \begin{bmatrix} \frac{1}{t_{\mathrm{s},\psi}} & \frac{r_{\mathrm{s},\psi}}{t_{\mathrm{s},\psi}} \\ \frac{r_{\mathrm{s},\psi}}{t_{\mathrm{s},\psi}} & \frac{1}{t_{\mathrm{s},\psi}} \end{bmatrix} \tag{56}$$

$$\mathbf{T}_{\mathrm{p},\psi} = \begin{bmatrix} \frac{1}{t_{\mathrm{p},\psi}} & \frac{r_{\mathrm{p},\psi}}{t_{\mathrm{p},\psi}} \\ \frac{r_{\mathrm{p},\psi}}{t_{\mathrm{p},\psi}} & \frac{1}{t_{\mathrm{p},\psi}} \end{bmatrix} \tag{57}$$

for $m = \psi$. These layer-wise transfer matrices can be combined into a single transfer-matrix for the entire film stack according to

$$\mathbf{T}_{\mathrm{s},\theta_1} = \prod_{m=2}^{\psi} \mathbf{T}_{\mathrm{s},m} \tag{58}$$

$$\mathbf{T}_{\mathrm{p},\theta_1} = \prod_{m=2}^{\psi} \mathbf{T}_{\mathrm{p},m} \tag{59}$$

where the product $\prod$ implies right two-dimensional matrix multiplication

$$\begin{aligned} \mathbf{M}_m \mathbf{M}_{m+1} &= \begin{bmatrix} a_m & b_m \\ c_m & d_m \end{bmatrix} \begin{bmatrix} a_{m+1} & b_{m+1} \\ c_{m+1} & d_{m+1} \end{bmatrix} \\ &= \begin{bmatrix} a_m a_{m+1} + b_m c_{m+1} & a_m b_{m+1} + b_m d_{m+1} \\ c_m a_{m+1} + d_m c_{m+1} & c_m b_{m+1} + d_m d_{m+1} \end{bmatrix} \end{aligned} \tag{60}$$

and the polar angle subscript θ_1 indicates that these are calculated at the specified angle of incidence.

The total Fresnel coefficients for reflection and transmission are then

$$r_{\mathrm{s},\theta_1} = \frac{\mathbf{T}_{\mathrm{s},\langle 2,1\rangle}}{\mathbf{T}_{\mathrm{s},\langle 1,1\rangle}} \tag{61}$$

$$t_{\mathrm{s},\theta_1} = \frac{1}{\mathbf{T}_{\mathrm{s},\langle 1,1\rangle}} \tag{62}$$

$$r_{\mathrm{p},\theta_1} = \frac{\mathbf{T}_{\mathrm{p},\langle 2,1\rangle}}{\mathbf{T}_{\mathrm{p},\langle 1,1\rangle}} \tag{63}$$

$$t_{\mathrm{p},\theta_1} = \frac{1}{\mathbf{T}_{\mathrm{p},\langle 1,1\rangle}}, \tag{64}$$

where the bracketed subscript indices $\langle i, j\rangle$ indicate the row and column in the matrix, respectively. Finally, the total angular spectral reflectivity ϱ, transmissivity τ, and absorptivity α in intensity can be obtained using

$$\varrho_{\mathrm{s},\lambda,\theta_1,\phi} = |r_{\mathrm{s},\theta_1}|^2 \tag{65}$$

$$\tau_{\mathrm{s},\lambda,\theta_1,\phi} = |t_{\mathrm{s},\theta_1}|^2 \frac{\Re(\mathfrak{n}_\psi \cos(\theta_\psi))}{\Re(\mathfrak{n}_1 \cos(\theta_1))} \tag{66}$$

$$\alpha_{\mathrm{s},\lambda,\theta_1,\phi} = 1 - \varrho_{\mathrm{s},\lambda,\theta_1,\phi} - \tau_{\mathrm{s},\lambda,\theta_1,\phi} \tag{67}$$

$$\varrho_{\mathrm{p},\lambda,\theta_1,\phi} = |r_{\mathrm{p},\theta_1}|^2 \tag{68}$$

$$\tau_{\mathrm{p},\lambda,\theta_1,\phi} = |t_{\mathrm{p},\theta_1}|^2 \frac{\Re(\mathfrak{n}_\psi \cos(\theta_\psi))}{\Re(\mathfrak{n}_1 \cos(\theta_1))} \tag{69}$$

$$\alpha_{\mathrm{p},\lambda,\theta_1,\phi} = 1 - \varrho_{\mathrm{p},\lambda,\theta_1,\phi} - \tau_{\mathrm{p},\lambda,\theta_1,\phi}, \tag{70}$$

where here we have added the aziumuthal angle ϕ as a subscript to indicate that these are spectral-specular values. In practice, the data produced by the transfer-matrix method in planar-isotropic films are azimuthally symmetric.

Supplementary Note 7: Optical property calculations

7.1. Polarization

The transfer-matrix method yields both perpendicular (s) and parallel (p) polarizations for the reflectivity ϱ, transmissivity τ, and absorptivity α. We assume that the incoming laser light is unpolarized, and as such, we average the s and p data to obtain single values for reflection and absorption.

$$\varrho_{\beta,\theta,\phi} = \frac{\varrho_{\mathrm{s},\lambda_s,\theta,\phi} + \varrho_{\mathrm{p},\lambda_s,\theta,\phi}}{2} \tag{71}$$

$$\alpha_{\beta,\theta,\phi} = \frac{\alpha_{\mathrm{s},\lambda_s,\theta,\phi} + \alpha_{\mathrm{p},\lambda_s,\theta,\phi}}{2} \tag{72}$$

The emitted light is also unpolarized, allowing us likewise to average the s and p components. In addition, according to Kirchhoff's law of radiation, we equate the film's emissivity and absorptivity by assuming the sail is in thermal equilibrium.

$$\varepsilon_{\lambda,\theta,\phi} = \alpha_{\lambda,\theta,\phi} = \frac{\alpha_{\mathrm{s},\lambda,\theta,\phi} + \alpha_{\mathrm{p},\lambda,\theta,\phi}}{2} \tag{73}$$

7.2. Reflectivity

For a flat two-dimensional isotropic sail oriented perpendicular to the incoming photons, the average reflectivity is equal to the normal reflectivity at any point on the sail:

$$\varrho_{\beta,a}^{flat} = \varrho_{\beta,\perp}. \tag{74}$$

The perpendicular-to-sail reflectivity is obtained using $\theta = 0$ rad in Equation 71 (for $\theta = 0$ rad, the ϕ-component is eliminated):

$$\varrho_{\beta,\perp} = \varrho_{\beta,\theta=0,\phi}. \tag{75}$$

In contrast, the reflectivity for curved sails, which have better laser-beam-riding stability and feature lower mechanical stresses than their flat counterparts [87, 98–100], is impacted by their off-normal-axis reflectivity values and angle-dependent photon reflection directions [87]. We can account for these two factors using

$$\varrho_{\beta,a} = \frac{4}{\pi}\left(\frac{s_s}{d_s}\right)^2 \int_0^{2\pi}\int_0^{\theta_{max}} \varrho_{\beta,\theta,\phi}\cos^3(\theta)\sin(\theta)d\theta d\phi, \tag{76}$$

where θ is the angular polar coordinate (measured relative to the incident laser light), ϕ is the aziumuthal angle, $\varrho_{\beta,\theta,\phi}$ is the angle-dependent reflectivity of the surface when the sail's relative velocity is β, and

$$\theta_{max} = \arcsin\left(\frac{d_s}{2s_s}\right). \tag{77}$$

It is expedient to compare the different sail films by examining their average normal (perpendicular-to-surface) reflectivity within the applicable Doppler-shifted wavelength range corresponding to their acceleration from $\beta = 0$ to $\beta = 0.2$. We calculate this value according to

$$\overline{\varrho_\perp} = \frac{\int_{\lambda_l}^{\lambda_f} \varrho_{\lambda,\perp}d\lambda}{\lambda_f - \lambda_l}, \tag{78}$$

where λ_l is the laser output wavelength (*i.e.*, that which hits the sail at $\beta = 0$) and λ_f is the wavelength of the photons hitting the sail when $\beta = 0.2$ (Equation 30). Note that the integral in Equation 78 is within λ space, whereas other integrals in this work (*e.g.*, Equation 36) are in β space. The two formulations produce slightly different average values due to differences in the wavelength spacing between evenly-spaced β and λ points. Campbell *et al.* [87] selected the β-basis, whereas here we use the λ-basis.

7.3. Absorptivity

For the thermal energy balance calculation, we used the normal (perpendicular-to-sail-surface) absorptivity in order to conservatively estimate the sail temperature (the absorptivity in the normal direction is usually the largest). These values are simply the $\theta = 0$ absorptivities (there is no ϕ-contribution for $\theta = 0$) provided by the transfer matrix method.

$$\alpha_{\beta,\perp} = \alpha_{\beta,\theta=0,\phi} \tag{79}$$

Estimating the absorptivity of the sails in the literature against which we compared our designs was complicated by the fact that several contained patterned geometric elements not captured well by the transfer matrix method. Therefore, for the sail comparison of Figure 4 of the main article, for designs that did not provide experimental reflectivity and transmissivity measurements, we estimated the absorptivity of the designs using

$$\begin{aligned}\alpha_{\lambda,\perp} &= 1 - \prod_{i=1}^{n}(1-\alpha_{\lambda,\perp,i}) \\ &\approx \sum_{i=1}^{n}\alpha_{\lambda,\perp,i} \\ &= \sum_{i=1}^{n}\frac{4\pi\kappa_{\lambda,i}t_{f,i}F_i}{\lambda},\end{aligned} \tag{80}$$

where $\alpha_{\lambda,\perp,i}$ is the absorptivity of the ith layer (n total layers) at wavelength λ, $\kappa_{\lambda,i}$ is the extinction coefficient of the ith layer at wavelength λ, $t_{f,i}$ is the ith layer's thickness, and F_i is the layer's material fill factor, estimated using the given geometric pattern of the sail (see Supplementary Note 14).

We calculated the laser-band average absorptivities by integrating in λ space:

$$\overline{\alpha_\perp} = \frac{\int_{\lambda_l}^{\lambda_f}\alpha_{\lambda,\perp}d\lambda}{\lambda_f - \lambda_l}, \tag{81}$$

7.4. Emissivity

We obtain the spectral hemispherical emissivity according to

$$\varepsilon_\lambda = \frac{\int_0^{2\pi}\int_0^{\frac{\pi}{2}}\varepsilon_{\lambda,\theta,\phi}\sin(\theta)\cos(\theta)d\theta d\phi}{\pi}, \tag{82}$$

where of course $\int_0^{2\pi} d\phi = 2\pi$. This calculation involves integrating emissivity (*i.e.*, absorptivity) data calculated using the transfer-matrix method over the polar angle θ and the azimuthal angle ϕ. A full derivation for this equation and other emissivity equations is presented in the Supporting Information for Campbell *et al.* [87].

It is useful to compare the emissivity values of different sail designs, and for this purpose we calculate the effective emissivity:

$$\varepsilon_e = \frac{\int_{\lambda_1}^{\lambda_2}\varepsilon_\lambda I_{b,\lambda}d\lambda}{\int_{\lambda_1}^{\lambda_2}I_{b,\lambda}d\lambda}. \tag{83}$$

The integration is performed over the wavelength range for which optical information is available $\langle\lambda_1, \lambda_2\rangle$, which ideally should include a majority of the emission (this can be estimated using Wien's displacement law [8]). The term $I_{b,\lambda}$ is the black-body emission intensity, calculated according to

$$I_{b,\lambda} = \frac{2hc^2}{\lambda^5\left(\exp\left(\frac{hc}{\lambda k_B T}\right) - 1\right)}, \tag{84}$$

where h is Planck's constant, k_B is Boltzmann's constant, and T is the sail's temperature. For the purpose of comparing the effective emissivity values of several sail films, we used a consistent temperature of $T = 1000$ K (but, due to the limited amount of data

available, we used refractive index information at roughly 300 K). The exitance of the sail in its radial center $\mathcal{E}_s$ can be determined through

$$\mathcal{E}_s = 2\int_{\lambda_1}^{\lambda_2} \pi\varepsilon_\lambda I_{b,\lambda} d\lambda, \tag{85}$$

where the factor of two denotes that emission can occur from both sides of the sail. For sail designs that are not symmetric across their thicknesses, the emissivity values of the front (facing the laser photons, ε_f) and back (facing away from the laser photons, ε_b) sides are different. In this case, we simply calculate the average hemispherical emissivity spectrum

$$\varepsilon_{\lambda,a} = \frac{\varepsilon_{\lambda,f} + \varepsilon_{\lambda,b}}{2} \tag{86}$$

and average effective emissivity

$$\varepsilon_{e,a} = \frac{\varepsilon_{e,f} + \varepsilon_{e,b}}{2} \tag{87}$$

and use these in place of ε_λ and ε_e, respectively.

Supplementary Note 8: Spectroscopic data

Supplementary Figure 17 provides a comparison of the index of refraction $\mathfrak{n} = n + i\kappa$ that we measured for a MoS_2 film that we fabricated with others reported in the literature [1–4]. This film in particular is that which was part of the fabricated prototype whose reflectivity and transmissivity are shown in Figure 3(c) of the main article. Peaks in the n data are evident near wavelengths of $\lambda = 675$ nm and 625 nm in all six records, indicating the presence of strong A and B excitons, respectively. Supplementary Figure 18 shows the indices of refraction measured for two other films that we fabricated. We were able to grow large-area MoS_2 with a refractive index as large as $n = 4.25$ at $\lambda = 1.5$ µm, which is comparable to the value observed in exfoliated crystals [2, 4]. This demonstrates the potential for high-quality, wafer-scale growth of MoS_2. The reduced refractive index in the record corresponding to our prototype is most likely caused by an increased number of voids within the MoS_2 layer, since both n and κ are reduced, whereas κ would increase below the band gap (1.2-1.8 eV) if the variation was the result of imperfections in the crystal [101]. In order to calculate the emissivity of our fabricated prototype, we used a Cauchy model (with the first two terms only) and an Urbach-tail extension to extrapolate our measured n and κ data, respectively, to longer wavelengths [102], as shown in Supplementary Figure 19 (see Supplementary Note 14). To estimate the emissivity of our proposed optimized design, we used MoS_2 data from Munkhbat *et al.* [4], similarly extrapolated. Other data for MoS_2 are also available in the literature, and generally agree with the results displayed here [10, 103–109].

Supplementary Figure 20 shows our measured index of refraction information for Al_2O_3 in comparison to those reported by Lingart, Petrov, and Tikhonova [5] at ($T = 300$ K), Querry [6], and Kischkat *et al.* [7] at wavelengths near the laser band ($\lambda = \langle 1.2, 1.4697\rangle$ µm). Both the n and κ data are relatively constant in this range, although there are significant differences between the κ values from different sources. For simulating the optimized film's optical properties for the comparison in Figure 4 in the main article, we extrapolated the data of Kischkat *et al.* [7] back to the laser band; we selected this source because it is more recent than the prior sources, its extinction coefficients fall within the reported range of literature values, and its n values closely match those that we measured.

The Al_2O_3 data of Lingart, Petrov, and Tikhonova [5] is compiled from several sources, including References [110–115]. Though it contains information up to $T = 2300$ K, we used only the $T = 300$ K data because elevated-temperature information was not also available at wavelengths longer than 7 µm, nor were high-temperature spectroscopic properties available for MoS_2. The absorption data in this paper are provided as Naperian attenuation coefficients ϖ_λ, which we converted to extinction coefficients κ_λ through

$$\kappa_\lambda = \frac{\varpi_\lambda \lambda}{4\pi}, \tag{88}$$

where λ is the light wavelength. We attach the subscript λ to emphasize that the index of refraction parameters are wavelength-dependent.

The report by Querry [6] presents both ordinary (o) and extraordinary (e) parts of the refractive index for Al_2O_3, which we averaged according to

$$n_\lambda = \frac{n_{\mathsf{o},\lambda} + n_{\mathsf{e},\lambda}}{2} \tag{89}$$

and

$$\kappa_\lambda = \frac{\kappa_{\mathsf{o},\lambda} + \kappa_{\mathsf{e},\lambda}}{2}. \tag{90}$$

Other references for Al_2O_3 exist and generally agree with the works we have included in Supplementary Figure 20, although we have not attempted to conduct an exhaustive comparison here [7, 116–125]. See also Supplementary Note 17 for limited temperature-dependent optical property information.

Supplementary Figure 21 shows longer-wavelength index of refraction information for Al_2O_3, which is particularly useful in judging its performance as a thermal emitter. We have included several unity-normalized Planck black-body curves, calculated according to Equation 84, to show how the emission shifts with temperature. Although the peaks at higher temperatures correspond to wavelengths where the extinction coefficient of Al_2O_3 is not as large, the overall exitance can still be significant because the peak of the black-body radiation curve increases dramatically in magnitude. See Supplementary Note 17 for additional information on thermal considerations for the light sails.

To simulate the optical properties of the other films reported in Figure 4 of the main article, we drew upon indices of refraction reported in the literature (see Supplementary Note 14): MoS_2 [4], Si [126, 127], SiO_2 [7, 128], Si_3N_4 [7], and TiO_2 [7].

Supplementary Note 9: Laser reflection and transmission measurements

Here we present additional details about the laser reflection and transmission measurements. All laser characterization measurements were conducted at the California Institute of Technology in Pasadena, CA; we designed special fixtures to ship chips with fully suspended films to there from the University of Pennsylvania in Philadelphia, PA. In our experiments, we coupled the output of a supercontinuum white light laser source (SuperK Fianium FIU-15) to a grating monochromator to produce wavelength-tunable, monochromatic light to illuminate our suspended test films in the wavelength range $\lambda = \langle 1000, 1600 \rangle$ nm (Supplementary Figure 22). We used an optical chopper at a frequency of 417 Hz to modulate the light, enabling us to make phase-sensitive measurements and reject stray radiation. Moreover, we used a wire-grid linear polarizer to polarize the laser beam in the perpendicular (s) direction. Note that, for light normally incident on a surface, the perpendicular (s) and parallel (p) transmissivity values are equal, and the (s) and (p) reflectivity values are equal. After the monochromator, our beam path included lenses, parabolic mirrors, and apertures to collimate and expand the monochromatic laser beam, filling the objective's rear aperture (20× M Plan APO NIR, numerical aperture $NA = 0.4$, Mitutoyo) for a focused laser spot on the sample ($<$ 10 µm in diameter; see Supplementary Figure 23). Note that this optic collects photons at angles ranging from normal incidence to roughly 23.6°. We conducted transfer-matrix method simulations using our measured indices of refraction and found that the reflectivity and absorptivity of the fabricated prototype design vary by at most 1% and 1.2%, respectively, within the laser band ($\lambda = \langle 1.2, 1.4697 \rangle$ µm) and the $NA = 0.4$ angular range ($0 \leq \theta \leq 23.6°$); see Supplementary Figure 27. This suggests that the experimental results can be regarded as normal-incidence data. We clamped the chip containing the suspended film vertically on a stage for $X - Y$ translation and single-degree-of-freedom rotation (incidence angle). We included a beam splitter prior to the objective lens to allow us to collect the reflected light for imaging onto a camera or for detecting the reflected intensity using a Ge photodetector. Importantly, our use of this beamsplitter allowed us to measure the normal-incidence (*i.e.*, 0°) reflectivity. We normalized the reflection measurements using the reflection from a template-stripped, flat Au sample on Si, whose wavelength-dependent reflectivity we calculated using the transfer-matrix method based on literature-obtained indices of refraction [129, 130]. For transmission measurements, we placed the Ge photodetector behind the sample. We obtained the dark current measurements (no light) by moving the sample out of the beam path with motorized stages. During all measurements, we took a reference measurement of the laser beam source simultaneously in order to normalize out intensity fluctuations. We amplified and biased the detected signals (reference, reflection, and transmission) using transimpedance amplifiers and ultimately measured the signals using lock-in amplifiers. We measured the reflectivity and transmissivity at three spots on the film (henceforth denoted A, B, and C) and averaged the results.

We analyzed the laser data as follows. We calculated the wavelength-dependent transmissivity using

$$\tau_\lambda = \frac{T_{\lambda,f}}{T_{\lambda,100}}, \tag{91}$$

where $T_{\lambda,f}$ is the normalized transmission detector signal with the film in place,

$$T_{\lambda,f} = \frac{J_{\lambda,f}}{J_{\lambda,f,r}}, \tag{92}$$

and $T_{\lambda,100}$ is the normalized transmission detector signal with the film shifted out of the beam path,

$$T_{\lambda,100} = \frac{J_{\lambda,100}}{J_{\lambda,100,r}}. \tag{93}$$

Here J indicates the current produced by the photodetector and subscript r denotes the reference detector measurement used for normalization of the laser intensity fluctuations.

We calculated the wavelength-dependent reflectivity according to

$$\varrho_\lambda = \frac{R_{\lambda,f} - R_{\lambda,0}}{R_{\lambda,100} - R_{\lambda,0}}. \tag{94}$$

Here, $R_{\lambda,f}$ is the normalized reflection detector signal with the film in place

$$R_{\lambda,f} = \frac{J_{\lambda,f}}{J_{\lambda,f,r}}, \tag{95}$$

$R_{\lambda,0}$ is the normalized reflection detector signal with the film shifted out of the beam path

$$R_{\lambda,0} = \frac{J_{\lambda,0}}{J_{\lambda,0,r}}, \tag{96}$$

and $R_{\lambda,100}$ is the normalized reflection detector signal with the Au reference chip in place of the film, normalized by the calculated reflectivity for the chip $\varrho_{\lambda,c}$

$$R_{\lambda,100} = \left(\frac{J_{\lambda,100}}{J_{\lambda,100,r}}\right)\left(\frac{1}{\varrho_{\lambda,c}}\right). \tag{97}$$

We estimated a $\pm 3\%$ uncertainty for the reflectivity and transmissivity values, which we applied to each of the three spots that we measured. This uncertainty is attributable to the mechanical instability of the monochromator, to mechanical and thermal drifts of components in the optical path, to non-perfect beam forming and illumination, to noise in the detection electronics, and the nature of non-single-shot reflection and transmission measurements. To obtain the error bars shown in the main article, we determined the maximum and minimum values among the three datasets, *e.g.*, $\tau_\lambda^{max} = \max\left(\tau_{\lambda,A}^{+}, \tau_{\lambda,B}^{+}, \tau_{\lambda,C}^{+}\right)$ or $\varrho_\lambda^{min} = \min\left(\varrho_{\lambda,A}^{-}, \varrho_{\lambda,B}^{-}, \varrho_{\lambda,C}^{-}\right)$, where here the $+$ and $-$ superscripts indicate the $+3\%$ and -3% uncertainty values, respectively. We compounded these errors for the absorptivity uncertainty values, *e.g.*, $\alpha_\lambda^{max} = 1 - \tau_\lambda^{min} - \varrho_\lambda^{min}$.

Finally, we note that each measurement was made with the laser spot focused in the center of one of the corrugated hexagon areas, rather than on the perimeter of a hexagon near the vertical walls. This was a practical decision on our part in order to simplify the experiments. In a practical light sail, laser light would naturally be incident upon these vertical components, which present a longer path length for absorption than the planar film areas. Here we offer several comments. First, as discussed in Supplementary Note 18, the vertical edges may scatter light, such that the number of photons available to be absorbed may be smaller than the full incident amount. Second, due to the nanoscale thicknesses of the vertical components, detailed numerical simulations accounting for the three-dimensional hexagonal corrugated film structure are necessary to fully quantify the absorption; these are beyond the scope of this work. Third, for the more immediately achievable goal of intra-solar-system light sail travel in which lower laser powers were required, incident photon fluxes on the sail would conceivably not be high enough to cause damage, even if the vertical corrugated components exhibited enhanced absorption. Fourth, we have developed an alternative corrugated architecture in which planar MoS_2 films are wet chemical transferred onto the tops of Al_2O_3-coated trenches (Supplementary Figure 24) [9]. This implementation reduces the amount of absorbing material in the vertical components, thereby mitigating the risk of excessive absorption there.

Supplementary Note 10: Optical requirements and thermally-limited power

Here we estimate the required ratio of infrared emissivity to laser band absorptivity for the sail film. We assume to first order that at all points throughout its acceleration, the sail will be in thermal equilibrium such that any energy it absorbs (per unit area) it also must radiate out. We perform this estimate on a per-unit-area basis because the lateral thermal conductivity of nanometer-thick sails will likely be so low that, for curved sails, the center region (perpendicular to the incident laser photons) may be significantly hotter than the perimeter areas [100].

$$e_{absorb} = e_{radiate} \tag{98}$$

The energy absorbed from the incident laser photons per unit area can be estimated by

$$e_{absorb} = \frac{\overline{\alpha_\perp}\Phi_s}{A_\perp} \tag{99}$$

where $\overline{\alpha_\perp}$ is the laser band-average absorptivity (Equation 81), Φ_s is the instantaneous incident photon power hitting the sail, and $A_\perp$ is the area of the sail perpendicular to the incident laser beam (Equation 47). The energy radiated per unit area can be estimated by

$$e_{radiate} = 2\varepsilon_e \sigma T^4 \tag{100}$$

where ε_e is the hemispherical effective emissivity of the sail at $T =$ 1000 K (Equation 83), σ is the Stefan-Boltzmann constant, T is the sail temperature, and the factor of two indicates that radiation can occur from both sides of the sail film. Equating Equations 99 and 100 and solving for the ratio $\frac{\varepsilon_e}{\overline{\alpha_\perp}}$ gives

$$\frac{\varepsilon_e}{\overline{\alpha_\perp}} = \frac{\Phi_s}{2A_\perp \sigma T^4}. \tag{101}$$

At a laser power of $\Phi_s = 100$ GW (when the $\beta = 0$, the power incident on the sail is equal to the laser output power; see Equation 35), a sail perpendicular-to-laser area of $A_\perp = 1\ \mathrm{m}^2$, and a reasonable maximum sail temperature of $T = 1000$ K (at which point the sail would likely begin to sublime or melt [48]), the ratio becomes roughly $\frac{\varepsilon_e}{\overline{\alpha_\perp}} \approx 10^6$. Since the emissivity of films this thin is likely to be $\varepsilon_e \sim 10^{-3}$, it is essential that the laser band absorptivity be $\overline{\alpha_\perp} \sim 10^{-9}$, or virtually zero.

Similarly, we can estimate the maximum power that a sail can tolerate, given an emissivity-absorptivity ratio $\frac{\varepsilon_e}{\overline{\alpha_\perp}}$, a maximum material temperature T_{max}, and a perpendicular-to-laser area $A_\perp$.

$$\Phi_{s,max} = 2\frac{\varepsilon_e}{\overline{\alpha_\perp}} A_\perp \sigma T_{max}^4 \tag{102}$$

In our calculations, we have used a consistent temperature of $T = 1000$ K to calculate the effective emissivity (Equation 83), although some sails could tolerate higher maximum temperatures before melting or vaporizing. This simplification is reasonable given other uncertainties, such as the temperature dependence of the indices of refraction of the sail materials (see Supplementary Note 17).

According to Equation 102, sails with higher emissivity-absorptivity ratios can tolerate higher laser power values. In addition, the fourth-order dependence on the material temperature suggests that sails that have higher thermal limits will realize substantial accelerative improvements. Finally, sail films that have lower areal densities can be made to be larger for the same total sail mass, reducing the optical intensity (power per area) at any point on the sail and allowing higher overall laser powers to be used.

We can use the emissivity-to-absorptivity ratio $\frac{\varepsilon_e}{\overline{\alpha_\perp}} \approx 10^6$ to estimate the minimum areal density achievable for our class of nanolaminate sails when optimizing solely for thermal considerations. We begin by using Equation 80 to estimate the absorptivity and emissivity (by Kirchhoff's law of thermal radiation) as a function of the film thickness.

$$\frac{\varepsilon_e}{\overline{\alpha_\perp}} \sim \frac{\frac{4\pi}{\lambda_{radiate}}\left(2\kappa_{A,radiate} t_{f,A} F_A + \kappa_{M,radiate} t_{f,M} F_M\right)}{\frac{4\pi}{\lambda_{absorb}}\left(2\kappa_{A,absorb} t_{f,A} F_A + \kappa_{M,absorb} t_{f,M} F_M\right)} \tag{103}$$

Here, subscripts A and M represent Al_2O_3 and MoS_2, respectively; $\lambda_{radiate}$ and λ_{absorb} are characteristic wavelengths for radiation (*e.g.*, in the infrared) and absorption (*e.g.*, the laser wavelength), respectively; $\kappa_{radiate}$ and κ_{absorb} are the characteristic extinction coefficients for radiation and absorption, respectively; t_f is the film thickness; F is a layer fill factor; and for simplicity we assume the top and bottom alumina layers have the same thickness. Rearranging, letting $F_A = F_M = 1$ (*i.e.*, complete and uniform material coverage with no holes), and solving for the film thickness ratio

$\frac{t_{f,M}}{t_{f,A}}$ gives

$$\frac{t_{f,M}}{t_{f,A}} \sim \frac{2\frac{\varepsilon_e}{\alpha_\perp}\frac{\lambda_{radiate}}{\lambda_{absorb}}\kappa_{A,absorb} - 2\kappa_{A,radiate}}{\kappa_{M,radiate} - \frac{\varepsilon_e}{\alpha_\perp}\frac{\lambda_{radiate}}{\lambda_{absorb}}\kappa_{M,absorb}}. \tag{104}$$

The equation only has meaningful solutions for $\frac{t_{f,M}}{t_{f,A}} > 0$, which places constraints on the optical values selected. To find the minimum possible areal density under thermal constraints we select optimistic values (see Supplementary Note 15) of $\kappa_{A,absorb} = \kappa_{M,absorb} = \kappa_{A,radiate} = 10^{-8}$ (the latter because we have not observed emissive features in MoS_2 at longer wavelengths) and $\kappa_{A,radiate} = 10^{-1}$, and select $\lambda_{absorb} = \lambda_l = 1.2$ µm and $\lambda_{radiate} = 2.9$ µm (the peak of the blackbody spectrum at $T = 1000$ K according to Wien's displacement law [8]). Using these values, we find $\frac{t_{f,M}}{t_{f,A}} \sim 6.3$. A minimum achievable thickness of Al_2O_3 on MoS_2 by atomic layer deposition is roughly $t_{f,A} \sim 1.5$ nm [63], leading to a minimum MoS_2 thickness of $t_{f,M} \sim 9.5$ nm. Finally, noting that the lowest-areal-density sails will occupy the limit where the rib/trench height is zero, these thicknesses (one MoS_2 sheet with two Al_2O_3 face sheets) lead to $\rho_{a,minimum} \sim 0.058\ \mathrm{g \cdot m^{-2}}$ (Equation 2). This limit is well within the $\rho_a \sim 0.1\ \mathrm{g \cdot m^{-2}}$ goal set by the Breakthrough Starshot Foundation [88, 92, 131, 132].

Supplementary Note 11: Strength analysis

The photon pressure (Equation 45) experienced by the sail will cause stress and strain in the sail film. For a thin spherically-curved sail with thickness t_f and radius of curvature s_s experiencing a pressure P, the membrane stress σ_s can be approximated by [87, 133–136]

$$\sigma_s = \frac{P s_s}{2 t_f}. \tag{105}$$

(See Supplementary Figure 25 for an explanation of the sail diameter and radius of curvature.) More complicated analyses can be done for the stress in multilayer nanolaminate films [75, 76], but the simple form above is expedient for analyses in which the uncertainty of many other variables (*e.g.*, the laser power, the sail temperature, the sail's optical properties, *etc.*) is relatively large. Rearranging Equation 105 produces

$$\sigma_s t_f = \frac{P s_s}{2}, \tag{106}$$

where we define the right side of this expression as the photon-induced tension,

$$\Upsilon = \frac{P s_s}{2}. \tag{107}$$

This quantity, with dimensions force per unit length, can be thought of as the force acting to create a tear of some length in the sail. At the verge of failure (sail tearing), the membrane stress will be equal to the tensile yield stress σ_y of the material, *i.e.*, $\sigma_s = \sigma_y$. Making this substitution in Equation 106 gives

$$\sigma_s t_f = \sigma_y t_f = \Upsilon. \tag{108}$$

We can extend the left side of Equation 108 to be applicable to composite materials using the rule of mixtures [77–79]. The rule of mixtures states that the yield stress of a composite film $\sigma_{y,c}$ composed of n layers can be estimated by

$$\sigma_{y,c} = \sum_{i=1}^{n} f_i \sigma_{y,i}, \tag{109}$$

where, if we define the total composite film thickness to be

$$t_c = \sum_{i=1}^{n} t_{f,i} \tag{110}$$

(see also Equation 10), the fractional composition of the ith component can be obtained by

$$f_i = \frac{t_{f,i}}{t_c} \tag{111}$$

(see also Equations 12 and 13). Multiplying Equation 109 by t_c and simplifying, we obtain

$$\begin{aligned} t_c \sigma_{y,c} &= t_c \sum_{i=1}^{n} f_i \sigma_{y,i} \\ &= t_c \sum_{i=1}^{n} \frac{t_{f,i}}{t_c} \sigma_{y,i} \\ &= \sum_{i=1}^{n} t_{f,i} \sigma_{y,i}. \end{aligned} \tag{112}$$

Equation 112 allows us to calculate the product of the unified composite thickness and its unified tensile yield strength from the thicknesses and yield strengths of its individual layers and materials. We define the right side of this equation as the membrane mechanical robustness,

$$\wp = \sum_{i=1}^{n} G_i t_{f,i} \sigma_{y,i}, \tag{113}$$

where G_i are geometrical parameters to account for the presence of holes or other architected features in the film layers, discussed below. Using this result in Equation 108, in our simulations, we defined mechanical failure of the film at the point where

$$\wp = \Upsilon. \tag{114}$$

The geometrical parameters can be calculated using Equation 115. For films that are planar or have repeating sections that are planar, G_i are taken as unity. For periodic films with holes of diameter d_{hole} and period $a_{pattern}$, G_i are the fractional connected lengths.

$$G_i = \begin{cases} 1 & \text{planar} \\ \frac{a_{pattern} - d_{hole}}{a_{pattern}} & \text{periodic holes} \end{cases} \tag{115}$$

Finally, for reference, we calculated the membrane mechanical robustness for monolayer graphene to be about $44\ \mathrm{N \cdot m^{-1}}$ [137], and that for a sheet of standard letter paper to be roughly $3\ \mathrm{kN \cdot m^{-1}}$ [138].

Supplementary Note 12: Calculation of maximum relative velocity

Here we explain our procedure for calculating the maximum relative velocity achievable by a given film design, β_{max}. We will provide specific details about the inputs to this method, such as areal density and laser output wavelength, for each sail film that we evaluated [46–54], in Supplementary Note 14.

For a given sail film, we began by using its material thickness, architecture, and yield stress information to calculate its areal density (simply denoted ρ_a here) and membrane mechanical robustness $\wp$ (Equation 113). We then used this areal density to calculate the surface area of one side of a spherically curved sail with a mass of $m_s = 1$ g:

$$A_s = \frac{m_s}{\rho_a} \tag{116}$$

Next, we used this area to calculate the diameter d_s of a spherically-curved circular sail that has a spherical radius of curvature equal to its diameter, $s_s = d_s$. See Supplementary Figure 25 for an explanation of the sail diameter and radius of curvature.

$$d_s = s_s = \sqrt{\frac{A_s}{\pi\left(2-\sqrt{3}\right)}} \tag{117}$$

Equation 117 can be derived from Equation S44 in the Supporting Information for Campbell *et al.* [87] by setting $d_s = s_s$. The condition $s_s \approx d_s$ is optimal for curved light sails [87]. We next used this sail diameter to estimate the maximum diffraction-limited distance L_{max} over which the sail could be accelerated with the laser perfectly focused on the full sail area for an Earth-bound laser array diameter $d_{l,E} = 30$ km and a constant laser output wavelength λ_l [89]:

$$L_{max} = \frac{d_{l,E} d_s}{2\lambda_l}. \tag{118}$$

We obtained this expression from Equation 44 in this document. Notice that, given the mass constraint, the sail diameter is inversely proportional to the areal density, which implies sails with higher areal densities have shorter acceleration "runways" L. This is one constraining factor on β_{max}.

We next iteratively determined the maximum constant laser output power Φ_l that the sail could sustain without tearing as it accelerated out to a distance L, up to a maximum power of $\Phi_l = 100$ GW. We defined the tearing failure mode according to Equation 114, *i.e.*, at the point where the photon-induced tension Υ (Equation 107) would be equal to the film's membrane mechanical robustness $\wp$ (Equation 113).

$$\wp = \Upsilon. \tag{114}$$

In this calculation we assumed a total rest sailcraft mass (including sail, payload, and any connecting tethers) of $m_{tot} = 2$ g (*i.e.*, twice the sail mass); having approximately equal sail and payload masses is optimal for acceleration [46, 87, 89, 139].

It is instructive to obtain an expression for the photon-induced tension in terms of the laser power and other sail design parameters. We achieved this by substituting Equation 45 into Equation 107 and simplifying knowing that we chose $d_s = s_s$ for this analysis. In the following, we also substituted $\Phi_{l,\beta} = \Phi_l$, which states that the output power produced by the laser is constant.

$$\frac{Ps_s}{2} = \Upsilon = \frac{4\varrho_{\beta,\perp}\Phi_l}{\pi c d_s}\left(\frac{1-\beta}{1+\beta}\right). \tag{119}$$

The photon-induced tension is proportional to the film's laser band reflectivity $\varrho_{\beta,\perp}$ and the constant laser output power Φ_l, and inversely proportional to the sail diameter d_s. It also decreases as β increases. Since $\varrho_{\beta,\perp}$ and d_s are prescribed by the sail design, some films required that the laser power be throttled back to a value lower than 100 GW. For other films, a power greater than this could be tolerated, in which case we simply applied the maximum power of 100 GW.

Finally, given the maximum achievable laser power, we stepped through Equation 36 by incrementally increasing the relative velocity β until the distance the sail has traveled was equal to L_{max}. We defined the corresponding relative velocity as β_{max}.

One may observe that Equation 119, which we use to calculate the maximum power that the sail can sustain without tearing, depends on β. Thus, the power iteration step required calculating over a range of β values, the upper limit of which was initially an unknown. In practice, we set a high upper limit for β and later checked to ensure that, if the laser output power was required to be limited at some $\beta = \beta_{limit}$, that limit occurred for $\beta_{limit} \le \beta_{max}$.

Lastly, note that Equation 119 is based on the perpendicular reflectivity of the sail ($\varrho_{\beta,\perp}$), whereas Equation 36 uses the spatially-averaged reflectivity that accounts for the impact of the sail curvature ($\varrho_{\beta,a}$; see Supplementary Note 7.2). In in our β_{max} calculations, we used the perpendicular reflectivity in place of the average reflectivity. This was because, for many of the sails in Figure 4 of the main article, angle-dependent reflectivity information, necessary for calculating the spatially-averaged reflectivity, was not available. We expect this approximation to slightly inflate the β_{max} values calculated for some designs.

Supplementary Note 13: Information on film thickness optimization

We determined the Al_2O_3 and MoS_2 film thicknesses of our proposed optimized design using the following method. We first selected hexagonal corrugation dimensions that would increase the film's bending stiffness enhancement factor $\mathbb{B}$ (thereby making the film more resistant to wrinkling) and decrease its tensile stiffness reduction factor $\mathbb{T}$ (thereby making the film more stretchy), as described in Supplementary Note 3 and illustrated in Figure 3(e) in the main article and Supplementary Figure 15(a). We chose a hexagon diameter $d_h = 70$ µm, a trench width $w_t = 2$ µm, and a trench height $h_t = 3$ µm. Also, for simplicity, we added the constraint that the top and bottom Al_2O_3 thicknesses should be equal. For optical property information, to determine the best sail accelerative performance within state-of-the-art fabrication capabilities, we used Al_2O_3 index of refraction data from Kischkat *et al.* [7] and MoS_2 index of refraction data from Munkhbat *et al.* [4]. In doing so, we assumed that future process improvements would allow

multilayer MoS_2 films to be fabricated with optical quality equivalent to that of bulk MoS_2 crystals. Furthermore, we set the sail mass to $m_s = 1$ g and the total sailcraft mass (sail with payload chip and any tethers) to be $m_{tot} = 2$ g.

Next, we examined multiple film design combinations spanning Al_2O_3 film thicknesses $\langle 1 \leq t_{A,b} = t_{A,t} \leq 100 \rangle$ nm and MoS_2 film thicknesses $\langle 1 \leq t_M \leq 200 \rangle$ nm. For each, we calculated the areal density $\rho_{a,c,t}$ (Equations 3, 5, and 7, with $\rho_A = 3200\ \mathrm{kg{\cdot}m^{-3}}$ [15] and $\rho_M = 5060\ \mathrm{kg \cdot m^{-3}}$ [21]), membrane mechanical robustness $\wp$ (Equation 113 with $G_i = 1$ and tensile yield stress values $\sigma_{y,\mathrm{Al_2O_3}} = 2$ GPa [16, 17] and $\sigma_{y,\mathrm{MoS_2}} = 2.3$ GPa [13]), wavelength-dependent reflectivity within the Doppler-shifted laser band $\varrho_{\beta,\perp}$ (Equation 75), and maximum relative velocity β_{max} for a laser array diameter $d_{l,E} = 30$ km and a constant maximum laser output power of $\Phi_l = 100$ GW. As discussed in Supplementary Note 4, the value we used for the MoS_2 tensile yield stress assumes that future fabrication improvements will allow thick and strong crystalline films to be formed; our estimate is reasonable given comparable data for a similar material, $MoSe_2$ [82].

We considered the optimized design to be that which achieved the highest β_{max} value. Our model predicted that this design, with Al_2O_3 thickness $t_{A,b} = t_{A,t} \approx 19$ nm and MoS_2 thickness $t_M \approx 63$ nm), will achieve $\beta_{max} = 0.26$. Our proposed optimized film has a corrugated areal density $\rho_{a,c,t} = 0.51\ \mathrm{g{\cdot}m^{-2}}$, a bending stiffness enhancement factor of $\mathbb{B} = 890$, and a tensile stiffness reduction factor of $\mathbb{T} = 0.0033$. We note that the optimization procedure outlined here does not account for thermal constraints. Depending on the extinction coefficients of Al_2O_3 and MoS_2, thermal considerations might require reducing the laser power to prevent the sail from sublimating, which would consequently decrease β_{max}. Such an optimization would be more relevant if reliable spectroscopic data at elevated temperatures for Al_2O_3 and MoS_2 was available (see Supplementary Note 17). Alternatively, a different design could be optimized for its effective emissivity ε_e (Equation 83) rather than for β_{max}.

Contour plots showing the results of the film thickness optimization are provided in Supplementary Figure 26. The optimal design (with the highest β_{max} value) does not achieve the highest reflectivity within the design space, in part because it decreases its areal density by reducing its MoS_2 thickness. Notice that, according to Equation 118, the acceleration length L_{max} scales with the sail diameter d_s, which depends on the sail area A_s (Equation 117), which in turn depends on the areal density ρ_a (Equation 116). This explains why the shapes of the contours in Supplementary Figure 26(f) mirror those of the areal density in panel (b).

Supplementary Note 14: Comparison of films in the literature

14.1. Excluded designs

Figure 4 of the main article compares several light sail films introduced in the literature [46–54]. The data from this figure are presented in Supplementary Table 2. Other sail designs have been suggested, as well, which we excluded for a variety of reasons. Some were theoretical proposals [87, 99, 100, 140]; others were non-continuous designs where a membrane mechanical robustness $\wp$ was not definable [141, 142]; one was a preliminary design outside the feasible atmospheric transmission wavelength range [143]; and others only reflected strongly at a single wavelength rather than across the entire Doppler-shifted laser wavelength band [144, 145].

14.2. Ilic, Went, and Atwater (2018) [46]

The paper by Ilic, Went, and Atwater [46] contained several silica-based designs and used a laser output wavelength of $\lambda_l = 1.2$ µm. For comparison, we selected what the paper refers to as design "A11," which consisted of SiO_2 layers separated by air/vacuum gaps, due to its high reflectivity and high membrane mechanical robustness (see Table S1 in the Supplementary Information for that paper). We used the design's layer thicknesses and index of refraction information for SiO_2 from Rodriguez-de Marcos *et al.* [128] to calculate the wavelength-dependent reflectivity for this design using the transfer-matrix method (Supplementary Note 6), which we then averaged to determine the laser band ($\beta = 0$ to $\beta = 0.2$) average reflectivity $\overline{\varrho_\perp}$ (Equation 78). We used the same data in Equations 80 and 81 to estimate the laser-band-average absorptivity, with fill factors for the layers $F_i = 1$ since the layers in this design were planar.

We used spectroscopic information from Kischkat *et al.* [7] and the transfer-matrix method to determine the sail's spectral directional emissivity in the wavelength range $\lambda = \langle 2, 14 \rangle$ µm, and subsequently integrated this data at a temperature of $T = 1000$ K to find the film's effective emissivity ε_e (Equation 83). The wavelength range $\lambda = \langle 2, 14 \rangle$ µm includes a majority of the emissive power at $T = 1000$ K and conveniently allowed us to make use of the Kischkat *et al.* [7] datasets for Al_2O_3, Si_3N_4, SiO_2, and TiO_2 without extrapolation.

We calculated the membrane mechanical robustness $\wp$ using Equation 113, with geometrical parameters $G_i = 1$ and yield stress $\sigma_{y,\mathrm{SiO_2}} = 1.5$ GPa [37, 40]. We calculated the areal density ρ_a using

$$\rho_a = \sum_{i=1}^{n} \rho_i t_{f,i} \tag{120}$$

where ρ_i and $t_{f,i}$ are the density and thickness of each layer, respectively; in this case $\rho_{\mathrm{SiO_2}} = 2030\ \mathrm{kg \cdot m^{-3}}$ [39] and $\rho_{gap} = 0\ \mathrm{kg \cdot m^{-3}}$ (see Supplementary Table 1). We obtained the maximum relative velocity achievable for this design, β_{max}, using the method outlined in Supplementary Note 12 with the reflectivity profile that we calculated above. Notably, the relatively high membrane mechanical robustness value of this design allowed it to sustain the maximum laser power of $\Phi_l = 100$ GW in this simulation. Lastly, we estimated the thermally-limited power $\Phi_{s,max}$ using Equation 102, with $A_\perp$ obtained *via* Equation 47 (using the sail diameter d_s derived from the sail's areal density and a mass of $m_s = 1$ g, Equations 116 and 117) and $T_{max} = 1450$ K (limited by SiO_2 [41]).

Overall, this film achieved a maximum relative velocity of $\beta_{max} = 0.22$, exceeding the Breakthrough Starshot goal [88, 92, 131, 132]. It was able to accomplish this because

of its high reflectivity of $\overline{\varrho_\perp} = 0.79$ (allowing it to accelerate quickly) and high membrane mechanical robustness of $\wp = 1077\ \mathrm{N \cdot m^{-1}}$ (allowing it to tolerate the maximum laser power $\Phi_l = 100$ GW). The sail had, however, a relatively high areal density of $\rho_a = 1.46\ \mathrm{g \cdot m^{-2}}$, which decreased its mass-constrained area ($A_\perp = 0.64\ \mathrm{m^2}$). This small area reduced its maximum acceleration length (*i.e.*, the acceleration runway, Equation 118; $L_{max} = 11.3$ Gm), which precluded its maximum relative velocity from increasing further. Considerations for future work were that the film had required nanoscale vacuum gaps between the SiO_2 films that required spacers to maintain, and that the film's emissivity-to-absorptivity ratio of $\frac{\varepsilon_e}{\alpha_\perp} = 8.9$ would limit practical laser powers to only $\Phi_{s,max} = 2.8$ MW to avoid overheating.

In addition to the SiO_2-only designs, Ilic, Went, and Atwater [46] also developed sails with Si layers. We have excluded these because recent work indicates that such sails are susceptible to thermal runaway [84]. For completeness, we note that their design "B4" performed exceptionally well in our simulations, achieving $\beta_{max} = 0.33$ with an areal density of $\rho_a = 0.17\ \mathrm{g \cdot m^{-2}}$ and a laser-band average reflectivity of $\overline{\varrho_\perp} = 0.83$.

14.3. Salary and Mosallaei (2020) [47]

The paper by Salary and Mosallaei [47] proposed designs that featured protruding Si disks of differing diameters on top of a continuous Si-SiO_2 backbone film, and used a laser output wavelength of $\lambda_l = 1.3$ µm. For simplicity, we used the reflectivity information contained in Figure 2(b) of their paper [47] with a constant disk diameter of 300 nm, which we averaged to estimate the mean laser band reflectivity $\overline{\varrho_\perp}$ (Equation 78).

We estimated the absorptivity using Equations 80 and 81 with optical data from Poruba *et al.* [126] and Rodriguez-de Marcos *et al.* [128] for Si and SiO_2, respectively. We accounted for the protruding Si disks using fill factor

$$F_i = \frac{\pi \left(\frac{d_{disk}}{2}\right)^2}{a^2}, \tag{121}$$

where d_{disk} is the protruding disk diameter and a is the period of the pattern, and used a fill factor $F_i = 1$ for the connecting (continuous) 89-nm-thick Si and 60-nm-thick SiO_2 layers below.

We calculated the sail's emissivity as in Supplementary Note 14.2, using optical data from Franta *et al.* [127] and Kischkat *et al.* [7] for Si and SiO_2, respectively, along with an effective medium approximation [146]. We calculated the membrane mechanical robustness using Equation 113 with $G_i = 1$, accounting only for the strength of the backbone Si-SiO_2 film (not the Si disks), with yield stress values $\sigma_{y,\mathrm{Si}} = 2$ GPa [31–34] and $\sigma_{y,\mathrm{SiO_2}} = 1.5$ GPa [37, 40]. We estimated the sail's areal density by averaging the two extreme areal density values given in the paper [47] for the largest and smallest protruding Si disks. We calculated the sail's maximum relative velocity using the method outlined in Supplementary Note 12 with the reflectivity profile obtained from Figure 2(b) of their paper [47] as discussed above. Lastly, we estimated the thermally-limited power $\Phi_{s,max}$ as in Supplementary Note 14.2, using $T_{max} = 1450$ K (limited by SiO_2 [41]).

Overall, this film achieved a high maximum relative velocity of $\beta_{max} = 0.25$, which was made possible by its high reflectivity of $\overline{\varrho_\perp} = 0.76$ and moderate membrane mechanical robustness of $\wp = 268\ \mathrm{N{\cdot}m^{-1}}$. The sail also featured a noteworthy low areal density of $\rho_a = 0.54\ \mathrm{g{\cdot}m^{-2}}$, which allowed it to have a large mass-constrained area ($A_\perp = 1.74\ \mathrm{m^2}$) and thus a long maximum acceleration length ($L_{max} = 17.2$ Gm). Future work should examine the fabricability of the nanodisk structure and also attempt to increase the film's emissivity-to-absorptivity ratio above its current $\frac{\varepsilon_e}{\alpha_\perp} = 8.0$ value.

14.4. Brewer et al. (2022) [48]

Brewer *et al.* [48] proposed a three-layer Si_3N_4-MoS_2-Si_3N_4 composite film (thicknesses $t_{\mathrm{Si_3N_4},t} = 5$ nm, $t_{\mathrm{MoS_2}} = 90$ nm, and $t_{\mathrm{Si_3N_4},b} = 5$ nm) with patterned holes (diameter $d_{hole} = 1044$ nm, x-period $a_x = 2009$ nm, and y-period $a_y = 1160$ nm). They specified a laser output wavelength of $\lambda_l = 1.2$ µm for their design. We averaged the reflectivity information provided in Figure 2(c) of their paper [48] to find the mean laser band reflectivity $\overline{\varrho_\perp}$ (Equation 78), and used Equations 80 and 81 with spectroscopic data from Kischkat *et al.* [7] and Munkhbat *et al.* [4] for Si_3N_4 and MoS_2, respectively, to estimate the average absorptivity. We accounted for the holes in the sail film in Equation 80 using fill factor

$$F_i = \frac{a_x a_y - 2\pi \left(\frac{d_{hole}}{2}\right)^2}{a_x a_y}, \tag{122}$$

where the factor of two in front of π is included because each unit cell in their design contained the equivalent of two holes. We obtained the effective emissivity as in Supplementary Note 14.2 using spectroscopic data from Kischkat *et al.* [7] and Munkhbat *et al.* [4] for Si_3N_4 and MoS_2, respectively.

We derived the sail's membrane mechanical robustness using Equation 113 with

$$G_i = \frac{a_y - d_{hole}}{a_y} \tag{123}$$

(see Equation 115) and yield stress values $\sigma_{y,\mathrm{Si_3N_4}} = 14$ GPa [37] and $\sigma_{y,\mathrm{MoS_2}} = 0.75$ GPa [21, 22]. We calculated the areal density of the film using the ratio of the unit cell mass to the unit cell planar area, *i.e.*,

$$\rho_a = \frac{M_{uc}}{A_{uc}}, \tag{124}$$

wherein

$$M_{uc} = \left(\rho_{\mathrm{Si_3N_4}} \left(t_{\mathrm{Si_3N_4},t} + t_{\mathrm{Si_3N_4},b}\right) + \rho_{\mathrm{MoS_2}} t_{\mathrm{MoS_2}}\right) \times \left(a_x a_y - 2\pi \left(\frac{d_{hole}}{2}\right)^2\right) \tag{125}$$

and

$$A_{uc} = a_y a_x. \tag{126}$$

Here, $\rho_{\mathrm{Si_3N_4}} = 3200\ \mathrm{kg \cdot m^{-3}}$ [36] and $\rho_{\mathrm{MoS_2}} = 5060\ \mathrm{kg \cdot m^{-3}}$ [21]. We computed the sail's maximum relative velocity using

the method outlined in Supplementary Note 12 with the reflectivity profile shown in Figure 2(c) of their paper [48]. In this simulation, we reduced the laser power because the mechanical robustness of the sail was low. Finally, we estimated the thermally-limited power $\Phi_{s,max}$ as in Supplementary Note 14.2, using $T_{max} = 1000$ K (limited by MoS_2 [23]).

This film featured a high reflectivity of $\overline{\varrho_\perp} = 0.81$, the lowest average absorptivity among films compared here ($\overline{\alpha_\perp} = 0.000041$), and also the lowest areal density ($\rho_a = 0.13\ \mathrm{g \cdot m^{-2}}$). However, since its membrane mechanical robustness was low ($\wp = 21\ \mathrm{N \cdot m^{-1}}$), it was able to tolerate a laser power of only $\Phi_l = 18$ GW, constraining its maximum relative velocity to just $\beta_{max} = 0.18$. Future work should address its mechanical shortcomings while further improving its optical properties.

14.5. Lien et al. (2022) [49]

Lien *et al.* [49] fabricated a silicon nitride photonic crystal film by patterning a $t_{\mathrm{Si_3N_4}} = 690$ nm thick Si_3N_4 layer with $d_{hole} = 415$ nm holes in a square grid with spacing $a = 1064$ nm. They optimized their design for a laser wavelength of $\lambda_l = 1.064$ µm and measured the transmissivity of their prototype; assuming negligible absorption, they estimated the reflectivity by subtracting the transmissivity from unity (see Figure 2(c) of their paper [49]). We used this definition of reflectivity to estimate the average laser band reflectivity $\overline{\varrho_\perp}$ for their design (Equation 78).

We used optical data from Kischkat *et al.* [7] in Equations 80 and 81 to calculate the average laser band absorptivity, and used the method of Supplementary Note 14.2 to obtain the effective emissivity. We calculated the appropriate fill factor for the absorptivity calculation using

$$F_i = \frac{a_x a_y - \pi \left(\frac{d_{hole}}{2}\right)^2}{a_x a_y}, \tag{127}$$

where unlike Equation 122, the pattern in the design contained only one hole per unit cell.

We derived the sail's membrane mechanical robustness using

$$G_i = \frac{a - d_{hole}}{a} \tag{128}$$

(see Equation 115; for this single-layer film $i = 1$ only) and yield stress $\sigma_{y,\mathrm{Si_3N_4}} = 14$ GPa [37] in Equation 113. We calculated the areal density using Equation 124, with

$$M_{uc} = \rho_{\mathrm{Si_3N_4}} t_{\mathrm{Si_3N_4}} \left(a^2 - \pi \left(\frac{d_{hole}}{2} \right)^2 \right) \tag{129}$$

and

$$A_{uc} = a^2. \tag{130}$$

Here $\rho_{\mathrm{Si_3N_4}} = 3200\ \mathrm{kg \cdot m^{-3}}$ [36]. We computed the sail's maximum relative velocity using the method outlined in Supplementary Note 12 with the reflectivity profile reported in Figure 2(c) of their paper [49]. Finally, we estimated the thermally-limited power $\Phi_{s,max}$ as in Supplementary Note 14.2, using $T_{max} = 1670$ K (limited by Si_3N_4 [38]).

This sail design was one of the first with an experimentally-measured reflectivity ($\overline{\varrho_\perp} = 0.44$). It is important to note that, among sail designs compared in this article, all of the experimental reflectivity values reported [49, 52, 53] are smaller than those for the theoretical designs [46–48, 50, 51, 54], suggesting that fabrication methods have room for future improvement to attain the ideal metrics. This sail had the highest membrane mechanical robustness of the group of films compared in this article ($\wp = 5892\ \mathrm{N \cdot m^{-1}}$) and also featured a relatively high emissivity-to-absorptivity ratio of $\frac{\varepsilon_e}{\overline{\alpha_\perp}} = 823$. This ratio, together with the high thermal limit of Si_3N_4, allowed the film to tolerate a relatively high thermally limited power of $\Phi_{s,max} = 348$ MW. However, this sail design had the highest areal density ($\rho_a = 1.94\ \mathrm{g \cdot m^{-2}}$), leading to a low mass-constrained area ($A_\perp = 0.48\ \mathrm{m^2}$) and a short maximum acceleration length ($L_{max} = 11.0$ Gm). Ultimately, the relatively low reflectivity of the film constrained its maximum relative velocity to $\beta_{max} = 0.17$. Future work could consider the tradeoff between mechanical robustness and reflectivity in order to optimize the design.

14.6. Santi et al. (2022) [50]

Santi *et al.* [50] numerically investigated a family of light sails composed of one, two, three, or four layers of various materials, with a laser output wavelength of $\lambda_l = 1.064$ µm.. Their optimization resulted in a three-layer TiO_2-SiO_2-TiO_2 composite film with thicknesses $t_{\mathrm{TiO_2},t} = 121$ nm, $t_{\mathrm{SiO_2}} = 203$ nm, and $t_{\mathrm{TiO_2},b} = 121$ nm. We used these layer thicknesses and index of refraction information for TiO_2 and SiO_2 from Kischkat *et al.* [7] and Rodriguez-de Marcos *et al.* [128], respectively, to calculate the wavelength-dependent reflectivity for this design using the transfer-matrix method (Supplementary Note 6), which we then averaged to determine the laser band average reflectivity $\overline{\varrho_\perp}$ (Equation 78). We also used this optical data in Equations Equations 80 and 81 with $F_i = 1$ to obtain the laser-band average absorptivity $\overline{\alpha_\perp}$. We used optical data from Kischkat *et al.* [7] for both TiO_2 and SiO_2 to estimate the effective emissivity of the film, as in Supplementary Note 14.2.

We obtained the film's membrane mechanical robustness using Equation 113 with yield stresses $\sigma_{y,\mathrm{TiO_2}} = 1.1$ GPa [43, 44] and $\sigma_{y,\mathrm{SiO_2}} = 1.5$ GPa [37, 40]. We derived the areal density *via* Equation 120, with $\rho_{\mathrm{TiO_2}} = 3700\ \mathrm{kg \cdot m^{-3}}$ [42] and $\rho_{\mathrm{SiO_2}} = 2030\ \mathrm{kg \cdot m^{-3}}$ [39]. Note that the areal density we calculated is slightly lower than that listed in their paper [50] because we used different material density values. We computed the sail's maximum relative velocity using the method outlined in Supplementary Note 12 with the reflectivity profile that we calculated above. Finally, we estimated the thermally-limited power $\Phi_{s,max}$ as in Supplementary Note 14.2, using $T_{max} = 670$ K (limited by TiO_2 [45]).

This design featured a high reflectivity ($\overline{\varrho_\perp} = 0.78$), had a high membrane mechanical robustness ($\wp = 571\ \mathrm{N \cdot m^{-1}}$), and achieved a strong maximum relative velocity ($\beta_{max} = 0.23$). However, the design had a relatively high areal density ($\rho_a = 1.31\ \mathrm{g \cdot m^{-2}}$, which caused the sail area to be only $A_\perp = 0.71\ \mathrm{m^2}$ within the mass

constraint, and thereby increased the incident light intensity focused upon it. This light intensity, combined with the low thermal limit of TiO_2, constrained the thermally limited power to only $\Phi_{s,max} = 0.26$ MW. Future designs could consider materials that could tolerate higher temperatures and absorb less light.

14.7. Taghavi and Mosallaei (2022) [51]

The paper by Taghavi and Mosallaei [51] proposed a similar design and methodology to that of Salary and Mosallaei [47] (see Supplementary Note 14.3), maintaining a laser output wavelength of $\lambda_l = 1.3$ μm. We averaged the reflectivity information provided in Figure 2(b) of their paper [51] at a constant disk diameter of 300 nm to obtain the laser band average reflectivity $\overline{\varrho_\perp}$ (Equation 78). We estimated the absorptivity using Equations 80 and 81 with optical data from Poruba *et al.* [126] and Rodriguez-de Marcos *et al.* [128] for Si and SiO_2, respectively, with a disk fill factor calculated using Equation 121 and $F_i = 1$ for the continuous 103-nm-thick Si and 60-nm-thick SiO_2 layers underneath. We obtained the sail's emissivity as in Supplementary Note 14.2, using optical information from Franta *et al.* [127] and Kischkat *et al.* [7] for Si and SiO_2, respectively.

We calculated the membrane mechanical robustness using Equation 113 with $G_i = 1$, accounting only for the strength of the backbone Si-SiO_2 film (not the Si disks), with yield stress values $\sigma_{y,\mathrm{Si}} = 2$ GPa [31–34] and $\sigma_{y,\mathrm{SiO_2}} = 1.5$ GPa [37, 40]. We obtained the sail's areal density using the ratio of the total sail mass ($m_s = 4.7$ g) to the sail area ($A_s = 4$ m^2; see Equation 116). We calculated the sail's maximum relative velocity according to the method outlined in Supplementary Note 12 using the reflectivity profile that we obtained from Figure 2(b) of their paper [51], as discussed above. Lastly, we estimated the thermally-limited power $\Phi_{s,max}$ as in Supplementary Note 14.2, using $T_{max} = 1450$ K (limited by SiO_2 [41]).

This sail design achieved similar optical properties to that proposed by Salary and Mosallaei [47]. However, its areal density was higher ($\rho_a = 1.18$ g·m^{-2}), which constrained the sail size ($A_\perp = 0.79$ m^2) within the mass budget. Since the photon-induced tension Υ is inversely proportional to the sail diameter d_s (Equation 119), the reduction in sail area increased the force on the sail to the point where the applied laser power had to be limited to $\Phi_l = 79$ GW to avoid tears. This reduction in power limited the sail's maximum relative velocity to $\beta_{max} = 0.19$. In addition, the smaller sail size increased the focused light intensity on the sail, which ultimately reduced its thermally limited power budget ($\Phi_{s,max} = 4.9$ MW).

14.8. Chang et al. (2024) [52]

Chang *et al.* [52] fabricated a bilayer Si_3N_4-Si sail with thicknesses $t_{\mathrm{Si_3N_4}} = 400$ nm and $t_{\mathrm{Si}} = 321$ nm. They patterned the Si_3N_4 layer with a square grid of holes ($d_{hole} = 415$ nm and $a = 1064$ nm) but left the Si intact. They measured both the reflectivity ϱ_λ and the transmissivity τ_λ of their prototype near their proposed laser wavelength of $\lambda_l = 1.3$ μm, and corrected their reflectivity for system losses using a gold mirror standard. We estimated the absorptivity spectrum of their prototype by subtracting their corrected reflectivity (labeled "Cali. refl.: SiN PhC+Si" in Figure 4 of their article [52]) and transmissivity (labeled "Meas. trans.: SiN PhC+Si" in Figure 4 of their article [52]) spectra from unity, and subsequently calculated the laser band reflectivity $\overline{\varrho_\perp}$ and absorptivity $\overline{\alpha_\perp}$ (Equations 78 and 81, respectively). Importantly, the optical measurements provided in the article span the wavelength range $\lambda = \langle 1.309, 1.550\rangle$ μm, whereas the laser band required to reach $\beta = 0.2$ for the stated laser output wavelength is $\lambda = \langle 1.300, 1.592\rangle$ μm. To achieve the full range in order to obtain $\overline{\varrho_\perp}$ and $\overline{\alpha_\perp}$ and calculate β_{max}, we linearly extrapolated the provided reflectivity and transmissivity data in the unknown regions, which we believe to be reasonable given the trends shown in both their experimental and simulated results. We derived the effective emissivity using the process outlined in Supplementary Note 14.2, with optical data from Franta *et al.* [127] and Kischkat *et al.* [7] for Si and Si_3N_4, respectively. We calculated the membrane mechanical robustness using Equation 113 with G_i given by Equation 128 for the ($i = 1$) Si_3N_4 layer and $G_i = 1$ for the ($i = 2$) Si layer, with $\sigma_{y,\mathrm{Si_3N_4}} = 14$ GPa [37] and $\sigma_{y,\mathrm{Si}} = 2$ GPa [31–34]. We derived the areal density using Equation 124 with

$$M_{uc} = \left(\rho_{\mathrm{Si_3N_4}} t_{\mathrm{Si_3N_4}}\right)\left(a^2 - \pi\left(\frac{d_{hole}}{2}\right)^2\right) + \rho_{\mathrm{Si}} t_{\mathrm{Si}} a^2 \qquad (131)$$

and A_{uc} from Equation 130, with $\rho_{\mathrm{Si_3N_4}} = 3200$ kg·m^{-3} [36] and $\rho_{\mathrm{Si}} = 2330$ kg·m^{-3} [30]. We calculated the sail's maximum relative velocity using the method outlined in Supplementary Note 12 using the study's measured reflectivity data. Lastly, we estimated the thermally-limited power $\Phi_{s,max}$ as in Supplementary Note 14.2, using $T_{max} = 1470$ K (limited by Si [35]).

This design was one of the first for which both the reflectivity and absorptivity were experimentally quantified. Importantly, the measured reflectivity ($\overline{\varrho_\perp} = 0.66$) was the highest among the four experimental values included in this article. Although the laser band absorptivity was relatively large ($\overline{\alpha_\perp} = 0.076$), it showed the potential of their choice of sail materials to be minimally absorbing if fabricated to the best available standards. The design had a large mechanical robustness ($\wp = 2353$ N·m^{-1}), but also had a high areal density ($\rho_a = 1.54$ g·m^{-2}). This areal density led to a low mass-constrained sail area ($A_\perp = 0.60$ m^2) and thus a very short maximum acceleration length ($L_{max} = 10.1$ Gm). Ultimately, the sail's relatively low reflectivity limited its maximum relative velocity to $\beta_{max} = 0.19$.

14.9. Norder et al. (2025) [53]

Norder *et al.* [53] fabricated a single-layer Si_3N_4 photonic crystal reflector film with thickness $t_{\mathrm{Si_3N_4}} = 200$ nm and a pentagonal hole pattern, and adopted a laser output wavelength of $\lambda_l = 1.55$ μm. They measured the reflectivity ϱ_λ in the wavelength range $\lambda = \langle 1.530, 1.620\rangle$ μm (see Figure 6(c) of their paper [53]). Because this does not include the entire Doppler-shifted wavelength range corresponding to accelerating to $\beta = 0.2$ (*i.e.*, $\lambda = \langle 1.550, 1.898\rangle$ μm), we augmented their experimental reflectance data with simulated reflectivity data from Figure 4(a) in

their manuscript [53] (labeled "Pent. opt. for T") to achieve the full required range. We averaged this data to obtain $\overline{\varrho_\perp}$ (Equation 78). We estimated the absorptivity using Equations 80 and 81 with optical data from Kischkat *et al.* [7] and $F_i = 0.71$ (derived from a micrograph of the hole pattern of their film in Figure 5(c) in their paper [53]). We used the method of Supplementary Note 14.2 to obtain the effective emissivity. To estimate the membrane mechanical robustness, we used Equation 113 with $G_i = 0.45$ (derived from a micrograph of the hole pattern of their film) along with $\sigma_{y,\mathrm{Si_3N_4}} = 14$ GPa [37]. We calculated the areal density ρ_a using

$$\rho_a = \rho_{\mathrm{Si_3N_4}} t_{\mathrm{Si_3N_4}} F_i \tag{132}$$

with $\rho_{\mathrm{Si_3N_4}} = 3200\ \mathrm{kg \cdot m^{-3}}$ [36] and $F_i = 0.71$ as above. We computed the sail's maximum relative velocity using the method outlined in Supplementary Note 12 with the reflectivity profile explained above. Finally, we estimated the thermally-limited power $\Phi_{s,max}$ as in Supplementary Note 14.2, using $T_{max} = 1670$ K (limited by Si_3N_4 [38]).

Impressively, this paper presented the largest experimental sail film prototype to date ($60 \times 60\ \mathrm{mm}^2$), suggesting the potential of producing even larger high-quality light sail films in the future. This sail featured the highest emissivity-to-absorptivity ratio of those considered in this article ($\frac{\varepsilon_e}{\overline{\alpha_\perp}} = 1259$), and thus was the only film with the potential to tolerate more than a gigawatt of laser power within its thermal limit ($\Phi_{s,max} = 1369$ MW). In addition, it had a low areal density ($\rho_a = 0.45\ \mathrm{g{\cdot}m^{-2}}$) and a high membrane mechanical robustness ($\wp = 1260\ \mathrm{N \cdot m^{-1}}$). However, like the other experimental prototypes considered, its reflectivity was relatively low ($\overline{\varrho_\perp} = 0.38$), limiting it to a maximum relative velocity of $\beta_{max} = 0.18$.

14.10. Whittam et al. (2025) [54]

Whittam *et al.* [54] proposed a Si/SiO_2 core shell nanosphere design with the spheres held together by a polydimethylsiloxane (PDMS) embedding. They specified a laser output wavelength of $\lambda_l = 1.0$ µm. We averaged the reflectivity provided in Figure 9(d) of their manuscript [54] to obtain the laser band average reflectivity $\overline{\varrho_\perp}$ (Equation 78). We estimated the film's absorptivity using Equations 80 and 81 by deriving equivalent film thicknesses $t_{eq,i}$ for the three materials based on their volume fractions f_i, as outlined below. In this calculation, we used optical data from Poruba *et al.* [126] (Si), Rodriguez-de Marcos *et al.* [128] (SiO_2), and Zhang *et al.* [147, 148] (PDMS, mass main component to mass curing agent in ratio 5:1).

$$t_f = 3r_2 \tag{133}$$

$$r_1 = 0.99r_2 \tag{134}$$

$$\Lambda = 2.5r_2 \tag{135}$$

$$V_{uc} = t_f \Lambda^2 \tag{136}$$

$$f_{\mathrm{Si}} = \frac{\frac{4}{3}\pi r_1^3}{V_{uc}} \tag{137}$$

$$f_{\mathrm{SiO_2}} = \frac{\frac{4}{3}\pi \left(r_2^3 - r_1^3\right)}{V_{uc}} \tag{138}$$

$$f_{\mathrm{PDMS}} = 1 - f_{\mathrm{Si}} - f_{\mathrm{SiO_2}} \tag{139}$$

$$t_{eq,i} = f_i t_f \tag{140}$$

Here t_f, r_1, r_2, Λ, and V_{uc} are the PDMS thickness, the core Si sphere radius, the shell SiO_2 outer radius, the center-to-center sphere spacing (square grid), and the unit cell volume. In their design, $r_2 = 180.8$ nm. We obtained the sail's emissivity as in Supplementary Note 14.2, accounting only for the emissivity of the PDMS [147, 148]. This simplification is reasonable given that the extinction coefficient of Si is about an order of magnitude lower than that of PDMS in the wavelength range of interest ($\lambda = \langle 2, 14 \rangle$ µm) [127], and that the volumetric content of SiO_2 in the design is less than a percent. We estimated the membrane mechanical robustness by accounting for the strength of the PDMS embedding using Equation 113 with $G_i = 0.6$, obtained as the ratio of the minimum distance between spheres $\Lambda - r_2$ to the sphere center-to-center spacing Λ (see Equation 135). In this calculation we used a tensile yield strength for PDMS of $\sigma_{y,PDMS} = 0.005$ GPa [28]. We calculated their film's areal density using Equation 124 with

$$M_{uc} = V_{uc} \sum_{i=1}^{n} f_i \rho_i \tag{141}$$

and

$$A_{uc} = \Lambda^2 \tag{142}$$

using densities $\rho_{\mathrm{Si}} = 2330\ \mathrm{kg{\cdot}m^{-3}}$ [30], $\rho_{\mathrm{SiO_2}} = 2030\ \mathrm{kg{\cdot}m^{-3}}$ [39], and $\rho_{PDMS} = 980\ \mathrm{kg \cdot m^{-3}}$ [27]. We calculated the sail's maximum relative velocity according to the method outlined in Supplementary Note 12 using the reflectivity profile that we obtained from their paper [54], as discussed above. Lastly, we estimated the thermally-limited power $\Phi_{s,max}$ as in Supplementary Note 14.2, using $T_{max} = 620$ K (limited by PDMS [29]).

This design was noteworthy because it achieved the highest reflectivity among the films examined in this article ($\overline{\varrho_\perp} = 0.89$). However, its membrane mechanical robustness was low ($\wp = 1.63\ \mathrm{N \cdot m^{-1}}$) because of the low tensile yield strength of PDMS, which limited the laser power that could be applied to only $\Phi_l = 0.51$ GW, which in turn constrained the sail's maximum relative velocity to just $\beta_{max} = 0.026$. This design could be improved if a stronger and low-absorbing webbing material for connecting the nanospheres were to be identified.

14.11. Fabricated prototype film (this study)

The fabricated prototype film characterized in Figure 3(c) of the main article consists of a Al_2O_3-MoS_2-Al_2O_3 composite ($t_{A,b} \approx 21$ nm (bottom Al_2O_3 thickness), $t_M \approx 53$ nm (MoS_2), $t_{A,t} \approx 51$ nm (top Al_2O_3)) with a hexagonally corrugated indented trench structure ($d_h \approx 77$ µm, $w_t \approx 15$ µm, $h_t \approx 10$ µm; see Supplementary Figure 12). We averaged our measured reflectivity and absorptivity data ($\alpha_{\lambda,\perp} = 1 - \varrho_{\lambda,\perp} - \tau_{\lambda,\perp}$) to obtain the laser band average values ($\beta = 0$ to $\beta = 0.2$, or equivalently, for our

laser wavelength, the range $\lambda = \langle 1.2, 1.4697\rangle$ µm) for reflectivity $\overline{\varrho_\perp}$ and absorptivity $\overline{\alpha_\perp}$ (Equations 78 and 81, respectively). Note that, especially in the case of curved sails [87], some light will encounter the film at off-normal angles. To estimate the change in optical properties that this could entail, we simulated the reflectivity and absorptivity of the fabricated prototype sail film using our measured indices of refraction (see Supplementary Note 8). The results, shown in Supplementary Figure 27, suggest that the angular dependence of the sail's reflectivity and absorptivity is only moderate; within the laser band ($\lambda = \langle 1.2, 1.4697\rangle$ µm), the reflectivity and absorptivity change by up to 9% and 2.5%, respectively, within a 0-to-50° angular range. We used spectroscopic information from Kischkat *et al.* [7] for Al_2O_3 and a two-term Cauchy expansion of our experimentally-measured MoS_2 index of refraction (see Supplementary Figure 19) to estimate the film's effective emissivity, as in Supplementary Note 14.2. For reference, our calculations of the film's spectral hemispherical emissivity (Equation 82) are provided in Supplementary Figure 28. We calculated the membrane mechanical robustness of our design using Equation 113 with $G_i = 1$ and yield stress values $\sigma_{y,\mathrm{Al_2O_3}} = 2$ GPa [16, 17] and $\sigma_{y,\mathrm{MoS_2}} = 0.75$ GPa [21, 22]. Our choice of $G = 1$ is conservative and allows us to examine the robustness of the film in a planar (non-corrugated) state. However, the hexagonal structure can actually be designed to increase compliance to in-plane stresses, thereby increasing the practical membrane mechanical robustness [80]. We derived the areal density using Equations 3, 5, and 7, with $\rho_A = 3200\ \mathrm{kg\cdot m^{-3}}$ [15] and $\rho_M = 5060\ \mathrm{kg\cdot m^{-3}}$ [21]. We calculated the film's maximum relative velocity using the method outlined in Supplementary Note 12 using our measured reflectivity data. Finally, we estimated the thermally-limited power $\Phi_{s,max}$ as in Supplementary Note 14.2, using $T_{max} = 1000$ K (limited by MoS_2 [23]).

14.12. Optimized film (this study)

The optimized film that we propose consists of a Al_2O_3-MoS_2-Al_2O_3 composite ($t_{A,b} \approx 19$ nm (bottom Al_2O_3 thickness), $t_M \approx 63$ nm (MoS_2), $t_{A,t} \approx 19$ nm (top Al_2O_3)) with a hexagonally corrugated indented trench structure ($d_h = 70$ µm, $w_t = 2$ µm, $h_t = 3$ µm; see Supplementary Figure 12). We used index of refraction information for Al_2O_3 from Kischkat *et al.* [7] (linearly extrapolating from $\lambda = 1.53941$) and for MoS_2 from Munkhbat *et al.* [4] to determine the laser band reflectivity spectrum according to the transfer-matrix method, and averaged it to determine $\overline{\varrho_\perp}$ (Equation 78). We also used these optical constants to derive the absorptivity using Equations 80 and 81, and in addition to obtain the effective emissivity using the method of Supplementary Note 14.2. For reference, our calculations of the film's spectral hemispherical emissivity (Equation 82) are provided in Supplementary Figure 28. In addition, for reference and completeness, we calculated the angular dependence of the reflectivity and absorptivity of our proposed optimized sail film using the optical data listed above [4, 7]; the results are provided in Supplementary Figure 29.

We calculated the membrane mechanical robustness using $G_i = 1$ and tensile yield stress values $\sigma_{y,\mathrm{Al_2O_3}} = 2$ GPa [16, 17] and $\sigma_{y,\mathrm{MoS_2}} = 2.3$ GPa [13] (see Equation 113 and Sections 4 and 13), and the areal density using $\rho_A = 3200\ \mathrm{kg\cdot m^{-3}}$ [15] and $\rho_M = 5060\ \mathrm{kg\cdot m^{-3}}$ [21]. We calculated the maximum relative velocity as outlined in Supplementary Note 12 using the reflectivity data that we calculated above. Finally, we estimated the thermally-limited power $\Phi_{s,max}$ as in Supplementary Note 14.2, using $T_{max} = 1000$ K (limited by MoS_2 [23]).

Supplementary Note 15: Energy analysis of accelerating sail

Here we describe our calculation of the equilibrium temperature during the acceleration of our optimized design. We performed this simulation with similar constraints to the others in this work: a sail mass of $m_s = 1$ g, a total sailcraft mass (sail, chip, and tethers) of $m_{tot} = 2$ g, a circular sail with a spherical radius of curvature equal to its diameter ($d_s = s_s$), and a maximum constant laser output power of $\Phi_l = 100$ GW (uniform/flat-top intensity distribution). Our approach was to divide the acceleration interval ($\beta = 0$ to $\beta = \beta_f = 0.2$) into discrete β steps and solve Equation 46 to determine the sail temperature at each relative velocity value. We also checked the mechanical integrity of the sail (photon-induced tension) during its acceleration, using the same material properties as in Supplementary Note 13.

For the optical properties of MoS_2 we used the data of Munkhbat *et al.* [4], extrapolating the n values using a Cauchy expansion and setting the κ values equal to 10^{-8} at wavelengths equal to and longer than the laser wavelength of $\lambda_l = 1.2$ µm. For the n optical coefficients of Al_2O_3 we used the data of Querry [6] at all wavelengths. For the κ data of Al_2O_3, at wavelengths equal to and shorter than $\lambda_A = 7$ µm we used the values of Lingart, Petrov, and Tikhonova [5]; at wavelengths equal to and greater than $\lambda_B = 10$ µm we used the values of Querry [6]; and in between we used a power-law interpolation of the form

$$\kappa = a\lambda^b \tag{143}$$

where

$$b = \frac{\ln\left(\frac{\kappa_A}{\kappa_B}\right)}{\ln\left(\frac{\lambda_A}{\lambda_B}\right)} \tag{144}$$

and

$$a = \frac{\kappa_A}{\lambda_A^b}. \tag{145}$$

Here, κ_A and κ_B correspond to the extinction coefficients provided by Lingart, Petrov, and Tikhonova [5] and Querry [6] at λ_A and λ_B, respectively. We used these optical coefficients and the transfer-matrix method to calculate the reflectivity and absorptivity at each β-step throughout the acceleration, and also used the transfer-matrix method to obtain the spectral hemispherical emissivity in the wavelength range $\lambda = \langle 0.301, 20\rangle$ µm (Equation 82). Also, in order to provide a more realistic portrait of the sail's accelerative performance, we accounted for the impact of the spherically-curved sail's shape on its reflectivity using Equation 76, which required calculating angle-dependent reflectivity values.

The results of our calculations are provided in Supplementary Figure 30. Panel (h) shows that the peak temperature occurred at the beginning of the acceleration phase, and that, even with a laser output power of $\Phi_l = 100$ GW, the sail stayed cooler than than the MoS_2 vacuum sublimation point of about 1000 K [23]. This result is predicated on our selection of $\kappa = 10^{-8}$ for MoS_2 in the laser band; higher extinction coefficients would require reducing the laser output power to avoid overheating the sail. In this particular simulation, the constraining parameter for this design was the photon-induced tension (panel (g)), which was the factor for which we optimized this design (see Supplementary Note 13).

A few more aspects of these acceleration calculations are worth discussing. Though we held the laser output power, shown in Supplementary Figure 30(c), constant, the power incident on the sail decreased with time due to the red shift of the photons and the reduced photon flux relative to the accelerating sail. Notice that the time duration during which the laser needed to produce photons, $t_l = 5.0$ min, was less than the sail's acceleration time, $t_a = 5.6$ min, because the last photon leaving the laser took about 0.6 min to reach the sail. The acceleration experienced by the sail (in its own reference frame) was also higher than that in the laser's reference frame due to the relativistic increase in the sailcraft's mass from the laser's perspective (panel (e)). The average reflectivity of the spherically curved sail (panel (i)) was smaller than that of the perpendicular component (at the sail center); both decreased with time as the laser wavelength reaching the sail (panel (b)) was redshifted. Ultimately, our calculations indicated that our proposed optimized film could accelerate from rest to $\beta = 0.2$ over a distance of $L \approx 11$ Gm, and would require a laser array diameter on Earth of $d_{l,E} \approx 17$ km.

Supplementary Note 16: Achieving enhanced optical properties

Achieving optically perfect films will be facilitated by continued advances in fabrication techniques [149, 150]. At the present time, we see several routes by which improved film quality and performance could be achieved.

A principal strategy to minimize the absorptivity of alumina would be to anneal the as-deposited amorphous form into a crystalline state. We assumed such a pathway in using the extinction coefficients from Lingart, Petrov, and Tikhonova [5] in Supplementary Note 15. Recent work has shown that annealing ALD Al_2O_3 films for just 30 min in a H_2 or N_2 atmosphere at a pressure of 10^5 Pa and a temperature of around 1000 K is sufficient to accomplish this [151, 152] (but note that another study suggested a temperature of around 1400 K [153]). (We also note that, while annealing greatly reduces the extinction coefficient of Al_2O_3, it has only a small impact its real component of the index of refraction [154].) Annealing the alumina may impact its mechanical properties; the yield stress of bulk sapphire is roughly an order of magnitude lower than that for thin ALD alumina films. We are unaware of studies on the mechanical properties of thin sapphire films, but there is some evidence that annealing Al_2O_3 films at temperatures up to 1000 K does not adversely impact them [155]. Annealing of the face alumina films would need to take place in the presence of the inner MoS_2 film. As we noted earlier, MoS_2 is known to sublimate in vacuum environments at temperatures exceeding 1000 K. However, recent work suggests that it can be heated to 1500 K without evaporating if it is pressurized to > 100 Pa (gaseous environment) [23]. Even if sublimation were to occur, it may be relatively slow; a study by Lu *et al.* found that at 923 K and 1300 Pa it took 1 h to remove one layer (about 0.65 nm) of a MoS_2 flake [156].

To minimize the absorptivity of the MoS_2, other emerging fabrication strategies [157, 158], including atomic layer deposition [159] or sputtering [160], could be employed. MoS_2 films of sufficient quality can in principle have very low absorptivity in the laser band ($\lambda = \langle 1.2, 1.4697 \rangle$ µm) because the photon energy ($E = \langle 1.03, 0.84 \rangle$ eV) is less than the material's bandgap (1.3-1.8 eV) [161]. For instance, Ermolaev *et al.* [2], Hsu *et al.* [106], and Liu *et al.* [10] reported optical coefficients of $\kappa = 0$ for MoS_2 at or near $\lambda = 1.2$ µm. Thus, our use of an extinction coefficient of $\kappa = 10^{-8}$ for MoS_2 in Supplementary Note 15 is optimistic, but not unreasonable.

There are also several routes to increase the emissivity of the film at longer wavelengths for enhanced radiative cooling. For instance, patterning micron-scale features (larger than the longest wavelength in the laser band) can excite surface phonon polariton resonances and increase mid-infrared extinction coefficients without significantly impacting the laser band absorbance. Alternatively, other materials with high mid-infrared extinction coefficients, such as SiO_2 [162], Si_3N_4 [7, 48], or (Al:)ZnO [163], could be used.

Together, these factors suggest that future Al_2O_3-MoS_2-Al_2O_3 light sails could feature alumina and MoS_2 extinction coefficients as low as or even lower than $\kappa = 10^{-7}$ and $\kappa = 10^{-8}$, respectively. This also holds promise that next-generation sails will have sufficient mid-infrared emissivity as to allow emissivity-to-absorptivity ratios of $\frac{\varepsilon_e}{\alpha_\perp} > 10^6$, sufficient for radiative cooling to tolerable temperatures.

Supplementary Note 17: Impact of temperature

Elevated temperatures will impact the mechanical and optical properties of the sail. While a dedicated study of these effects for thin films will ultimately be required (but is now beyond the scope of this work), we can make some observations from related studies in the literature [84, 164].

The mechanical properties of Al_2O_3 and MoS_2 will degrade with temperature. The fracture stress of sapphire has been found to decrease by roughly 15% from room temperature to 1000 K [165], and the yield stress of polycrystalline alumina was found to decrease by as much as 62% over the same range [166]. Likewise, molecular dynamics simulations of monolayer MoS_2 flakes suggest that their ultimate stress decreases by roughly 23% from room temperature to 600 K [167]. This degradation could be offset by using lower laser powers (decreasing the photon flux and hence the photon-induced tension) or thicker film layers (increasing the sail's membrane mechanical robustness).

The extinction coefficients for Al_2O_3 and MoS_2 will likely increase with temperature; unfortunately, high-temperature optical data within the laser band for these materials is scarce. Lingart, Petrov, and Tikhonova [5] compiled information for sapphire at elevated temperatures, which suggests that its extinction coefficient in the laser band roughly triples (190% increase) from room temperature to 1000 K (Supplementary Figure 31). However, this is accompanied by a similar increase at longer wavelengths, suggesting that the sail will be able to emit more radiation to slightly offset the increased photon absorption. The real component of the index of refraction of sapphire increases negligibly (1%) over the same temperature range. Data reported by Liu *et al.* for MoS_2 suggest that its extinction coefficient in the laser band does not change significantly from 4.5 K to 300 K, and that it may decrease at higher temperatures [10] (Supplementary Figure 32). Also, according to their results, the real component of the index of refraction of MoS_2 changes by less than 1% from room temperature to 500 K.

As the sail heats it will be impacted by thermal expansion. As shown in Supplementary Figure 33, Al_2O_3 and MoS_2 have well-matched thermal expansion coefficients, α_A and α_M, respectively - within about 5% in the range 500-800 K and within 10-15% up to 1000 K [11, 12, 168, 169]. We can estimate the strain ϵ_T that this thermal expansion induces using

$$\epsilon_T = (T - T_0)\,|\alpha_A - \alpha_M| \tag{146}$$

where T is the temperature of interest and T_0 is a reference temperature at which the strain is negligible. This equation indicates that the thermal strain will be less than a tenth of a percent from room temperature up to 1000 K, and therefore suggests that thermal strain is not likely to be a failure mode for the sail.

Finally, we note that several studies have observed that defects at the interfaces between thin film layers can lead to increased absorption and film degradation [170–173]. Our expectation is that continuing improvements in fabrication techniques will allow large-area nanolaminate films to be formed with perfect interface matching, *i.e.*, avoiding defects, inclusions, and contamination [170]. This seems especially possible given the demonstrated compatibility between thin films of Al_2O_3 and MoS_2 (see Supplementary Note 1) [55–66]. Should interface defects be found to be problematic, we propose several courses of action. First, a single-layer film, constructed with materials such as Si_3N_4, could be formed into the same hexagonally-corrugated structure that we demonstrated, since this by definition avoids film-film interfaces [53, 173]. Second, sail materials that have high thermal conductivity (to rapidly dissipate heat generated at defects) and low coefficients of thermal expansion (to avoid thermal stress) could be selected [170]. Finally, sail designs could be conceived that avoid sub-wavelength nanophotonic structures, because the strong field localization they cause can result in increased absorption [172].

Supplementary Note 18: Photon scattering by hexagonal corrugation

The hexagonal corrugation in our sails may cause some photons to scatter rather than be reflected specularly. We can conservatively estimate the fraction of incident light that will be affected by examining Supplementary Figures 12 and 13. Each single hexagonal unit cell shown has an area given by Equations 7 or 8 for the indented trench and protruding rib configurations, respectively. Also, each unit cell comprises six hexagon sides with length a_h (Equation 1). The lateral width impacted by each vertical wall can be estimated as being roughly proportional to the depth of the trenches h_t or height of the ribs h_r. Thus, the total area impacted by scattering (henceforth written for the trench configuration) can be approximated by

$$A_{s,t} \approx 6a_h h_t \tag{147}$$

Combining Equations 1, 7, and 147, we find the fraction of impacted area $F_{scatter}$ to be approximately

$$F_{scatter} \approx \frac{A_{s,t}}{A_{uc,t}} = \frac{6\frac{d_h}{\sqrt{3}}h_t}{\frac{\sqrt{3}}{2}\left(d_h + w_t\right)^2} = \frac{4d_h h_t}{\left(d_h + w_t\right)^2}. \tag{148}$$

For the fabricated prototype design with $d_h \approx 77$ µm, $w_t \approx 15$ µm, and $h_t \approx 10$ µm, we find $F_{scatter} = 0.364$. For the proposed optimized design, with $d_h = 70$ µm, $w_t = 2$ µm, and $h_t = 3$ µm, we have $F_{scatter} = 0.162$. Note that these fractions are conservative estimates, and detailed numerical simulations would be necessary to more accurately quantify the scattering. Future work would benefit from co-optimizing the mechanical aspects of corrugated sails (bending stiffness enhancement and tensile stiffness reduction) and the optical performance. We also note that, as introduced in Supplementary Note 9 and Supplementary Figure 24, we designed films featuring a flat reflective layer on top of a corrugated backbone. Such designs may mitigate scattering.

Supplementary Note 19: Beam-riding stability

Light sails must be designed to stay within the laser beam during the acceleration phase. For instance, if the sail shifts laterally away from the center of the laser beam or tilts so its heading is no longer aligned with the beam, the sail should feature automatic compensation mechanisms to bring it back into alignment. Methods for damping oscillations are also important [174]. The stabilization must generally be passive because feedback control on the laser beam would be difficult given the fast acceleration experienced by light sails. Leading strategies for sail stabilization include selecting macroscopic shapes such as spherical curves that inherently imply stability [87, 98, 175] or by leveraging nanostructured or diffractive surfaces that scatter light in favorable ways [47, 51, 99, 100, 145, 174, 176–180]. A detailed study of these methods is beyond the scope of this work, but we briefly summarize a few considerations for corrugated light sail films in particular.

As mentioned in Supplementary Note 18, some photons may be scattered by the corrugated structure of our sail films. These scattered photons could have the potential to destabilize the sail, making it important to implement stabilizing techniques to compensate. A simple method could be to employ a spherically curved

shape for the sail [87, 98]. In this aspect, hexagonal corrugation offers the benefit of increased bending stiffness relative to flat plates, thereby helping the film to maintain its curved shape despite the radiation pressure. In addition, metamaterial coatings could be applied to the horizontal elements in our films (*i.e.*, the hexagon tops and trench bottoms for films in the indented trench configuration). Presumably, if fabricated with sufficiently high precision, the films' microfabricated hexagonal architecture would give rise to predictable or repeatable scattering patterns, and the coatings could be designed with this in mind. Such features would need to be applied in radially-varying arrays to generate the correct effects in reflectivity magnitude and phase. These features could be patterned by growing them on top of the film (additive manufacturing) or by etching them into the Si mold prior to applying the first Al_2O_3 coating. Finally, as mentioned in Supplementary Note 9 (see also Supplementary Figure 24), we have experimented with adding a flat MoS_2 film on top of a corrugated Al_2O_3 frame. The upper flat surface could serve to prevent edge scattering.

Supplementary Note 20: Robustness of light sail in space

Interstellar space is a harsh environment, and during its multi-year transit to another star system the sail film will likely encounter radiation, micro-meteoroids, reactive radicals, and other hazards [164, 181–184]. Although in principle the sail would only need to stay intact for a few minutes during its acceleration phase, it would be beneficial for it to survive the entire journey because (1) were it to disintegrate it could alter the sail's trajectory; (2) the sail could be useful for transmitting data back to Earth [179]; and (3) the sail could be used for slowing the probe as it approached a foreign star to provide more time for observation [90, 185, 186]. Several articles have discussed this problem in detail, studying the size distribution of dust particles in space [181], the potential of a foil shield to mitigate impacts from dust and gas particles [182], the impact of dust and gas bombardment on light sails [183], the impact of electromagnetic forces on light sails [184], and the potential for heating of light sails due to dust bombardment [164].

While a thorough discussion of the robustness of our class of light sails is beyond the scope of this work, we briefly offer a few thoughts here. First, as mentioned in the main article, the out-of-plane corrugated structure of our sails prevents crack propagation by deflecting cracks at the vertical trench or rib walls [74]; this may be an important factor given the inevitability of impacts from dust and gas. Second, both Al_2O_3 and MoS_2 have been proposed for use in space - the former as a coating to protect components against atomic oxygen [187] or as thermal insulation [188], and the latter as a solid-based lubricant [189, 190] or as an absorber in photovoltaic cells [191]. This suggests their viability as materials for light sails from a survivability standpoint. Third, several studies have examined the durability of these materials in space (or in vacuum on Earth) [189, 191–193] - some for equivalent durations of months or longer [188, 194, 195]. Although this is only a fraction of the required transit time to another star system, it indicates that these materials hold promise and warrant further study.

Supplementary Note 21: Scalability

Any sail film design must be scalable, in the sense that square-meter-sized areas must be fabricated or pieced together to produce a single sailcraft, and that a fleet of many sails must be built and sent toward the destination (*e.g.*, the Breakthrough Starshot Foundation's goal of Proxima Centauri [88, 92, 131, 132]) to increase the chances of a successful mission. The backside etching approach that we used to create the prototypes for optical testing would be sub-optimal for creating large area suspended films because laser-micromachining is a slow process. Here we briefly suggest three routes toward scaling up our class of films.

First, a straightforward approach would simply be to remove the silicon substrate from the front through tiny holes cut in the film. This would obviate the backside etching step; a similar strategy was employed by Norder *et al.* [53]. We envision that photolithography and ion milling could be used to drill the etch holes, and XeF_2 vapor etching could be used to remove the Si. We have used a similar process to create large Al_2O_3 films, one of which is shown in Supplementary Figure 34 [196, 197]. A potential challenge to this approach is that photolithography is limited to wafer-scale substrates, implying that many films would need to be assembled to achieve a full light sail. It would be important to ensure that all of the photoresist was removed from the film in this process, which could conceivably be accomplished using O_2 plasma ashing and verified using fluorescence microscopy or related diagnostics.

Second, another option would be to use roll-to-roll nanoimprint lithography [198–202]. In this strategy, rollers would press the out-of-plane hexagonal pattern into a sacrificial polymer film, which would serve as a substrate mold for subsequent Al_2O_3 and MoS_2 deposition steps. A MoS_2 sputtering process could be used, rather than a hotter furnace-based Mo-to-MoS_2 conversion, to prevent warping of the sacrificial polymer film [160, 203], and the sacrificial film could be removed later using O_2 plasma ashing. This process could potentially be performed on large films directly in the vacuum of low-Earth orbit [204, 205]. As in the previous option, it would be important to ensure that the entire sacrificial film was eliminated at the end of the process.

Finally, a third option would be to use deep reactive ion etching (DRIE) on the backside of the wafer to remove the substrate Si [206]. For large areas, this is a faster process than laser micromachining because it can be done simultaneously over the entire wafer area rather than through rastering. After removing a majority of the Si with DRIE, the remainder could be gently etched away using XeF_2 vapor etching. A potential bottleneck of this approach is that the DRIE process is limited to wafer-scale lateral areas. In addition, it would be important to protect the corrugated film on the front side of the wafers during the backside etching process. Whereas the wafer can be suspended in mid-air in the laser rastering method (so the corrugated film is not scratched), in DRIE the wafer must be in firm thermal contact with the tool chuck to ensure sufficient cooling. One possibility would be to sputter a temporary thin aluminum coating on the corrugated film for protection, which

could be wet-etched away after the DRIE step but before the final XeF_2 etch.

While the second scale-up pathway (roll-to-roll) could be directly done on square-meter-sized areas, the first and third pathways would require processing Si wafers. A wafer-based scale-up strategy was suggested previously by Norder *et al.* [53]. By area, it would require roughly ten 300-mm diameter wafers to achieve a 1-m diameter sail and roughly 1100 wafers for a 10-m diameter sail. This represents just a fraction of the daily output of professional foundries, suggesting the viability of this wafer-based scale-up approach. To connect several films together, the pieces could be stitched to a transparent semi-rigid frame [87, 92], likely made from a ceramic material such as Al_2O_3. Alternatively, pieces could be tiled and hooked to one another like pieces in a puzzle, potentially eliminating the need for a large frame [207]. A final option could be a form of thermal spot welding to join corners of the films together into a large array [208].

Supplementary References

[1] Song, B., Gu, H., Fang, M., Chen, X., Jiang, H., Wang, R., Zhai, T., Ho, Y.-T., Liu, S.: Layer-dependent dielectric function of wafer-scale 2D MoS_2. Advanced Optical Materials **7**(2), 1801250 (2019) https://doi.org/10.1002/adom.201801250

[2] Ermolaev, G.A., Stebunov, Y.V., Vyshnevyy, A.A., Tatarkin, D.E., Yakubovsky, D.I., Novikov, S.M., Baranov, D.G., Shegai, T., Nikitin, A.Y., Arsenin, A.V., Volkov, V.S.: Broadband optical properties of monolayer and bulk MoS_2. npj 2D Mater. Appl. **4**(1), 21 (2020) https://doi.org/10.1038/s41699-020-0155-x

[3] Islam, K.M., Synowicki, R., Ismael, T., Oguntoye, I., Grinalds, N., Escarra, M.D.: In-plane and out-of-plane optical properties of monolayer, few-layer, and thin-film MoS_2 from 190 to 1700 nm and their application in photonic device design. Adv. Photon. Res. **2**(5), 2000180 (2021) https://doi.org/10.1002/adpr.202000180

[4] Munkhbat, B., Wróbel, P., Antosiewicz, T.J., Shegai, T.O.: Optical constants of several multilayer transition metal dichalcogenides measured by spectroscopic ellipsometry in the 300–1700 nm range: High index, anisotropy, and hyperbolicity. ACS Photonics **9**(7), 2398–2407 (2022) https://doi.org/10.1021/acsphotonics.2c00433

[5] Lingart, Y.K., Petrov, V.A., Tikhonova, N.A.: Optical-properties of leucosapphire at high-temperatures. I. Translucent region. High Temp. **20**(5), 706–713 (1982). URL: http://mi.mathnet.ru/tvt6472

[6] Querry, M.R.: Optical constants. Technical Report CRDC-CR-85034, University of Missouri (June 1985). URL: https://apps.dtic.mil/sti/pdfs/ADA158623.pdf

[7] Kischkat, J., Peters, S., Gruska, B., Semtsiv, M., Chashnikova, M., Klinkmüller, M., Fedosenko, O., Machulik, S., Aleksandrova, A., Monastyrskyi, G., Flores, Y., Masselink, W.T.: Mid-infrared optical properties of thin films of aluminum oxide, titanium dioxide, silicon dioxide, aluminum nitride, and silicon nitride. Appl. Opt. **51**(28), 6789–6798 (2012) https://doi.org/10.1364/AO.51.006789

[8] Marr, J.M., Wilkin, F.P.: A better presentation of Planck's radiation law. American Journal of Physics **80**(5), 399–405 (2012) https://doi.org/10.1119/1.3696974

[9] Kumar, P., Lynch, J., Song, B., Ling, H., Barrera, F., Kisslinger, K., Zhang, H., Anantharaman, S.B., Digani, J., Zhu, H., Choudhury, T.H., McAleese, C., Wang, X., Conran, B.R., Whear, O., Motala, M.J., Snure, M., Muratore, C., Redwing, J.M., Glavin, N.R., Stach, E.A., Davoyan, A.R., Jariwala, D.: Light–matter coupling in large-area van der Waals superlattices. Nature Nanotechnology **17**(2), 182–189 (2022) https://doi.org/10.1038/s41565-021-01023-x

[10] Liu, H.-L., Yang, T., Chen, J.-H., Chen, H.-W., Guo, H., Saito, R., Li, M.-Y., Li, L.-J.: Temperature-dependent optical constants of monolayer MoS_2, $MoSe_2$, WS_2, and WSe_2: Spectroscopic ellipsometry and first-principles calculations. Sci. Rep. **10**(1), 15282 (2020) https://doi.org/10.1038/s41598-020-71808-y

[11] Hayashi, H., Watanabe, M., Inaba, H.: Measurement of thermal expansion coefficient of $LaCrO_3$. Thermochim. Acta **359**(1), 77–85 (2000) https://doi.org/10.1016/S0040-6031(00)00507-4

[12] Huang, L.F., Gong, P.L., Zeng, Z.: Correlation between structure, phonon spectra, thermal expansion, and thermomechanics of single-layer MoS_2. Phys. Rev. B **90**(4), 045409 (2014) https://doi.org/10.1103/PhysRevB.90.045409

[13] Bertolazzi, S., Brivio, J., Kis, A.: Stretching and breaking of ultrathin MoS_2. ACS Nano **5**(12), 9703–9709 (2011) https://doi.org/10.1021/nn203879f

[14] Groner, M.D., Fabreguette, F.H., Elam, J.W., George, S.M.: Low-temperature Al_2O_3 atomic layer deposition. Chemistry of Materials **16**(4), 639–645 (2004) https://doi.org/10.1021/cm0304546

[15] Ilic, B., Krylov, S., Craighead, H.G.: Young's modulus and density measurements of thin atomic layer deposited films using resonant nanomechanics. Journal of Applied Physics **108**(4), 044317 (2010) https://doi.org/10.1063/1.3474987

[16] Miller, D.C., Foster, R.R., Jen, S.-H., Bertrand, J.A., Cunningham, S.J., Morris, A.S., Lee, Y.-C., George, S.M., Dunn, M.L.: Thermo-mechanical properties of alumina films created using the atomic layer deposition technique. Sens. Actuators, A **164**(1), 58–67 (2010) https://doi.org/10.1016/j.sna.2010.09.018

[17] Jen, S.-H., Bertrand, J.A., George, S.M.: Critical tensile and compressive strains for cracking of Al_2O_3 films grown by atomic layer deposition. J. Appl. Phys. **109**(8), 084305 (2011) https://doi.org/10.1063/1.3567912

[18] Schneider, S.J., McDaniel, C.L.: Effect of environment upon the melting point of Al_2O_3. J. Res. Natl. Bur. Stand. A Phys. Chem. **71A**(4), 317–333 (1967) https://doi.org/10.6028/jres.071A.038

[19] Tripp, M.K., Stampfer, C., Miller, D.C., Helbling, T., Herrmann, C.F., Hierold, C., Gall, K., George, S.M., Bright, V.M.: The mechanical properties of atomic layer deposited alumina for use in micro- and nano-electromechanical systems. Sens. Actuators, A **130–131**, 419–429 (2006) https://doi.org/10.1016/j.sna.2006.01.029

[20] Ylivaara, O.M.E., Liu, X., Kilpi, L., Lyytinen, J., Schneider, D., Laitinen, M., Julin, J., Ali, S., Sintonen, S., Berdova, M., Haimi, E., Sajavaara, T., Ronkainen, H., Lipsanen, H., Koskinen, J., Hannula, S.-P., Puurunen, R.L.: Aluminum oxide from trimethylaluminum and water by atomic layer deposition: The temperature dependence of residual stress, elastic modulus, hardness and adhesion. Thin Solid Films **552**, 124–135 (2014) https://doi.org/10.1016/j.tsf.2013.11.112

[21] Graczykowski, B., Sledzinska, M., Placidi, M., Saleta Reig, D., Kasprzak, M., Alzina, F., Sotomayor Torres, C.M.: Elastic properties of few nanometers thick polycrystalline MoS_2 membranes: A nondestructive study. Nano Lett. **17**(12), 7647–7651 (2017) https://doi.org/10.1021/acs.nanolett.7b03669

[22] Sledzinska, M., Jumbert, G., Placidi, M., Arrighi, A., Xiao, P., Alzina, F., Sotomayor Torres, C.M.: Fracturing of polycrystalline MoS_2 nanofilms. ACS Appl. Electron. Mater. **2**(4), 1169–1175 (2020) https://doi.org/10.1021/acsaelm.0c00189

[23] Cui, S., Hu, B., Ouyang, B., Zhao, D.: Thermodynamic assessment of the Mo-S system and its application in thermal decomposition of MoS_2. Thermochim. Acta **660**, 44–55 (2018) https://doi.org/10.1016/j.tca.2017.12.011

[24] Cooper, R.C., Lee, C., Marianetti, C.A., Wei, X., Hone, J., Kysar, J.W.: Nonlinear elastic behavior of two-dimensional molybdenum disulfide. Phys. Rev. B **87**(3), 035423 (2013) https://doi.org/10.1103/PhysRevB.87.035423

[25] Peng, Q., De, S.: Outstanding mechanical properties of monolayer MoS_2 and its application in elastic energy storage. Phys. Chem. Chem. Phys. **15**(44), 19427–19437 (2013) https://doi.org/10.1039/C3CP52879K

[26] Woo, S., Park, H.C., Son, Y.-W.: Poisson's ratio in layered two-dimensional crystals. Phys. Rev. B **93**(7), 075420 (2016) https://doi.org/10.1103/PhysRevB.93.075420

[27] P., M., Vargas, M.D., Werlang, M.M., Yoshida, I.V.P., Mauler, R.S.: High-density polyethylene modified by polydimethylsiloxane. Journal of Applied Polymer Science **82**(14), 3460–3467 (2001) https://doi.org/10.1002/app.2207

[28] Ariati, R., Sales, F., Souza, A., Lima, R.A., Ribeiro, J.: Polydimethylsiloxane composites characterization and its applications: A review. Polymers **13**(23), 4258 (2021) https://doi.org/10.3390/polym13234258

[29] Camino, G., Lomakin, S.M., Lazzari, M.: Polydimethylsiloxane thermal degradation. Part 1. Kinetic aspects. Polymer **42**(6), 2395–2402 (2001) https://doi.org/10.1016/S0032-3861(00)00652-2

[30] Petersen, K.E.: Silicon as a mechanical material. Proceedings of the IEEE **70**(5), 420–457 (1982) https://doi.org/10.1109/PROC.1982.12331

[31] Sato, K., Yoshioka, T., Ando, T., Shikida, M., Kawabata, T.: Tensile testing of silicon film having different crystallographic orientations carried out on a silicon chip. Sensors and Actuators A: Physical **70**(1), 148–152 (1998) https://doi.org/10.1016/S0924-4247(98)00125-3

[32] Sharpe, W.N., Turner, K.T., Edwards, R.L.: Tensile testing of polysilicon. Experimental Mechanics **39**(3), 162–170 (1999) https://doi.org/10.1007/BF02323548

[33] Tsuchiya, T.: Tensile testing of silicon thin films. Fatigue & Fracture of Engineering Materials & Structures **28**(8), 665–674 (2005) https://doi.org/10.1111/j.1460-2695.2005.00910.x

[34] Tsuchiya, T., Ikeda, T., Tsunematsu, A., Sugano, K., Tabata, O.: Tensile testing of single-crystal silicon thin films at 600°C using infrared radiation heating. Sensors and Materials **22**(1), 1–11 (2010) https://doi.org/10.18494/SAM.2010.619

[35] Nannichi, Y.: Sublimation rate of silicon in high vacuum. Japanese Journal of Applied Physics **2**(9), 586–587 (1963) https://doi.org/10.1143/JJAP.2.586

[36] Yen, B.K., White, R.L., Waltman, R.J., Dai, Q., Miller, D.C., Kellock, A.J., Marchon, B., Kasai, P.H., Toney, M.F., York, B.R., Deng, H., Xiao, Q.-F., Raman, V.: Microstructure and properties of ultrathin amorphous silicon nitride protective coating. Journal of Vacuum Science & Technology A **21**(6), 1895–1904 (2003) https://doi.org/10.1116/1.1615974

[37] Yoshioka, T., Ando, T., Shikida, M., Sato, K.: Tensile testing of SiO_2 and Si_3N_4 films carried out on a silicon chip. Sensors and Actuators A: Physical **82**(1), 291–296 (2000) https://doi.org/10.1016/S0924-4247(99)00364-7

[38] Batha, H.D., Whitney, E.D.: Kinetics and mechanism of the thermal decomposition of Si_3N_4. Journal of the American Ceramic Society **56**(7), 365–369 (1973) https://doi.org/10.1111/j.1151-2916.1973.tb12687.x

[39] Kawase, K., Noda, S., Nakai, T., Uehara, Y.: Densification of chemical vapor deposition silicon dioxide film using ozone treatment. Japanese Journal of Applied Physics **48**(10R), 101401 (2009) https://doi.org/10.1143/JJAP.48.101401

[40] Tsuchiya, T., Inoue, A., Sakata, J.: Tensile testing of insulating thin films; humidity effect on tensile strength of SiO_2 films. Sensors and Actuators A: Physical **82**(1), 286–290 (2000) https://doi.org/10.1016/S0924-4247(99)00363-5

[41] Liehr, M., Lewis, J.E., Rubloff, G.W.: Kinetics of high-temperature thermal decomposition of SiO_2 on Si(100). Journal of Vacuum Science & Technology A **5**(4), 1559–1562 (1987) https://doi.org/10.1116/1.574564

[42] Saari, J., Ali-Löytty, H., Lahtonen, K., Hannula, M., Palmolahti, L., Tukiainen, A., Valden, M.: Low-temperature route to direct

amorphous to rutile crystallization of TiO_2 thin films grown by atomic layer deposition. The Journal of Physical Chemistry C **126**(36), 15357–15366 (2022) https://doi.org/10.1021/acs.jpcc.2c04905

[43] Borgese, L., Gelfi, M., Bontempi, E., Goudeau, P., Geandier, G., Thiaudière, D., Depero, L.E.: Young modulus and Poisson ratio measurements of TiO_2 thin films deposited with atomic layer deposition. Surface and Coatings Technology **206**(8), 2459–2463 (2012) https://doi.org/10.1016/j.surfcoat.2011.10.050

[44] Tavares, C.J., Marques, S.M., Lanceros-Méndez, S., Sencadas, V., Teixeira, V., Carneiro, J.O., Martins, A.J., Fernandes, A.J.: Strain analysis of photocatalytic TiO_2 thin films on polymer substrates. Thin Solid Films **516**(7), 1434–1438 (2008) https://doi.org/10.1016/j.tsf.2007.03.134

[45] Mizuno, Y., King, F.K., Yamauchi, Y., Homma, T., Tanaka, A., Takakuwa, Y., Momose, T.: Temperature dependence of oxide decomposition on titanium surfaces in ultrahigh vacuum. Journal of Vacuum Science & Technology A **20**(5), 1716–1721 (2002) https://doi.org/10.1116/1.1500746

[46] Ilic, O., Went, C.M., Atwater, H.A.: Nanophotonic heterostructures for efficient propulsion and radiative cooling of relativistic light sails. Nano Lett. **18**(9), 5583–5589 (2018) https://doi.org/10.1021/acs.nanolett.8b02035

[47] Salary, M.M., Mosallaei, H.: Photonic metasurfaces as relativistic light sails for Doppler-broadened stable beam-riding and radiative cooling. Laser Photonics Rev. **14**(8), 1900311 (2020) https://doi.org/10.1002/lpor.201900311

[48] Brewer, J., Campbell, M.F., Kumar, P., Kulkarni, S., Jariwala, D., Bargatin, I., Raman, A.P.: Multiscale photonic emissivity engineering for relativistic lightsail thermal regulation. Nano Letters **22**(2), 594–601 (2022) https://doi.org/10.1021/acs.nanolett.1c03273

[49] Lien, M.R., Meng, D., Liu, Z., Sakib, M.A., Tang, Y., Wu, W., Povinelli, M.L.: Experimental characterization of a silicon nitride photonic crystal light sail. Opt. Mater. Express **12**(8), 3032–3042 (2022) https://doi.org/10.1364/OME.464430

[50] Santi, G., Favaro, G., Corso, A.J., Lubin, P., Bazzan, M., Ragazzoni, R., Garoli, D., Pelizzo, M.G.: Multilayers for directed energy accelerated lightsails. Communications Materials **3**(1), 16 (2022) https://doi.org/10.1038/s43246-022-00240-8

[51] Taghavi, M., Mosallaei, H.: Increasing the stability margins using multi-pattern metasails and multi-modal laser beams. Scientific Reports **12**(1), 20034 (2022) https://doi.org/10.1038/s41598-022-24681-w

[52] Chang, J., Ji, W., Yao, X., Run, A.J., Gröblacher, S.: Broadband, high-reflectivity dielectric mirrors at wafer scale: Combining photonic crystal and metasurface architectures for advanced lightsails. Nano Letters **24**(22), 6689–6695 (2024) https://doi.org/10.1021/acs.nanolett.4c01374

[53] Norder, L., Yin, S., de Jong, M.H.J., Stallone, F., Aydogmus, H., Sberna, P.M., Bessa, M.A., Norte, R.A.: Pentagonal photonic crystal mirrors: Scalable lightsails with enhanced acceleration via neural topology optimization. Nature Communications **16**(1), 2753 (2025) https://doi.org/10.1038/s41467-025-57749-y

[54] Whittam, M.R., Rebholz, L., Zerulla, B., Rockstuhl, C.: Analyzing the acceleration time and reflectance of light sails made from homogeneous and core-shell spheres. Opt. Mater. Express **15**(2), 345–361 (2025) https://doi.org/10.1364/OME.545481

[55] Bergeron, H., Sangwan, V.K., McMorrow, J.J., Campbell, G.P., Balla, I., Liu, X., Bedzyk, M.J., Marks, T.J., Hersam, M.C.: Chemical vapor deposition of monolayer MoS_2 directly on ultrathin Al_2O_3 for low-power electronics. Applied Physics Letters **110**(5), 053101 (2017) https://doi.org/10.1063/1.4975064

[56] Song, X., Xu, J., Liu, L.: High-quality CVD-MoS_2 synthesized on surface-modified Al_2O_3 for high-performance MoS_2 field-effect transistors. IEEE Transactions on Electron Devices **67**(11), 5196–5200 (2020) https://doi.org/10.1109/TED.2020.3021998

[57] Gao, Q., Lu, J., Chen, S., Chen, L., Xu, Z., Lin, D., Xu, S., Liu, P., Zhang, X., Cai, W., Zhang, C.: Chemical vapor deposition of uniform and large-domain molybdenum disulfide crystals on glass/Al_2O_3 substrates. Nanomaterials **12**(15), 2719 (2022) https://doi.org/10.3390/nano12152719

[58] Azcatl, A., McDonnell, S., K. C., S., Peng, X., Dong, H., Qin, X., Addou, R., Mordi, G.I., Lu, N., Kim, J., Kim, M.J., Cho, K., Wallace, R.M.: MoS_2 functionalization for ultra-thin atomic layer deposited dielectrics. Applied Physics Letters **104**(11), 111601 (2014) https://doi.org/10.1063/1.4869149

[59] Song, J.-G., Kim, S.J., Woo, W.J., Kim, Y., Oh, I.-K., Ryu, G.H., Lee, Z., Lim, J.H., Park, J., Kim, H.: Effect of Al_2O_3 deposition on performance of top-gated monolayer MoS_2-based field effect transistor. ACS Applied Materials & Interfaces **8**(41), 28130–28135 (2016) https://doi.org/10.1021/acsami.6b07271

[60] Zhang, H., Chiappe, D., Meersschaut, J., Conard, T., Franquet, A., Nuytten, T., Mannarino, M., Radu, I., Vandervorst, W., Delabie, A.: Nucleation and growth mechanisms of Al_2O_3 atomic layer deposition on synthetic polycrystalline MoS_2. The Journal of Chemical Physics **146**(5), 052810 (2016) https://doi.org/10.1063/1.4967406

[61] Park, T., Kim, H., Leem, M., Ahn, W., Choi, S., Kim, J., Uh, J., Kwon, K., Jeong, S.-J., Park, S., Kim, Y., Kim, H.: Atomic layer deposition of Al_2O_3 on MoS_2, WS_2, WSe_2, and h-BN: Surface coverage and adsorption energy. RSC Adv. **7**(2), 884–889 (2017) https://doi.org/10.1039/C6RA24733D

[62] Kropp, J.A., Cai, Y., Yao, Z., Zhu, W., Gougousi, T.: Atomic layer deposition of Al_2O_3 and TiO_2 on MoS_2 surfaces. Journal of Vacuum Science & Technology A **36**(6), 06–101 (2018) https://doi.org/10.1116/1.5043621

[63] Huang, B., Zheng, M., Zhao, Y., Wu, J., Thong, J.T.L.: Atomic layer deposition of high-quality Al_2O_3 thin films on MoS_2 with

water plasma treatment. ACS Applied Materials & Interfaces **11**(38), 35438–35443 (2019) https://doi.org/10.1021/acsami.9b10940

[64] Li, N., Wei, Z., Zhao, J., Wang, Q., Shen, C., Wang, S., Tang, J., Yang, R., Shi, D., Zhang, G.: Atomic layer deposition of Al_2O_3 directly on 2D materials for high-performance electronics. Advanced Materials Interfaces **6**(10), 1802055 (2019) https://doi.org/10.1002/admi.201802055

[65] Schilirò, E., Nigro, R.L., Panasci, S.E., Agnello, S., Cannas, M., Gelardi, F.M., Roccaforte, F., Giannazzo, F.: Direct atomic layer deposition of ultrathin aluminum oxide on monolayer MoS_2 exfoliated on gold: The role of the substrate. Advanced Materials Interfaces **8**(21), 2101117 (2021) https://doi.org/10.1002/admi.202101117

[66] Schilirò, E., Panasci, S.E., Mio, A.M., Nicotra, G., Agnello, S., Pecz, B., Radnoczi, G.Z., Deretzis, I., La Magna, A., Roccaforte, F., Lo Nigro, R., Giannazzo, F.: Direct atomic layer deposition of ultra-thin Al_2O_3 and HfO_2 films on gold-supported monolayer MoS_2. Applied Surface Science **630**, 157476 (2023) https://doi.org/10.1016/j.apsusc.2023.157476

[67] Vitale, S.M., Sugar, J.D.: Using Xe plasma FIB for high-quality TEM sample preparation. Microscopy and Microanalysis **28**(3), 646–658 (2022) https://doi.org/10.1017/S1431927622000344

[68] Kim, K.-H., Oh, S., Fiagbenu, M.M.A., Zheng, J., Musavigharavi, P., Kumar, P., Trainor, N., Aljarb, A., Wan, Y., Kim, H.M., Katti, K., Song, S., Kim, G., Tang, Z., Fu, J.-H., Hakami, M., Tung, V., Redwing, J.M., Stach, E.A., Olsson, R.H. III, Jariwala, D.: Scalable CMOS back-end-of-line-compatible AlScN/two-dimensional channel ferroelectric field-effect transistors. Nature Nanotechnology **18**(9), 1044–1050 (2023) https://doi.org/10.1038/s41565-023-01399-y

[69] Fei, L., Lei, S., Zhang, W.-B., Lu, W., Lin, Z., Lam, C.H., Chai, Y., Wang, Y.: Direct TEM observations of growth mechanisms of two-dimensional MoS_2 flakes. Nature Communications **7**(1), 12206 (2016) https://doi.org/10.1038/ncomms12206

[70] Kumar, P., Viswanath, B.: Horizontally and vertically aligned growth of strained MoS_2 layers with dissimilar wetting and catalytic behaviors. CrystEngComm **19**(34), 5068–5078 (2017) https://doi.org/10.1039/C7CE01162H

[71] Altvater, M., Muratore, C., Snure, M., Glavin, N.R.: Two-step conversion of metal and metal oxide precursor films to 2D transition metal dichalcogenides and heterostructures. Small, 2400463 (2024) https://doi.org/10.1002/smll.202400463

[72] Winters, H.F., Coburn, J.W.: The etching of silicon with XeF_2 vapor. Applied Physics Letters **34**(1), 70–73 (1979) https://doi.org/10.1063/1.90562

[73] Veyan, J.-F., Halls, M.D., Rangan, S., Aureau, D., Yan, X.-M., Chabal, Y.J.: XeF_2-induced removal of SiO_2 near Si surfaces at 300 K: An unexpected proximity effect. Journal of Applied Physics **108**(11), 114914 (2010) https://doi.org/10.1063/1.3517148

[74] Davami, K., Zhao, L., Lu, E., Cortes, J., Lin, C., Lilley, D.E., Purohit, P.K., Bargatin, I.: Ultralight shape-recovering plate mechanical metamaterials. Nature Communications **6**(1), 10019 (2015) https://doi.org/10.1038/ncomms10019

[75] Foral, R.F.: Composite spherical pressure vessels with hardening metal liners. J. Press. Vessel Technol. **101**(3), 200–206 (1979) https://doi.org/10.1115/1.3454623

[76] Roy, A.K., Massard, T.N.: A design study of thick multilayered composite spherical pressure vessels. J. Reinf. Plast. Compos. **11**(5), 479–493 (1992) https://doi.org/10.1177/073168449201100502

[77] Liu, B., Feng, X., Zhang, S.-M.: The effective Young's modulus of composites beyond the Voigt estimation due to the Poisson effect. Composites Science and Technology **69**(13), 2198–2204 (2009) https://doi.org/10.1016/j.compscitech.2009.06.004

[78] You, Y.-J., Kim, J.-H.J., Park, K.-T., Seo, D.-W., Lee, T.-H.: Modification of rule of mixtures for tensile strength estimation of circular GFRP rebars. Polymers **9**(12), 682 (2017) https://doi.org/10.3390/polym9120682

[79] Raju, B., Hiremath, S.R., Roy Mahapatra, D.: A review of micromechanics based models for effective elastic properties of reinforced polymer matrix composites. Composite Structures **204**, 607–619 (2018) https://doi.org/10.1016/j.compstruct.2018.07.125

[80] Jiao, P., Nicaise, S.M., Azadi, M., Cortes, J., Lilley, D.E., Cha, W., Purohit, P.K., Bargatin, I.: Tunable tensile response of honeycomb plates with nanoscale thickness: Testing and modeling. Extreme Mechanics Letters **34**, 100599 (2020) https://doi.org/10.1016/j.eml.2019.100599

[81] Jiao, P., Nicaise, S.M., Lin, C., Purohit, P.K., Bargatin, I.: Extremely sharp bending and recoverability of nanoscale plates with honeycomb corrugation. Phys. Rev. Appl. **11**(3), 034055 (2019) https://doi.org/10.1103/PhysRevApplied.11.034055

[82] Babacic, V., Saleta Reig, D., Varghese, S., Vasileiadis, T., Coy, E., Tielrooij, K.-J., Graczykowski, B.: Thickness-dependent elastic softening of few-layer free-standing $MoSe_2$. Advanced Materials **33**(23), 2008614 (2021) https://doi.org/10.1002/adma.202008614

[83] Gayler, M.L.V.: Melting point of high-purity silicon. Nature **142**(3593), 478 (1938) https://doi.org/10.1038/142478a0

[84] Holdman, G.R., Jaffe, G.R., Feng, D., Jang, M.S., Kats, M.A., Brar, V.W.: Thermal runaway of silicon-based laser sails. Advanced Optical Materials **10**(19), 2102835 (2022) https://doi.org/10.1002/adom.202102835

[85] Rumble, J.R.: CRC Handbook of Chemistry and Physics, 105th edn. CRC Press, Boca Raton, FL (2024)

[86] St. Pierre, P.D.S.: A note on the melting point of titanium dioxide. Journal of the American Ceramic Society **35**(7), 188–188 (1952) https://doi.org/10.1111/j.1151-2916.1952.tb13097.x

[87] Campbell, M.F., Brewer, J., Jariwala, D., Raman, A.P., Bargatin, I.: Relativistic light sails need to billow. Nano Letters **22**(1), 90–96 (2022) https://doi.org/10.1021/acs.nanolett.1c03272

[88] Parkin, K.L.G.: The Breakthrough Starshot system model. Acta Astronaut. **152**, 370–384 (2018) https://doi.org/10.1016/j.actaastro.2018.08.035

[89] Kulkarni, N., Lubin, P.M., Zhang, Q.: Relativistic solutions to directed energy. In: Hughes, G.B. (ed.) Planetary Defense and Space Environment Applications, vol. 9981, pp. 43–52. SPIE, Bellingham, WA, USA (2016). https://doi.org/10.1117/12.2238094

[90] Füzfa, A., Dhelonga-Biarufu, W., Welcomme, O.: Sailing towards the stars close to the speed of light. Phys. Rev. Research **2**(4), 043186 (2020) https://doi.org/10.1103/PhysRevResearch.2.043186

[91] Pegoraro, F., Livi, C., Macchi, A.: Light sail boosted by instantaneous radiation pressure. Eur. Phys. J. Plus **136**(5), 485 (2021) https://doi.org/10.1140/epjp/s13360-021-01357-4

[92] Atwater, H.A., Davoyan, A.R., Ilic, O., Jariwala, D., Sherrott, M.C., Went, C.M., Whitney, W.S., Wong, J.: Materials challenges for the Starshot lightsail. Nat. Mater. **17**(10), 861–867 (2018) https://doi.org/10.1038/s41563-018-0075-8

[93] Kulkarni, N., Lubin, P., Zhang, Q.: Relativistic spacecraft propelled by directed energy. Astron. J. **155**(4), 155 (2018) https://doi.org/10.3847/1538-3881/aaafd2

[94] Nichols, E.F., Hull, G.F.: The pressure due to radiation. Astrophys. J. **17**(5), 315–351 (1903) https://doi.org/10.1086/141035

[95] Ohta, K., Ishida, H.: Matrix formalism for calculation of the light beam intensity in stratified multilayered films, and its use in the analysis of emission spectra. Appl. Opt. **29**(16), 2466–2473 (1990) https://doi.org/10.1364/AO.29.002466

[96] Macleod, H.A.: Thin-film Optical Filters, 5th edn. CRC Press, New York, NY, USA (2017)

[97] Boyajian, A.: Physical interpretation of complex angles and their functions. J. Am. Inst. Electr. Eng. **42**(2), 155–164 (1923) https://doi.org/10.1109/JoAIEE.1923.6592034

[98] Popova, E., Efendiev, M., Gabitov, I.: On the stability of a space vehicle riding on an intense laser beam. Math. Meth. Appl. Sci. **40**, 1346–1354 (2016) https://doi.org/10.1002/mma.4282

[99] Gieseler, N., Rahimzadegan, A., Rockstuhl, C.: Self-stabilizing curved metasurfaces as a sail for light-propelled spacecrafts. Opt. Express **29**(14), 21562–21575 (2021) https://doi.org/10.1364/OE.420475

[100] Gao, R., Kelzenberg, M.D., Atwater, H.A.: Dynamically stable radiation pressure propulsion of flexible lightsails for interstellar exploration. Nature Communications **15**(1), 4203 (2024) https://doi.org/10.1038/s41467-024-47476-1

[101] Mistrik, J., Krbal, M., Prokop, V., Prikryl, J.: Giant change of MoS_2 optical properties along amorphous–crystalline transition: Broadband spectroscopic study including the NIR therapeutic window. Nanoscale Adv. **5**(11), 2911–2920 (2023) https://doi.org/10.1039/D3NA00111C

[102] Fujiwara, H.: Spectroscopic Ellipsometry: Principles and Applications. John Wiley & Sons, Ltd., Chichester, UK (2007). https://doi.org/10.1002/9780470060193

[103] Beal, A.R., Hughes, H.P.: Kramers-Krönig analysis of the reflectivity spectra of 2H-MoS_2, 2H-$MoSe_2$, and 2H-$MoTe_2$. J. Phys. C: Solid State Phys. **12**(5), 881–890 (1979) https://doi.org/10.1088/0022-3719/12/5/017

[104] Roxlo, C.B., Chianelli, R.R., Deckman, H.W., Ruppert, A.F., Wong, P.P.: Bulk and surface optical absorption in molybdenum disulfide. Journal of Vacuum Science & Technology A **5**(4), 555–557 (1987) https://doi.org/10.1116/1.574671

[105] Yim, C., O'Brien, M., McEvoy, N., Winters, S., Mirza, I., Lunney, J.G., Duesberg, G.S.: Investigation of the optical properties of MoS_2 thin films using spectroscopic ellipsometry. Appl. Phys. Lett. **104**(10), 103114 (2014) https://doi.org/10.1063/1.4868108

[106] Hsu, C., Frisenda, R., Schmidt, R., Arora, A., de Vasconcellos, S.M., Bratschitsch, R., van der Zant, H.S.J., Castellanos-Gomez, A.: Thickness-dependent refractive index of 1L, 2L, and 3L MoS_2, $MoSe_2$, WS_2, and WSe_2. Advanced Optical Materials **7**(13), 1900239 (2019) https://doi.org/10.1002/adom.201900239

[107] Ermolaev, G.A., Grudinin, D.V., Stebunov, Y.V., Voronin, K.V., Kravets, V.G., Duan, J., Mazitov, A.B., Tselikov, G.I., Bylinkin, A., Yakubovsky, D.I., Novikov, S.M., Baranov, D.G., Nikitin, A.Y., Kruglov, I.A., Shegai, T., Alonso-González, P., Grigorenko, A.N., Arsenin, A.V., Novoselov, K.S., Volkov, V.S.: Giant optical anisotropy in transition metal dichalcogenides for next-generation photonics. Nat. Commun. **12**(1), 854 (2021) https://doi.org/10.1038/s41467-021-21139-x

[108] Zotev, P.G., Wang, Y., Andres-Penares, D., Severs-Millard, T., Randerson, S., Hu, X., Sortino, L., Louca, C., Brotons-Gisbert, M., Huq, T., Vezzoli, S., Sapienza, R., Krauss, T.F., Gerardot, B.D., Tartakovskii, A.I.: Van der Waals materials for applications in nanophotonics. Laser & Photonics Reviews **17**(8), 2200957 (2023) https://doi.org/10.1002/lpor.202200957

[109] Polyanskiy, M.N.: Refractiveindex.info database of optical constants. Scientific Data **11**(1), 94 (2024) https://doi.org/10.1038/s41597-023-02898-2

[110] Lee, D.W., Kingery, W.D.: Radiation energy transfer and thermal conductivity of ceramic oxides. J. Am. Ceram. Soc. **43**(11), 594–607 (1960) https://doi.org/10.1111/j.1151-2916.1960.tb13623.x

[111] Oppenheim, U.P., Even, U.: Infrared properties of sapphire at elevated temperatures. J. Opt. Soc. Am. **52**(9), 1078–1079 (1962) https://doi.org/10.1364/JOSA.52.1078_1

[112] Gillespie, D.T., Olsen, A.L., Nichols, L.W.: Transmittance of optical materials at high temperatures in the 1-μ to 12-μ range. Appl. Opt. **4**(11), 1488–1493 (1965) https://doi.org/10.1364/AO.4.001488

[113] Gryvnak, D.A., Burch, D.E.: Optical and infrared properties of Al_2O_3 at elevated temperatures. J. Opt. Soc. Am. **55**(6), 625–629 (1965) https://doi.org/10.1364/JOSA.55.000625

[114] Billard, D., Piriou, B.: Absorption infrarouge du corindon de 77 a 2075 K. Mater. Res. Bull. **9**(7), 943–950 (1974) https://doi.org/10.1016/0025-5408(74)90174-3

[115] Billard, D., Gervais, F., Piriou, B.: Analysis of multiphonon absorption in corundum. Phys. Stat. Sol. (B) **75**(1), 117–126 (1976) https://doi.org/10.1002/pssb.2220750111

[116] Malitson, I.H.: Refraction and dispersion of synthetic sapphire. J. Opt. Soc. Am. **52**(12), 1377–1379 (1962) https://doi.org/10.1364/JOSA.52.001377

[117] Hagemann, H.-J., Gudat, W., Kunz, C.: Optical constants from the far infrared to the X-ray region: Mg, Al, Cu, Ag, Au, Bi, C, and Al_2O_3. J. Opt. Soc. Am. **65**(6), 742–744 (1975) https://doi.org/10.1364/JOSA.65.000742

[118] Billard, D., Gervais, F., Piriou, B.: Farinfrared absorption in Al_2O_3 and MgO. Int. J. Infrared and Millim. Waves **1**(4), 641–647 (1980) https://doi.org/10.1007/BF01013473

[119] Cabannes, F., Billard, D.: Measurement of infrared absorption of some oxides in connection with the radiative transfer in porous and fibrous materials. Int. J. Thermophys. **8**(1), 97–118 (1987) https://doi.org/10.1007/BF00503227

[120] Sarou-Kanian, V., Rifflet, J.C., Millot, F.: IR radiative properties of solid and liquid alumina: Effects of temperature and gaseous environment. Int. J. Thermophys. **26**(4), 1263–1275 (2005) https://doi.org/10.1007/s10765-005-6725-5

[121] Lee, G.W., Jeon, S., Park, S.-N., Yoo, Y.S., Park, C.-W.: Temperature and thickness dependence of IR optical properties of sapphire at moderate temperature. Int. J. Thermophys. **32**(7), 1448–1456 (2011) https://doi.org/10.1007/s10765-011-0990-2

[122] Franta, D., Nečas, D., Ohlídal, I., Giglia, A.: Dispersion model for optical thin films applicable in wide spectral range. In: Duparré, A., Geyl, R. (eds.) Optical Systems Design 2015: Optical Fabrication, Testing, and Metrology V, vol. 9628, p. 96281. SPIE, Bellingham, WA, USA (2015). https://doi.org/10.1117/12.2190104

[123] Kalman, J., Allen, D., Glumac, N., Krier, H.: Optical depth effects on aluminum oxide spectral emissivity. J. Thermophys. Heat Trans. **29**(1), 74–82 (2015) https://doi.org/10.2514/1.T4260

[124] Yang, J.Y., Xu, M., Liu, L.H.: Infrared radiative properties of alumina up to the melting point: A first-principles study. J. Quant. Spectrosc. Radiat. Transf. **184**, 111–117 (2016) https://doi.org/10.1016/j.jqsrt.2016.07.006

[125] Boidin, R., Halenkovič, T., Nazabal, V., Beneš, L., Němec, P.: Pulsed laser deposited alumina thin films. Ceram. **42**(1, Part B), 1177–1182 (2016) https://doi.org/10.1016/j.ceramint.2015.09.048

[126] Poruba, A., Fejfar, A., Remeš, Z., Špringer, J., Vaněček, M., Kočka, J., Meier, J., Torres, P., Shah, A.: Optical absorption and light scattering in microcrystalline silicon thin films and solar cells. Journal of Applied Physics **88**(1), 148–160 (2000) https://doi.org/10.1063/1.373635

[127] Franta, D., Dubroka, A., Wang, C., Giglia, A., Vohánka, J., Franta, P., Ohlídal, I.: Temperature-dependent dispersion model of float zone crystalline silicon. Applied Surface Science **421**, 405–419 (2017) https://doi.org/10.1016/j.apsusc.2017.02.021

[128] Marcos, L.V.R.-d., Larruquert, J.I., Méndez, J.A., Aznárez, J.A.: Self-consistent optical constants of SiO_2 and Ta_2O_5 films. Opt. Mater. Express **6**(11), 3622–3637 (2016) https://doi.org/10.1364/OME.6.003622

[129] Olmon, R.L., Slovick, B., Johnson, T.W., Shelton, D., Oh, S.-H., Boreman, G.D., Raschke, M.B.: Optical dielectric function of gold. Phys. Rev. B **86**(23), 235147 (2012) https://doi.org/10.1103/PhysRevB.86.235147

[130] Schinke, C., Christian Peest, P., Schmidt, J., Brendel, R., Bothe, K., Vogt, M.R., Kröger, I., Winter, S., Schirmacher, A., Lim, S., Nguyen, H.T., MacDonald, D.: Uncertainty analysis for the coefficient of band-to-band absorption of crystalline silicon. AIP Advances **5**(6), 067168 (2015) https://doi.org/10.1063/1.4923379

[131] Parkin, K.L.G.: Cost-optimal laser-accelerated lightsails. Preprint at https://arxiv.org/abs/2205.13138 (2023)

[132] Phipps, C. (ed.): Laser Propulsion in Space: Fundamentals, Technology, and Future Missions, 1st edn. Elsevier, New York, NY, USA (2024)

[133] Gordon, J.E.: Structures or Why Things Don't Fall Down. Plenum Press, New York, NY, USA (1978). Chapter 6: Tension structures and pressure vessels - with some remarks on boilers, bats, and Chinese junks

[134] Leff, H.S.: Teaching the photon gas in introductory physics. Am. J. Phys. **70**(8), 792–797 (2002) https://doi.org/10.1119/1.1479743

[135] Sakamoto, H., Miyazaki, Y., Park, K.C.: Finite element modeling of sail deformation under solar radiation pressure. J. Spacecr. Rockets **44**(3), 514–521 (2007) https://doi.org/10.2514/1.23474

[136] Timoshenko, S., Woinowsky-Krieger, S.: Theory of Plates and Shells. McGraw-Hill Book Company, New York, NY, USA (1959)

[137] Lee, C., Wei, X., Kysar, J.W., Hone, J.: Measurement of the elastic properties and intrinsic strength of monolayer graphene. Science **321**(5887), 385–388 (2008) https://doi.org/10.1126/science.1157996

[138] Yokoyama, T., Nakai, K., Odamura, T.: Tensile stress-strain properties of paper and paperboard and their constitutive equations. Journal of the Japanese Society for Experimental Mechanics **7**, 68–73 (2007) https://doi.org/10.11395/jjsem.7.s68

[139] Lubin, P.: A roadmap to interstellar flight. J. Br. Interplanet. Soc. **69**, 40–72 (2016)

[140] Tung, H.-T., Davoyan, A.R.: Low-power laser sailing for fast-transit space flight. Nano Letters **22**(3), 1108–1114 (2022) https://doi.org/10.1021/acs.nanolett.1c04188

[141] Jin, W., Li, W., Orenstein, M., Fan, S.: Inverse design of lightweight broadband reflector for relativistic lightsail propulsion. ACS Photonics **7**(9), 2350–2355 (2020) https://doi.org/10.1021/acsphotonics.0c00768

[142] Kudyshev, Z.A., Kildishev, A.V., Shalaev, V.M., Boltasseva, A.: Optimizing startshot lightsail design: A generative network-based approach. ACS Photonics **9**(1), 190–196 (2022) https://doi.org/10.1021/acsphotonics.1c01352

[143] Gao, R., Kelzenberg, M.D., Kim, Y., Ilic, O., Atwater, H.A.: Optical characterization of silicon nitride metagrating-based lightsails for self-stabilization. ACS Photonics **9**(6), 1965–1972 (2022) https://doi.org/10.1021/acsphotonics.1c02022

[144] Siegel, J., Wang, A.Y., Menabde, S.G., Kats, M.A., Jang, M.S., Brar, V.W.: Self-stabilizing laser sails based on optical metasurfaces. ACS Photonics **6**(8), 2032–2040 (2019) https://doi.org/10.1021/acsphotonics.9b00484

[145] Myilswamy, K.V., Krishnan, A., Povinelli, M.L.: Photonic crystal lightsail with nonlinear reflectivity for increased stability. Opt. Express **28**(6), 8223–8232 (2020) https://doi.org/10.1364/OE.387687

[146] El-Haija, A.J.A.: Effective medium approximation for the effective optical constants of a bilayer and a multilayer structure based on the characteristic matrix technique. Journal of Applied Physics **93**(5), 2590–2594 (2003) https://doi.org/10.1063/1.1543229

[147] Zhang, X., Qiu, J., Zhao, J., Li, X., Liu, L.: Complex refractive indices measurements of polymers in infrared bands. Journal of Quantitative Spectroscopy and Radiative Transfer **252**, 107063 (2020) https://doi.org/10.1016/j.jqsrt.2020.107063

[148] Zhang, X., Qiu, J., Li, X., Zhao, J., Liu, L.: Complex refractive indices measurements of polymers in visible and near-infrared bands. Appl. Opt. **59**(8), 2337–2344 (2020) https://doi.org/10.1364/AO.383831

[149] Feynman, R.P.: There's plenty of room at the bottom. Engineering and Science **23**(5), 22–36 (1960). URL: https://resolver.caltech.edu/CaltechES:23.5.0

[150] Waldrop, M.M.: The chips are down for Moore's law. Nature **530**(7589), 144–147 (2016) https://doi.org/10.1038/530144a

[151] Broas, M., Kanninen, O., Vuorinen, V., Tilli, M., Paulasto-Kröckel, M.: Chemically stable atomic-layer-deposited Al_2O_3 films for processability. ACS Omega **2**(7), 3390–3398 (2017) https://doi.org/10.1021/acsomega.7b00443

[152] Broas, M., Lemettinen, J., Sajavaara, T., Tilli, M., Vuorinen, V., Suihkonen, S., Paulasto-Kröckel, M.: In-situ annealing characterization of atomic-layer-deposited Al_2O_3 in N_2, H_2 and vacuum atmospheres. Thin Solid Films **682**, 147–155 (2019) https://doi.org/10.1016/j.tsf.2019.03.010

[153] Zhang, L., Jiang, H.C., Liu, C., Dong, J.W., Chow, P.: Annealing of Al_2O_3 thin films prepared by atomic layer deposition. Journal of Physics D: Applied Physics **40**(12), 3707 (2007) https://doi.org/10.1088/0022-3727/40/12/025

[154] Wang, Z.-Y., Zhang, R.-J., Lu, H.-L., Chen, X., Sun, Y., Zhang, Y., Wei, Y.-F., Xu, J.-P., Wang, S.-Y., Zheng, Y.-X., Chen, L.-Y.: The impact of thickness and thermal annealing on refractive index for aluminum oxide thin films deposited by atomic layer deposition. Nanoscale Research Letters **10**(1), 46 (2015) https://doi.org/10.1186/s11671-015-0757-y

[155] Ylivaara, O.M.E., Langner, A., Ek, S., Malm, J., Julin, J., Laitinen, M., Ali, S., Sintonen, S., Lipsanen, H., Sajavaara, T., Puurunen, R.L.: Thermomechanical properties of aluminum oxide thin films made by atomic layer deposition. Journal of Vacuum Science & Technology A **40**(6), 062414 (2022) https://doi.org/10.1116/6.0002095

[156] Lu, X., Utama, M.I.B., Zhang, J., Zhao, Y., Xiong, Q.: Layer-by-layer thinning of MoS_2 by thermal annealing. Nanoscale **5**(19), 8904–8908 (2013) https://doi.org/10.1039/C3NR03101B

[157] Omashova, G., Tussupzhanov, A., Ramankulov, S., Katpayeva, K., Baltabaeyeva, D., Mussakhan, N., Kaldar, B.: Recent advances in MoS_2-based nanocomposites: Synthesis, structural features, and electrochemical applications. Crystals **15**(12), 1037 (2025) https://doi.org/10.3390/cryst15121037

[158] Zhu, X., Jung, G.S., Yang, J., Hu, X., Zhou, X., Shi, Z., Ravel, V., Lin, C.-L., Peng, Z., Franklin, A., Ma, Q., Roy, T., Wang, H.: Chemical vapor deposition of uniform and large-scale MoS_2 using heterogeneous precursors. ChemRxiv (2025) https://doi.org/10.26434/chemrxiv-2025-tz2gv

[159] Aspiotis, N., Morgan, K., März, B., Müller-Caspary, K., Ebert, M., Weatherby, E., Light, M.E., Huang, C.-C., Hewak, D.W., Majumdar, S., Zeimpekis, I.: Large-area synthesis of high electrical performance MoS_2 by a commercially scalable atomic layer deposition process. npj 2D Materials and Applications **7**(1), 18 (2023) https://doi.org/10.1038/s41699-023-00379-z

[160] Imai, S., Tatsumi, T., Tomiya, S., Kakushima, K., Wakabayashi, H.: Improvement of MoS_2 film quality using sputtering processes controlling particle- and energy-flux followed by sulfur-vapor annealing. Japanese Journal of Applied Physics **64**(2), 021001 (2025) https://doi.org/10.35848/1347-4065/ada9df

[161] Mak, K.F., Lee, C., Hone, J., Shan, J., Heinz, T.F.: Atomically thin MoS_2: A new direct-gap semiconductor. Phys. Rev. Lett. **105**(13), 136805 (2010) https://doi.org/10.1103/PhysRevLett.105.136805

[162] Franta, D., Nečas, D., Ohlídal, I., Giglia, A.: Optical characterization of SiO_2 thin films using universal dispersion model over wide spectral range. In: Gorecki, C., Asundi, A.K., Osten, W. (eds.) Optical Micro- and Nanometrology VI, vol. 9890, p. 989014. SPIE, Bellingham, WA, USA (2016). https://doi.org/10.1117/12.2227580

[163] Shkondin, E., Takayama, O., Panah, M.E.A., Liu, P., Larsen, P.V., Mar, M.D., Jensen, F., Lavrinenko, A.V.: Large-scale high aspect ratio Al-doped ZnO nanopillars arrays as anisotropic metamaterials. Opt. Mater. Express **7**(5), 1606–1627 (2017) https://doi.org/10.1364/OME.7.001606

[164] Jaffe, G.R., Holdman, G.R., Jang, M.S., Feng, D., Kats, M.A., Brar, V.W.: Effect of dust and hot spots on the thermal stability of laser sails. Nano Letters **23**(15), 6852–6858 (2023) https://doi.org/10.1021/acs.nanolett.3c01069

[165] Brenner, S.S.: Mechanical behavior of sapphire whiskers at elevated temperatures. Journal of Applied Physics **33**(1), 33–39 (1962) https://doi.org/10.1063/1.1728523

[166] Sánchez-González, E., Miranda, P., Meléndez-Marténez, J.J., Guiberteau, F., Pajares, A.: Temperature dependence of mechanical properties of alumina up to the onset of creep. J. Eur. Ceram. Soc. **27**(11), 3345–3349 (2007) https://doi.org/10.1016/j.jeurceramsoc.2007.02.191

[167] Pham, V.-T., Fang, T.-H.: Thermal and mechanical characterization of nanoporous two-dimensional MoS_2 membranes. Scientific Reports **12**(1), 7777 (2022) https://doi.org/10.1038/s41598-022-11883-5

[168] Despont, M., Gross, H., Arrouy, F., Stebler, C., Staufer, U.: Fabrication of a silicon-Pyrex-silicon stack by A.C. anodic bonding. Sens. Actuators, A **55**(2), 219–224 (1996) https://doi.org/10.1016/S0924-4247(97)80081-7

[169] Touloukian, Y.S., Ho, C.Y.: Thermophysical properties of matter: The TPRC data series. Volume 13: Thermal expansion - Nonmetallic solids. Technical report, Purdue University (1977)

[170] Ristau, D., Jupé, M., Starke, K.: Laser damage thresholds of optical coatings. Thin Solid Films **518**(5), 1607–1613 (2009) https://doi.org/10.1016/j.tsf.2009.07.150

[171] Taylor, L.N., Brown, A.K., Pung, A.J., Johnson, E.G., Talghader, J.J.: Continuous-wave laser damage of uniform and nanolaminate hafnia and titania optical coatings. Opt. Lett. **38**(21), 4292–4295 (2013) https://doi.org/10.1364/OL.38.004292

[172] Tumkur, T.U., Sokhoyan, R., Su, M.P., Ceballos-Sanchez, A., Shirmanesh, G.K., Kim, Y., Atwater, H.A., Feigenbaum, E., Elhadj, S.: Toward high laser power beam manipulation with nanophotonic materials: Evaluating thin film damage performance. Opt. Express **29**(5), 7261–7275 (2021) https://doi.org/10.1364/OE.413843

[173] Atikian, H.A., Sinclair, N., Latawiec, P., Xiong, X., Meesala, S., Gauthier, S., Wintz, D., Randi, J., Bernot, D., DeFrances, S., Thomas, J., Roman, M., Durrant, S., Capasso, F., Lončar, M.: Diamond mirrors for high-power continuous-wave lasers. Nature Communications **13**(1), 2610 (2022) https://doi.org/10.1038/s41467-022-30335-2

[174] Lin, J.Y., de Sterke, C.M., Wheatland, M.S., Song, A.Y., Kuhlmey, B.T.: All-optical damping forces enhanced by metasurfaces for stable relativistic lightsail propulsion. Phys. Rev. Appl. **22**(6), 064028 (2024) https://doi.org/10.1103/PhysRevApplied.22.064028

[175] Manchester, Z., Loeb, A.: Stability of a light sail riding on a laser beam. Astrophys. J. Lett. **837**, 20 (2017) https://doi.org/10.3847/2041-8213/aa619b

[176] Ilic, O., Atwater, H.A.: Self-stabilizing photonic levitation and propulsion of nanostructured macroscopic objects. Nature Photonics **13**(4), 289–295 (2019) https://doi.org/10.1038/s41566-019-0373-y

[177] Srivastava, P.R., Chu, Y.-J.L., Swartzlander, G.A.: Stable diffractive beam rider. Opt. Lett. **44**(12), 3082–3085 (2019) https://doi.org/10.1364/OL.44.003082

[178] Srivastava, P.R., Swartzlander, G.A.: Optomechanics of a stable diffractive axicon light sail. Eur. Phys. J. Plus **135**(7), 570 (2020) https://doi.org/10.1140/epjp/s13360-020-00542-1

[179] Taghavi, M., Salary, M.M., Mosallaei, H.: Multifunctional metasails for self-stabilized beam-riding and optical communication. Nanoscale Adv. **4**(7), 1727–1740 (2022) https://doi.org/10.1039/D1NA00747E

[180] Santi, G., Corso, A.J., Garoli, D., Lio, G.E., Manente, M., Favaro, G., Bazzan, M., Piotto, G., Andriolli, N., Strambini, L., Pavarin, D., Badia, L., Proietti Zaccaria, R., Lubin, P., Ragazzoni, R., Pelizzo, M.G.: Swarm of lightsail nanosatellites for Solar System exploration. Scientific Reports **13**(1), 19583 (2023) https://doi.org/10.1038/s41598-023-46101-3

[181] Weingartner, J.C., Draine, B.T.: Dust grain-size distributions and extinction in the Milky Way, Large Magellanic Cloud, and Small Magellanic Cloud. The Astrophysical Journal **548**(1), 296–309 (2001) https://doi.org/10.1086/318651

[182] Early, J.T., London, R.A.: Dust grain damage to interstellar vehicles and lightsails. J. Br. Interplanet. Soc. **68**, 205–210 (2015)

[183] Hoang, T., Lazarian, A., Burkhart, B., Loeb, A.: The interaction of relativistic spacecrafts with the interstellar medium. Astrophys. J. **837**(1), 5 (2017) https://doi.org/10.3847/1538-4357/aa5da6

[184] Hoang, T., Loeb, A.: Electromagnetic forces on a relativistic spacecraft in the interstellar medium. Astrophys. J. **848**(1), 31 (2017) https://doi.org/10.3847/1538-4357/aa8c73

[185] Forward, R.L.: Roundtrip interstellar travel using laser-pushed lightsails. J. Spacecr. Rockets **21**(2), 187–195 (1984) https://doi.org/10.2514/3.8632

[186] Heller, R., Hippke, M., Kervella, P.: Optimized trajectories to the nearest stars using lightweight high-velocity photon sails. The Astronomical Journal **154**(3), 115 (2017) https://doi.org/10.3847/1538-3881/aa813f

[187] Minton, T.K., Wu, B., Zhang, J., Lindholm, N.F., Abdulagatov, A.I., O'Patchen, J., George, S.M., Groner, M.D.: Protecting polymers in space with atomic layer deposition coatings. ACS Applied Materials & Interfaces **2**(9), 2515–2520 (2010) https://doi.org/10.1021/am100217m

[188] Ádám, P., Dudás, L., Temesi, O., Nagy, A., Sinkó, K.: Porous aluminum oxide insulation materials tested in space mission. CEAS Space Journal **15**(5), 671–680 (2023) https://doi.org/10.1007/s12567-022-00473-w

[189] Fan, X., Shi, Y., Cui, M., Ren, S., Wang, H., Pu, J.: MoS_2/WS_2 nanosheet-based composite films irradiated by atomic oxygen: Implications for lubrication in space. ACS Applied Nano Materials **4**(10), 10307–10320 (2021) https://doi.org/10.1021/acsanm.1c01816

[190] Johnson, D.A., Gori, M., Vellore, A., Clough, A.J., Sitzman, S.D., Lince, J.R., Martini, A.: Evaluation of a commercial MoS_2 dry film lubricant for space applications. Lubricants **12**(9), 307 (2024) https://doi.org/10.3390/lubricants12090307

[191] Ismael, T., Abbas, M.A., Harris, O.P., Ingrish, G.B., Bush, M.E., Sasson, J.M., McNatt, J.S., Escarra, M.D.: High-specific power flexible photovoltaics from large-area MoS_2 for space applications. ACS Applied Energy Materials **8**(1), 87–98 (2025) https://doi.org/10.1021/acsaem.4c01797

[192] Lan, E.H., Smith, C.A., Cross, J.B.: Atomic oxygen effects on candidate coatings for long-term spacecraft in low Earth orbit. Technical Report 19890003221, NASA, Goddard Space Flight Center (January 1 1988). URL: https://ntrs.nasa.gov/citations/19890003221

[193] Bin-zhi, J., Yang, X., Chen, M., Zhi-hang, P., Jin, W., Jian-ping, C., Hai-long, L.: Space environment properties of Al_2O_3f/Al_2O_3 composites. Ceramics International **52**(3), 3470–3480 (2026) https://doi.org/10.1016/j.ceramint.2025.12.138

[194] Gao, X., Hu, M., Fu, Y., Weng, L., Liu, W., Sun, J.: MoS_2-Au/Au multilayer lubrication film with better resistance to space environment. Journal of Alloys and Compounds **815**, 152483 (2020) https://doi.org/10.1016/j.jallcom.2019.152483

[195] He, J., Wang, D., Hu, M., Gao, X., Sun, J., Li, Z., Lv, H., Guo, L., Fu, Y., Liu, Z., Jiang, D., Wang, Q., Yang, J., Zhao, X., Weng, L.: Unveiling the degradation mechanisms of MoS_2 lubricating films in authentic low earth orbit *via* China space station exposure experiment. Advanced Functional Materials **36**(10), 23156 (2026) https://doi.org/10.1002/adfm.202523156

[196] Nicaise, S.M., Lin, C., Azadi, M., Bozorg-Grayeli, T., Adebayo-Ige, P., Lilley, D.E., Pfitzer, Y., Cha, W., Van Houten, K., Melosh, N.A., Howe, R.T., Schwede, J.W., Bargatin, I.: Micron-gap spacers with ultrahigh thermal resistance and mechanical robustness for direct energy conversion. Microsyst. Nanoeng. **5**(1), 31 (2019) https://doi.org/10.1038/s41378-019-0071-4

[197] Campbell, M.F., Azadi, M., Lu, Z., Eskenazi, A.G., Jain, A., Bang, J.W., Sieg, P.G., Popov, G.A., Nicaise, S.M., Van Houten, K.C., Schmitt, F., Schwede, J.W., Bargatin, I.: Nanostructured spacers for thermionic and thermophotovoltaic energy converters. J. Microelectromech. Syst. **29**(5), 637–644 (2020) https://doi.org/10.1109/JMEMS.2020.3000422

[198] Ahn, S.H., Guo, L.J.: High-speed roll-to-roll nanoimprint lithography on flexible plastic substrates. Advanced Materials **20**(11), 2044–2049 (2008) https://doi.org/10.1002/adma.200702650

[199] Velten, T., Bauerfeld, F., Schuck, H., Scherbaum, S., Landesberger, C., Bock, K.: Roll-to-roll hot embossing of microstructures. Microsystem Technologies **17**(4), 619–627 (2011) https://doi.org/10.1007/s00542-010-1158-x

[200] Kooy, N., Mohamed, K., Pin, L.T., Guan, O.S.: A review of roll-to-roll nanoimprint lithography. Nanoscale Research Letters **9**(1), 320 (2014) https://doi.org/10.1186/1556-276X-9-320

[201] Peng, L., Wu, H., Shu, Y., Yi, P., Deng, Y., Lai, X.: Roll-to-roll hot embossing system with shape preserving mechanism for the large-area fabrication of microstructures. Review of Scientific Instruments **87**(10), 105120 (2016) https://doi.org/10.1063/1.4963907

[202] Haponow, L., Kettle, J., Allsop, J.: Optimization of a continuous hot embossing process for fabrication of micropyramid structures in thermoplastic sheets. Journal of Vacuum Science & Technology B **39**(1), 012203 (2020) https://doi.org/10.1116/6.0000551

[203] Di Russo, E., Tonon, A., Mischianti, A., Sgarbossa, F., Coleman, E., Gity, F., Panarella, L., Sheehan, B., Lebedev, V.A., De Salvador, D., Duffy, R., Napolitani, E.: Synthesis of large-area crystalline MoS_2 by sputter deposition and pulsed laser annealing. ACS Applied Electronic Materials **5**(5), 2862–2875 (2023) https://doi.org/10.1021/acsaelm.3c00362

[204] Lippman, M.E.: In-space fabrication of thin-film structures. Technical Report NASA-CR-1969, Astro Research Corporation, Santa Barbara, CA, USA (February 1972). URL: https://ntrs.nasa.gov/api/citations/19720009888/downloads/19720009888.pdf

[205] Gupta, B., Hossain, M.A., Riaz, A., Sharma, A., Zhang, D., Tan, H.H., Jagadish, C., Catchpole, K., Hoex, B., Karuturi, S.: Recent advances in materials design using atomic layer deposition for energy applications. Advanced Functional Materials **32**(3), 2109105 (2022) https://doi.org/10.1002/adfm.202109105

[206] Shih, B.-J., Chen, Z.-Y., Chang, S.-P., Chen, T.-Y., Sung, P.-J., Lin, N.-C., Yang, C.-C., Huang, P.-T., Cheng, H.-C., Li, M.-Y., Radu, I.P., Chen, K.-N.: Surface characteristics optimization during wafer-level backside silicon removal for SOI wafers in 3D integration. Applied Surface Science **688**, 162366 (2025) https://doi.org/10.1016/j.apsusc.2025.162366

[207] Kang, J.H., Gordon, K.L., Bryant, R.G., Stohlman, O.R., Wilkie, W.K., Stark, A.E., Barfield, R.S., Sindle, B.R., Finckenor, M.M., Craven, P.D.: Durability characterization of mechanical interfaces

in solar sail membrane structures. Advances in Space Research **67**(9), 2643–2654 (2021) https://doi.org/10.1016/j.asr.2020.08.015

[208] Penilla, E.H., Devia-Cruz, L.F., Wieg, A.T., Martinez-Torres, P., Cuando-Espitia, N., Sellappan, P., Kodera, Y., Aguilar, G., Garay, J.E.: Ultrafast laser welding of ceramics. Science **365**(6455), 803–808 (2019) https://doi.org/10.1126/science.aaw6699
