# Supplementary material for: Experimental demonstration of corrugated nanolaminate films as reflective light sails
Source: Nat Commun. 2026 May 26;17:6874. doi: 10.1038/s41467-026-73414-4 (PMC13389247; doi:10.1038/s41467-026-73414-4)
Supplement: Supplementary file 2 — Transparent Peer Review file [file 41467_2026_73414_MOESM2_ESM.pdf]

nature portfolio

Peer Review File

Experimental demonstration of corrugated nanolaminate films as reflective light sails

Corresponding Author: Professor Deep Jariwala

This file contains all reviewer reports in order by version, followed by all author rebuttals in order by version.

Attachments originally included by the reviewers as part of their assessment can be found at the end of this file.

Version 0:

Reviewer comments:

Reviewer #1

(Remarks to the Author)
This manuscript reports a novel corrugated nanolaminate film designed for reflective light sails. The work presents impressive experimental results and a comprehensive investigation of the material system and its potential suitability for light sail applications. Overall, the study is of high quality and addresses a topic of significant interest. In the reviewer's opinion, the manuscript is suitable for publication in a high-impact journal after addressing several minor comments listed below.
• Several comments relate to the discussion of thermal management, particularly infrared (IR) emissivity. The manuscript currently does not provide sufficient justification for the choice of Al_2O_3 thickness from the perspective of infrared emissivity considerations.
• At ~1000 K, the blackbody radiation peak shifts toward shorter wavelengths. In this regime, Al_2O_3 may not be an optimal material since its primary absorption lies above ~6 µm. The experimental study itself is already very comprehensive; however, it would be helpful if the authors could acknowledge this limitation of Al_2O_3 and briefly mention other potential materials (e.g., ZnO or Al:ZnO) that could be considered for improved mid-IR emissivity.
• No infrared reflection or absorption measurements appear to have been performed, and the IR emissivity is estimated using the transfer-matrix method. At minimum, it would be beneficial to include the simulated or calculated infrared spectrum in the Supplementary Information.
• For the comparison presented in Figure 4, adding a table in the Supplementary Information would allow a clearer and more quantitative comparison between the proposed structure and previously reported solutions.
• As noted in the discussion, thermal management constraints have not yet been fully considered. It would be valuable if the authors could comment on the potential technical limits of the proposed solution in terms of achievable mass density. Presumably, optimization of the layer thickness will play a key role. A brief discussion of the expected limits would strengthen the manuscript.
• For the photoresist stripping step, a two-step process (solvent sonication followed by O_2 plasma ashing) was used. Could the authors clarify the main limitation if only O_2 plasma treatment were applied? Would incomplete removal be expected?
• For removal of the Si substrate, laser ablation was employed. Could the authors comment on whether this step could alternatively be implemented using a DRIE process?

(Remarks on code availability)

Reviewer #2

(Remarks to the Author)
This work proposes a candidate lightsail structure with optimized reflectivity, area-to-mass ratio, emissivity and mechanical properties. Proposals targeting these aspects have been presented in past work (and acknowledged in this paper), but the breadth of analysis presented in this study goes beyond that of prior literature, especially within the substantial supplementary material, making it an impactful study. In particular, the design consisting of three layers (Al2O3, MoS2, Al2O3) has its main novelty in being shaped into corrugated hexagonal repeating cells possessing advantageous mechanical properties. The authors fabricate the proposed structure, test its composition and measure optical properties

such as reflection and transmission. Then, they compare their design against others in the literature using several metrics such as average reflectivity, emissivity-absorptivity ratio and areal density.

The paper is well composed, consistent with the literature and of great interest to the lightsail community. Given the breadth of the paper under study, this review focuses on aspects aligning with the reviewers' expertise, namely, lightsails and optical properties rather than the mechanical properties or material characterization.

Overall, this reviewer finds the paper is worthy of, and well suited for publication in Nature Communications. However, some points (numbered below) should be addressed:

References regarding the lightsail mission should include the latest book on the topic: [Laser Propulsion in Space, edited by C. Phipps (Elsevier, 2024)]

In Lines 59—63, the authors claim that previous lightsail designs struggled to address all the sail-performance requirements simultaneously while also citing Ref. 29 (Norder et al. 2025). Given that the photonic crystal from Ref. 29 appears to be better than the current work in 4 out of 6 metrics (according to Fig. 4), I suggest softening the wording or highlighting Ref. 29 as an exception.

The claim of broadband absorptivity being consistent with zero (<0.04) within the measurement uncertainty in Lines 182—184 is somewhat exaggerated given that kappa was measured to be 0.03 in supplementary Fig. S17 (without stating a large uncertainty). The measured kappa would be more consistent with an absorption around 1%, not 0.

All optical simulations appear to ignore the influence of the hexagonal corrugation. Given that aspects such as sail beam-riding stability are strongly influenced by non-specular scattering, the authors should comment on the scattering impact (or lack thereof) due to the corrugations if the entire structure were illuminated (as it would be in the mission). If the impact is non-negligible, the authors should comment on whether the design could be compatible with beam-riding stability requirements in photonic crystals, citing the relevant literature (e.g. [O. Ilic and H. A. Atwater, Nature Photonics 13, 289 (2019)], which was the first to propose nanostructuring for beam-riding stability and [J. Y. Lin, C. M. de Sterke, M. S. Wheatland, A. Y. Song, and B. T. Kuhlmey, Physical Review Applied 22, 064028 (2024)], which is a more recent example of gratings used to address the beam-riding stability problem).

The description in Fig. 3c “Shaded areas for $\varrho_{\lambda,\perp}$ and $\tau_{\lambda,\perp}$ reflect a 3% uncertainty along with the maximum variation among three sets of measurements.” is unclear, as it suggests there are two distinct elements being represented (a 3% uncertainty and a maximum variation). The authors should clarify this wording.

Throughout the main text and supplementary information, the reflectivity is said to be averaged over the Doppler-band associated with acceleration up to 0.2c. However, Ref. 132 (Chang et al. 2024) reports measured reflection spectra over a wavelength range (1300-1550 nm) that is about 13% shorter than the Doppler band (other references may be the same). This should be checked and mentioned in the supplementary information (similar to what was said in Supplementary S14.9 Lines 37—40).

Since large-area nanopatterning is a key design issue for lightsails (and considering the recent result of Norder et al. 2025 with the 60x60 mm^2 photonic crystal), the authors should briefly discuss the scalability of the fabricated nanolaminate structure in this work (or move some of the discussion from Supplementary S18 to the main text).

The optical measurements are done with a numerical aperture of 0.4, so that a wider range of angles than normal incidence are probed. Within the supplementary material, the authors should mention how this is addressed.

It is difficult to determine what is being shown in Figure S2a because it is almost entirely uniform in colour - is that an error, and if not, what information does the figure communicate?

(Remarks on code availability)

Reviewer #3

(Remarks to the Author)
I co-reviewed this manuscript with one of the reviewers who provided the listed reports. This is part of the Nature Communications initiative to facilitate training in peer review and to provide appropriate recognition for Early Career Researchers who co-review manuscripts.

(Remarks on code availability)

Reviewer #4

(Remarks to the Author)

[Editorial Note: Please also see attachment at end of file]

Dear authors:
This manuscript presents an interesting experimental demonstration of corrugated nanolaminate structures as candidate materials for laser-driven lightsails. I find the work technically sound and potentially suitable for publication after clarification of several points. My detailed assessment and specific questions are provided in the attached PDF review report.

(Remarks on code availability)
I have briefly checked the provided GitHub repository. The repository appears to include relevant datasets and scripts used in the study, along with a README file that provides basic documentation. The structure of the repository and the accompanying files seem reasonably organized and should allow interested readers to understand the computational and data analysis procedures used in the manuscript. While I did not attempt to fully run the code, the provided materials appear sufficient in principle to support reproducibility of the reported results.

Version 1:

Reviewer comments:

Reviewer #1

(Remarks to the Author)
The authors have made substantial efforts to revise and improve the manuscript, resulting in significant enhancements in both quality and clarity. The study is now presented to a high standard and is supported by comprehensive supplementary information. I therefore support its publication in Nature Communications.

(Remarks on code availability)

Reviewer #2

(Remarks to the Author)
We thank the authors for the many clarifications in the revision, which substantially improved the manuscript and supplementary material.
We don't entirely agree with the analysis of the optical scattering due to the hexagonal corrugation (since the periodicity leading to coherent backscattering effects that could be quite different from the cross sectional analysis provided) - but we can agree that this will require further, separate study as future work. In that sense, we believe the manuscript can be published without further revision.

(Remarks on code availability)

Reviewer #3

(Remarks to the Author)
I co-reviewed this manuscript with one of the reviewers who provided the listed reports. This is part of the Nature Communications initiative to facilitate training in peer review and to provide appropriate recognition for Early Career Researchers who co-review manuscripts.

(Remarks on code availability)

Reviewer #4

(Remarks to the Author)
Dear authors,

The revised manuscript has addressed the main concerns raised in the initial review. The additional analyses and newly included supplementary materials improve the clarity and completeness of the work. The revised version provides a clearer justification of the structural design choices, and the discussion of system stability has been strengthened. Optical response, including scattering-related discussions, is now more clearly presented and better supported.

Overall, the revisions have improved the rigor and transparency of the manuscript, and the main issues identified in the previous review have been satisfactorily resolved.

(Remarks on code availability)
The code is well organized.

Open Access This Peer Review File is licensed under a Creative Commons Attribution 4.0 International License, which permits use, sharing, adaptation, distribution and reproduction in any medium or format, as long as you give appropriate credit to the original author(s) and the source, provide a link to the Creative Commons license, and indicate if changes were made.
In cases where reviewers are anonymous, credit should be given to 'Anonymous Referee' and the source.
The images or other third party material in this Peer Review File are included in the article's Creative Commons license, unless indicated otherwise in a credit line to the material. If material is not included in the article's Creative Commons license and your intended use is not permitted by statutory regulation or exceeds the permitted use, you will need to obtain permission directly from the copyright holder.
To view a copy of this license, visit https://creativecommons.org/licenses/by/4.0/

Author's Response for NCOMMS-26-015816:
Experimental demonstration of corrugated nanolaminate films as reflective light sails

We thank the referees for their insightful comments, and we have implemented the suggested changes. We provide a brief summary of the most important alterations at the top of this letter, and a detailed response to the questions in subsequent pages. Also, the changes are highlighted in the modified manuscript and Supplemental Information documents.

Brief summary

- Main text (MT): We removed the "absorption consistent with zero" wording, added information about the laser measurements, and added several clarifying references to material in the Supplementary Information.
- Supplemental Information (SI): We added two panels to Figure S14 and Figure S15 about the mechanical benefits of the hexagonal corrugation; added Figures S27 and S29, which show calculations of both the angle-dependent reflectivity and absorptivity for the fabricated prototype and optimized designs, respectively; and added Figure S28, which shows calculations of the spectral hemispherical emissivity for the fabricated prototype and optimized designs.
- SI: We added Section S18, which discusses scattering by the hexagonal corrugation; added Section S19, which discusses beam-riding stability; and added Section S20, which discusses the in-space robustness of the sail.
- SI: In Section S9, we discussed the impact of the $NA = 0.4$ numerical aperture; in Section S10, we estimated the minimum achievable film areal density under thermal constraints; in Section S14.8, we clarified our calculation of the optical properties of the prototype from Chang *et al.* (2024) [1]; and in Section S21, we added more information about scalability.

Reviewer #1

1. *This manuscript reports a novel corrugated nanolaminate film designed for reflective light sails. The work presents impressive experimental results and a comprehensive investigation of the material system and its*

potential suitability for light sail applications. Overall, the study is of high quality and addresses a topic of significant interest. In the reviewer's opinion, the manuscript is suitable for publication in a high-impact journal after addressing several minor comments listed below.

- We thank Reviewer #1 for these comments and for their suggested edits.

2. *Several comments relate to the discussion of thermal management, particularly infrared (IR) emissivity. The manuscript currently does not provide sufficient justification for the choice of Al_2O_3 thickness from the perspective of infrared emissivity considerations.*

   - We thank Reviewer #1 for this comment about our film optimization procedure. We chose to optimize our film for β_{max}, which resulted in the Al_2O_3 and MoS_2 thicknesses provided. Thicker Al_2O_3 face films would likely be required in a design optimized for a thermal target, such as the film's effective emissivity ε_e. We added a sentence about this to the Supplementary Information, and also added a parenthetical comment in the main text pointing readers to Section S13 for more details on the film optimization procedure. In addition, to provide more information about the emissivity of the fabricated prototype and proposed optimized films, we added Figure S28 to the Supplementary Information, which shows the films' spectral hemispherical emissivity spectra.
   - MT: . . . within the design space (see Methods and Section S13 in the Supplementary Information).
   - SI: Alternatively, a different design could be optimized for its effective emissivity ε_e (Equation S82) rather than for β_{max}.
   - SI: For reference, our calculations of the film's spectral hemispherical emissivity (Equation S82) are provided in Figure S28.

3. *At* ~ 1000 K, *the blackbody radiation peak shifts toward shorter wavelengths. In this regime, Al_2O_3 may not be an optimal material since its primary absorption lies above* ~ 6 μm. *The experimental study itself is already very comprehensive; however, it would be helpful if the authors could acknowledge this limitation of Al_2O_3 and briefly mention other potential materials* (e.g., *ZnO or Al:ZnO) that could be considered for improved mid-IR emissivity.*

   - We thank Reviewer #1 for highlighting this important fact about Al_2O_3. We had originally made a brief comment about the wavelength mismatch in the caption of Figure S21, but, to emphasize it more, have now commented about this in the text of Section S8. In addition, in both the main article text and Supplementary Information, we added a note about Al:ZnO as an alternative material system that may have better emissive performance.
   - MT & SI: . . . different face sheet materials such as SiO_2 [2], Si_3N_4 [3, 4], or (Al:)ZnO [5].
   - SI: Although the peaks at higher temperatures correspond to wavelengths where the extinction coefficient of Al_2O_3 is not as large, the overall exitance can still be significant because the peak of the black-body radiation curve increases dramatically in magnitude. See Section S17 for additional information on thermal considerations for the light sails.

4. *No infrared reflection or absorption measurements appear to have been performed, and the IR emissivity is estimated using the transfer-matrix method. At minimum, it would be beneficial to include the simulated or calculated infrared spectrum in the Supplementary Information.*

   - We appreciate Reviewer #1's helpful suggestion. We have added Figure S28 to the Supplementary Information, which provides the calculated spectral hemispherical emissivity spectra of the fabricated prototype and proposed optimized films.
   - SI: (caption) Calculations of the spectral hemispherical emissivity of the fabricated prototype and proposed optimized films. See Equation S82.

5. *For the comparison presented in Figure 4, adding a table in the Supplementary Information would allow a clearer and more quantitative comparison between the proposed structure and previously reported solutions.*

   - We agree with Reviewer #1 that a companion table of values corresponding to Figure 4 would be greatly beneficial. To aid readers, in the main text, we added specific call-outs to the table in the Supplementary Information.

- MT: . . . see Methods and Section S14 of the Supplementary Information, including Table S2.
- MT: . . . and areal density values (see also Table S2 in the Supplementary Information).

6. *As noted in the discussion, thermal management constraints have not yet been fully considered. It would be valuable if the authors could comment on the potential technical limits of the proposed solution in terms of achievable mass density. Presumably, optimization of the layer thickness will play a key role. A brief discussion of the expected limits would strengthen the manuscript.*
   - We appreciate Reviewer #1's question about how thermal considerations place a lower bound on the sail's areal density, because it highlights the effects of film layer thickness on absorptivity and emissivity. We added a brief discussion to Section S10 of the Supplementary Information.
   - SI: We can use the emissivity-to-absorptivity ratio $\frac{\varepsilon_e}{\alpha_\perp} \approx 10^6$ to estimate the minimum areal density achievable for our class of nanolaminate sails when optimizing solely for thermal considerations. We begin by using Equation S80 to estimate the absorptivity and emissivity (by Kirchhoff's law of thermal radiation) as a function of the film thickness.

$$\frac{\varepsilon_e}{\alpha_\perp} \sim \frac{\frac{4\pi}{\lambda_{radiate}}\left(2\kappa_{A,radiate}t_{f,A}F_A + \kappa_{M,radiate}t_{f,M}F_M\right)}{\frac{4\pi}{\lambda_{absorb}}\left(2\kappa_{A,absorb}t_{f,A}F_A + \kappa_{M,absorb}t_{f,M}F_M\right)} \tag{1}$$

Here, subscripts A and M represent Al_2O_3 and MoS_2, respectively; $\lambda_{radiate}$ and λ_{absorb} are characteristic wavelengths for radiation (*e.g.*, in the infrared) and absorption (*e.g.*, the laser wavelength), respectively; $\kappa_{radiate}$ and κ_{absorb} are the characteristic extinction coefficients for radiation and absorption, respectively; t_f is the film thickness; F is a layer fill factor; and for simplicity we assume the top and bottom alumina layers have the same thickness. Rearranging, letting $F_A = F_M = 1$ (*i.e.*, complete and uniform material coverage with no holes), and solving for the film thickness ratio $\frac{t_{f,M}}{t_{f,A}}$ gives

$$\frac{t_{f,M}}{t_{f,A}} \sim \frac{2\frac{\varepsilon_e}{\alpha_\perp}\frac{\lambda_{radiate}}{\lambda_{absorb}}\kappa_{A,absorb} - 2\kappa_{A,radiate}}{\kappa_{M,radiate} - \frac{\varepsilon_e}{\alpha_\perp}\frac{\lambda_{radiate}}{\lambda_{absorb}}\kappa_{M,absorb}}. \tag{2}$$

The equation only has meaningful solutions for $\frac{t_{f,M}}{t_{f,A}} > 0$, which places constraints on the optical values selected. To find the minimum possible areal density under thermal constraints we select optimistic values (see Section S15) of $\kappa_{A,absorb} = \kappa_{M,absorb} = \kappa_{A,radiate} = 10^{-8}$ (the latter because we have not observed emissive features in MoS_2 at longer wavelengths) and $\kappa_{A,radiate} = 10^{-1}$, and select $\lambda_{absorb} = \lambda_l = 1.2$ µm and $\lambda_{radiate} = 2.9$ µm (the peak of the blackbody spectrum at $T = 1000$ K according to Wien's displacement law [6]). Using these values, we find $\frac{t_{f,M}}{t_{f,A}} \sim 6.3$. A minimum achievable thickness of Al_2O_3 on MoS_2 by atomic layer deposition is roughly $t_{f,A} \sim 1.5$ nm [7], leading to a minimum MoS_2 thickness of $t_{f,M} \sim 9.5$ nm. Finally, noting that the lowest-areal-density sails will occupy the limit where the rib/trench height is zero, these thicknesses (one MoS_2 sheet with two Al_2O_3 face sheets) lead to $\rho_{a,minimum} \sim 0.058$ g m^{-2} (Equation S2). This limit is well within the $\rho_a \sim 0.1$ g m^{-2} goal set by the Breakthrough Starshot Foundation [8–11].

7. *For the photoresist stripping step, a two-step process (solvent sonication followed by O_2 plasma ashing) was used. Could the authors clarify the main limitation if only O_2 plasma treatment were applied? Would incomplete removal be expected?*
   - We thank Reviewer #1 for this significant fabrication question, as it is of the utmost importance that the Si substrate be perfectly clean prior to depositing the first Al_2O_3 layer. It is indeed possible to remove photoresist using O_2 plasma only [12] but we did not pursue this approach. We have added a note about this to the manuscript.
   - MT: . . . longer quiescent soak (an alternative could also be to forgo the liquid-cleaning step altogether [12]).

8. *For removal of the Si substrate, laser ablation was employed. Could the authors comment on whether this step could alternatively be implemented using a DRIE process?*

   - We thank Reviewer #1 for pointing out the alternate technique of using deep reactive ion etching (DRIE) to remove the wafer backside Si substrate, as it is widely used in the semiconductor industry [13]. We did not attempt it, in part because it would have required additional protection/coating and subsequent cleaning steps on the corrugated film sides of our chips. However, this strategy would be a good possibility for future fabrication iterations. Were DRIE to be used, it would be important to consider the topographical shape of the remaining Si; we found that removing more Si from the center of the membrane and less at the edges allowed the center to released first in the ultimate XeF_2 process, mitigating film tears. We have added a note about this to the manuscript.
   - MT: . . . in the ablation region (note that backside DRIE could potentially be used instead of laser ablation [13]).

Reviewer #2

1. *This work proposes a candidate lightsail structure with optimized reflectivity, area-to-mass ratio, emissivity and mechanical properties. Proposals targeting these aspects have been presented in past work (and acknowledged in this paper), but the breadth of analysis presented in this study goes beyond that of prior literature, especially within the substantial supplementary material, making it an impactful study. In particular, the design consisting of three layers (Al_2O_3, MoS_2, Al_2O_3) has its main novelty in being shaped into corrugated hexagonal repeating cells possessing advantageous mechanical properties. The authors fabricate the proposed structure, test its composition and measure optical properties such as reflection and transmission. Then, they compare their design against others in the literature using several metrics such as average reflectivity, emissivity-absorptivity ratio and areal density. The paper is well composed, consistent with the literature and of great interest to the lightsail community. Given the breadth of the paper under study, this review focuses on aspects aligning with the reviewers' expertise, namely, lightsails and optical properties rather than the mechanical properties or material characterization. Overall, this reviewer finds the paper is worthy of, and well suited for publication in Nature Communications. However, some points (numbered below) should be addressed:*

   - We thank Reviewer #2 for these comments and for their suggested edits.

2. *References regarding the lightsail mission should include the latest book on the topic: [Laser Propulsion in Space, edited by C. Phipps (Elsevier, 2024)] [11]*

   - We thank Reviewer #2 for bringing this book to our attention, and have added the requested reference in the main text.
   - MT: . . . highly reflective, ultra-lightweight membranes that accelerate miniature chip satellites to relativistic velocities [11].
   - MT: . . . within an order of magnitude of the target set by the Breakthrough Starshot Foundation [11].

3. *In Lines 59—63, the authors claim that previous lightsail designs struggled to address all the sail-performance requirements simultaneously while also citing Ref. 29 (Norder* et al. *2025 [14]). Given that the photonic crystal from Ref. 29 appears to be better than the current work in 4 out of 6 metrics (according to Fig. 4), I suggest softening the wording or highlighting Ref. 29 as an exception.*

   - We thank Reviewer #2 for pointing this out; it is true that, according to Figure 4, other sail designs perform better than ours in several categories concurrently. In our view, the important task of a light sail design is to balance the tradeoffs between all of these metrics in order to achieve the best overall performance. In fact, the "best" sail overall may not be the best by *any* single measure used. Although our fabricated prototype and proposed optimized designs do not stand out by individual standards, we feel that their overall, holistic performance is noteworthy. We have reworded this sentence to better reflect our perspective.
   - MT: Many previous designs, though maximizing reflectivity or minimizing areal density, struggled to manage the tradeoffs between all of these requirements simultaneously.

4. *The claim of broadband absorptivity being consistent with zero (<0.04) within the measurement uncertainty in Lines 182—184 is somewhat exaggerated given that kappa was measured to be 0.03 in supplementary Fig. S17 (without stating a large uncertainty). The measured kappa would be more consistent with an absorption around 1%, not 0.*

   - We appreciate Reviewer #2 bringing this to our attention, since the wording "consistent with zero" is precise and implies a statistical significance. In addition, Reviewer #2 rightly pointed out that we did not include uncertainty estimates for the index of refraction data included in Figure 2(b) (also in Figure S17). To address this comment, we altered the wording in the abstract and the main text to remove the "consistent with zero" phrase. Also, we made a note about the uncertainty of the n and κ data in the caption of Figure 2. Moreover, we agree with Reviewer #2 that our measurement of the extinction coefficient of MoS_2 (Figure 2(b)) suggests a film absorptivity greater than zero, as supported by the simulations shown in Figure 3(c). We wish to emphasize that, although the actual prototype film we fabricated exhibits too much absorption to be viable, it represents a larger class of sail films that our calculations indicate could achieve sufficiently low absorption to fly.
   - MT: . . . and feature broadband absorptivities of $< 4\%$ with a measurement uncertainty that overlaps with zero - indicative of our sail class's potential for fast acceleration and ultra-low photon absorption.
   - MT: Moreover, the broadband absorptivity of $\alpha_{\lambda,\perp} = 1 - \rho_{\lambda,\perp} - \tau_{\lambda,\perp} < 0.04$ has an associated uncertainty that overlaps with zero, suggesting the capacity of our class of films to achieve ultra-low photon absorption if optimized and fabricated with fewer imperfections. . . . Although our transfer-matrix method simulations suggest an absorptivity greater than zero, we emphasize that our experimental results highlight the strong potential of our nanolaminate design.
   - MT: (caption) The relative uncertainties of the n and κ data within the laser band are $\pm 0.8\%$ and $\pm 2.6\%$, respectively.

5. *All optical simulations appear to ignore the influence of the hexagonal corrugation. Given that aspects such as sail beam-riding stability are strongly influenced by non-specular scattering, the authors should comment on the scattering impact (or lack thereof) due to the corrugations if the entire structure were illuminated (as it would be in the mission). If the impact is non-negligible, the authors should comment on whether the design could be compatible with beam-riding stability requirements in photonic crystals, citing the relevant literature (e.g. [O. Ilic and H. A. Atwater, Nature Photonics 13, 289 (2019) [15]], which was the first to propose nanostructuring for beam-riding stability and [J. Y. Lin, C. M. de Sterke, M. S. Wheatland, A. Y. Song, and B. T. Kuhlmey, Physical Review Applied 22, 064028 (2024) [16]], which is a more recent example of gratings used to address the beam-riding stability problem).*

   - We thank Reviewer #2 for this insightful comment and for bringing these two additional articles to our attention. Scattering from the hexagonal edges is indeed possible. Fortunately, because the films are precisely microfabricated, we anticipate that this scattering may be predictable, which could allow metamaterial or diffractive surfaces to compensate if necessary. We have added a new section to the Supplementary Information (Section S19) to briefly discuss beam-riding stability and to provide additional references.
   - MT: . . . In addition, more work is needed to address concerns of beam-riding stability, which could be related to both the film's nano-architecture and its macroscopic shape [15, 16].
   - SI: Light sails must be designed to stay within the laser beam during the acceleration phase. For instance, if the sail shifts laterally away from the center of the laser beam or tilts so its heading is no longer aligned with the beam, the sail should feature automatic compensation mechanisms to bring it back into alignment. Methods for damping oscillations are also important [16]. The stabilization must generally be passive because feedback control on the laser beam would be difficult given the fast acceleration experienced by light sails. Leading strategies for sail stabilization include selecting macroscopic shapes such as spherical curves that inherently imply stability [17–19] or by leveraging nanostructured or diffractive surfaces that scatter light in favorable ways [15, 16, 20–28]. A detailed study of these methods is beyond the scope of this work, but we briefly summarize a few considerations for corrugated light sail films in particular. As mentioned in Section S18, some photons may be scattered by the corrugated structure of our

sail films. These scattered photons could have the potential to destabilize the sail, making it important to implement stabilizing techniques to compensate. A simple method could be to employ a spherically curved shape for the sail [17, 19]. In this aspect, hexagonal corrugation offers the benefit of increased bending stiffness relative to flat plates, thereby helping the film to maintain its curved shape despite the radiation pressure. In addition, metamaterial coatings could be applied to the horizontal elements in our films (*i.e.*, the hexagon tops and trench bottoms for films in the indented trench configuration). Presumably, if fabricated with sufficiently high precision, the films' microfabricated hexagonal architecture would give rise to predictable or repeatable scattering patterns, and the coatings could be designed with this in mind. Such features would need to be applied in radially-varying arrays to generate the correct effects in reflectivity magnitude and phase. These features could be patterned by growing them on top of the film (additive manufacturing) or by etching them into the Si mold prior to applying the first Al_2O_3 coating. Finally, as mentioned in Section S9 (see also Figure S24), we have experimented with adding a flat MoS_2 film on top of a corrugated Al_2O_3 frame. The upper flat surface could serve to prevent edge scattering.

6. *The description in Fig. 3c "Shaded areas for $\rho_{\lambda,\perp}$ and $\tau_{\lambda,\perp}$ reflect a 3% uncertainty along with the maximum variation among three sets of measurements." is unclear, as it suggests there are two distinct elements being represented (a 3% uncertainty and a maximum variation). The authors should clarify this wording.*
   - We appreciate that Reviewer #2 pointed out how this is confusing. To aid readers, in the caption of Figure 3 in the article, we added a specific reference to Section S9 in the Supplementary Information where we explain how we derived the shaded error regions. In addition, in the Supplementary Information, we added more details about the underlying causes of the 3% uncertainty.
   - MT: (see Section S9 of the Supplementary Information for a detailed description).
   - SI: This uncertainty is attributable to the mechanical instability of the monochromator, to mechanical and thermal drifts of components in the optical path, to non-perfect beam forming and illumination, to noise in the detection electronics, and the nature of non-single-shot reflection and transmission measurements.

7. *Throughout the main text and supplementary information, the reflectivity is said to be averaged over the Doppler-band associated with acceleration up to 0.2c. However, Ref. 132 (Chang* et al. *2024 [1]) reports measured reflection spectra over a wavelength range (1300-1550 nm) that is about 13% shorter than the Doppler band (other references may be the same). This should be checked and mentioned in the supplementary information (similar to what was said in Supplementary S14.9 Lines 37—40).*
   - We thank Reviewer #2 for pointing this out. Indeed, the laser band associated with the sail of Chang *et al.* 2024 [1] is $\lambda = \langle 1.300, 1.592 \rangle$ µm but the experimental data provided span the range $\lambda = \langle 1.309, 1.550 \rangle$ µm. In our calculations, we linearly extrapolated the measurements to achieve the entire range, which we believe to be reasonable given the trends shown in both the measurements and simulations in the article. We have augmented the text of Section S14.8 in the Supplementary Information to explain this. We also checked the other articles to ensure that their spectra comprised the correct wavelength ranges, and verified that the laser output wavelength was included as a detail in all of the descriptive sections.
   - SI: We estimated the absorptivity spectrum of their prototype by subtracting their corrected reflectivity (labeled "Cali. refl.: SiN PhC+Si" in Figure 4 of their article [1]) and transmissivity (labeled "Meas. trans.: SiN PhC+Si" in Figure 4 of their article [1]) spectra from unity, and subsequently calculated the laser band reflectivity $\overline{\rho_\perp}$ and absorptivity $\overline{\alpha_\perp}$ (Equations S78 and S81, respectively). Importantly, the optical measurements provided in the article span the wavelength range $\lambda = \langle 1.309, 1.550 \rangle$ µm, whereas the laser band required to reach $\beta = 0.2$ for the stated laser output wavelength is $\lambda = \langle 1.300, 1.592 \rangle$ µm. To achieve the full range in order to obtain $\overline{\rho_\perp}$ and $\overline{\alpha_\perp}$ and calculate β_{max}, we linearly extrapolated the provided reflectivity and transmissivity data in the unknown regions, which we believe to be reasonable given the trends shown in both their experimental and simulated results.

8. *Since large-area nanopatterning is a key design issue for lightsails (and considering the recent result of Norder* et al. *2025 [14] with the 60×60 mm^2 photonic crystal), the authors should briefly discuss*

the scalability of the fabricated nanolaminate structure in this work (or move some of the discussion from Supplementary S19 to the main text).

- We thank Reviewer #2 for this important critique. We have included a brief discussion of methods for scaling up our fabrication process in the main text and in the Methods section, as well as in Section S21 in the Supplementary Information. However, to aid readers, we also added a reference to Section S21 in the caption of Figure 3.
- MT: see Methods; for information on scaling this process to larger suspended sizes, see Section S21 in the Supplementary Information.

9. *The optical measurements are done with a numerical aperture of 0.4, so that a wider range of angles than normal incidence are probed. Within the supplementary material, the authors should mention how this is addressed.*

- We thank Reviewer #2 for pointing this out. Indeed, this numerical aperture ($NA = 0.4$) admits a cone of light within an incident angle (from the normal axis) of roughly 23.6°, such that measurements are not comprised strictly of normally-reflected photons. We conducted reflectivity and absorptivity simulations for both the fabricated prototype and proposed optimized designs at angles ranging from normal incidence to 80° off-axis at light wavelengths in the range $\langle 1 \le \lambda \le 1.6 \rangle$ µm (shown in Supplementary Information Figures S27 and S29). These simulations predict that the reflectivity and absorptivity of the fabricated prototype design vary by at most 1% and 1.2%, respectively, within the laser band ($\lambda = \langle 1.2, 1.4697 \rangle$ µm) and the $NA = 0.4$ angular range ($0 \le \theta \le 23.6°$). This suggests that we can regard the experimental reflectivity and absorptivity results as normal-incidence data. We have updated the main text and Supplemental Information Section S9 with details on the impact of the aperture.
- MT: Note that this optic collects photons at angles ranging from normal incidence to roughly 23.6°; we simulated the reflectivity and absorptivity within this range to confirm that the angular-dependence of these data is minimal (see Figure S27 in the Supplementary Information).
- SI: Note that this optic collects photons at angles ranging from normal incidence to roughly 23.6°. We conducted transfer-matrix method simulations using our measured indices of refraction and found that the reflectivity and absorptivity of the fabricated prototype design vary by at most 1% and 1.2%, respectively, within the laser band ($\lambda = \langle 1.2, 1.4697 \rangle$ µm) and the $NA = 0.4$ angular range ($0 \le \theta \le 23.6°$); see Figure S27. This suggests that the experimental results can be regarded as normal-incidence data.
- SI: (caption) Angular dependence of reflectivity and absorptivity for the fabricated prototype film. Contour plots provide simulations of the (**a**) reflectivity and (**b**) absorptivity for the fabricated prototype film, with thicknesses $t_{A,b} \approx 21$ nm (bottom Al_2O_3 thickness), $t_M \approx 53$ nm (MoS_2), and $t_{A,t} \approx 51$ nm (top Al_2O_3). The simulations do not account for the film's hexagonal corrugation. The vertical gray lines denote the Doppler-shifted wavelength range corresponding to a final relative velocity of $\beta_f = 0.2$: $\lambda = \langle 1.2, 1.4697 \rangle$ µm. The horizontal dashed line corresponds to the maximum angular range accepted by the $NA = 0.4$ numerical aperture in the laser experiments ($0 \le \theta \le 23.6°$; see Section S9). Simulations shown use measured indices of refraction for Al_2O_3 and MoS_2 (see Section S8).
- SI: (caption) Angular dependence of reflectivity and absorptivity for the proposed optimized film. Contour plots provide simulations of the (**a**) reflectivity and (**b**) absorptivity for the proposed optimized film, with thicknesses $t_{A,b} = t_{A,t} \approx 19$ nm and $t_M \approx 63$ nm. The simulations do not account for the film's hexagonal corrugation. The vertical gray lines denote the Doppler-shifted wavelength range corresponding to a final relative velocity of $\beta_f = 0.2$: $\lambda = \langle 1.2, 1.4697 \rangle$ µm. Simulations use literature index of refraction data [3, 29]. The discontinuity near $\lambda = 1.55$ is related to extrapolating the Kischkat *et al.* [3] data. See also Figure S27.

10. *It is difficult to determine what is being shown in Figure S2a because it is almost entirely uniform in colour - is that an error, and if not, what information does the figure communicate?*

- We thank Reviewer #2 for bringing this to our attention. We have enlarged the cropping on the figure to show the outline of the chip along with a ruler next to it, and have updated the caption with the new scale bar dimension accordingly.

- SI: Scale bars: (**a**) 10 mm, (**b**) 10 mm.

Reviewer #3

1. *I co-reviewed this manuscript with one of the reviewers who provided the listed reports. This is part of the Nature Communications initiative to facilitate training in peer review and to provide appropriate recognition for Early Career Researchers who co-review manuscripts.*
   - We thank Reviewer #3 for their service to the profession and for reading our manuscript.

Reviewer #4

1. *This manuscript presents an interesting experimental demonstration of corrugated nanolaminate structures as candidate materials for laser-driven lightsails. I find the work technically sound and potentially suitable for publication after clarification of several points. My detailed assessment and specific questions are provided in the attached PDF review report.*
   The manuscript by M.F. Campbell et al. *proposed a three-layer $Al_2O_3/MoS_2/Al_2O_3$ nanolaminate architecture combined with a hexagonally corrugated microstructure to simultaneously address the optical, mechanical, and mass constraints required for relativistic light-sail propulsion. The authors fabricate prototype films using semiconductor-compatible processes and characterize their structural and optical properties, reporting broadband reflectivity exceeding 50% and absorptivity below approximately 4% in the proposed laser band. This work also analyzes the potential propulsion performance and compares the proposed design with previously reported lightsail concepts. Overall, the manuscript is interesting and solid in terms of concept and methodology, and the idea of combining multilayer nanolaminate optics with architected corrugated mechanical structures is promising. I am generally supportive of publication if the authors could clarify several points and further discuss some aspects of the design motivation and optical implications of the system. See detailed questions below:*
   - We thank Reviewer #4 for these comments and for their suggested edits.
2. *The manuscript introduces a hexagonally corrugated architecture to enhance bending stiffness and mechanical robustness, but the motivation for choosing this corrugated geometry over a planar multilayer structure could be explained more clearly. In particular, it would be helpful if the authors could elaborate in the earlier part of the manuscript on why a corrugated architecture is preferred instead of a three-layer planar film with increased thickness or other mechanical reinforcement strategies. Quantitative comparisons between planar and corrugated configurations, for example in terms of stiffness-to-mass ratio or wrinkle suppression, would help justify the design choice.*
   - We appreciate Reviewer #4's comment, and agree that the text and figures could more clearly communicate the importance and advantages of corrugation. To address this comment, we slightly expanded the text introducing the film corrugation early in the main text, and added call-outs to the two relevant figures in the Supplemental Information (Figures S14 and S15). In both of these supplemental figures, we added two panels: one to show the ratio of the areal density of the corrugated film to that of a flat/planar film with the same nanolaminate thicknesses (the areal density ratio, ADR), and a second to show the ratio of the bending stiffness enhancement factor to the ADR. These figures illustrate the mass penalty associated with corrugation and the stiffness gain that corrugation brings relative to the associated mass penalty.
   - MT: The out-of-plane "U"-shapes of this pattern greatly increase the bending stiffness of the architected films relative to their non-corrugated counterparts (the bending stiffness of a beam scales with the cube of its effective thickness [30]), much like the vertical section of an "I"-beam increases its rigidity by increasing its bending moment of inertia [31] (see Figure 3(e) and Section S3 in the Supplementary Information).
   - MT: (see Figures S14 and S15 in the Supplementary Information)
   - SI: (caption) Mechanical property calculations for fabricated prototype film. Panel (**a**) shows the corrugated areal density $\rho_{a,c,t}$ for hexagonally corrugated films (indented trench configuration) with $t_{A,b} \approx 21$ nm (bottom Al_2O_3 thickness), $t_M \approx 53$ nm (MoS_2), and $t_{A,t} \approx 51$ nm (top

Al_2O_3), and panel (**b**) shows the areal density ratio (ADR): the ratio of the areal density of a corrugated film to that of a flat/planar film with the same nanolaminate thicknesses (areal density $\rho_{a,p} \approx 0.5\ \mathrm{g\,m^{-2}}$). Panel (**c**) shows the tensile stiffness reduction factor $\mathbb{T}$ and panel (**d**) provides the ratio of the bending stiffness enhancement factor $\mathbb{B}$ (Figure 3(e) in the main article) to the areal density ratio (panel (b) of this figure). The fabricated prototype design, featuring $d_h \approx 77$ µm, $w_t \approx 15$ µm, $h_t \approx 10$ µm, $\rho_{a,c,t} \approx 0.7\ \mathrm{g\,m^{-2}}$, $\mathbb{B} \approx 38$, and $\mathbb{T} \approx 2.4$, is shown with the white circle. In this case, the value $\mathbb{T} > 1$ indicates no reduction in the tensile stiffness relative to a planar (non-corrugated) film. See also Figure S15.

- SI: (caption) Mechanical property calculations for proposed optimized film. Panel (**a**) shows the corrugated areal density $\rho_{a,c,t}$ for hexagonally corrugated films (indented trench configuration) with $t_{A,b} = t_{A,t} \approx 19$ nm and $t_M \approx 63$ nm, and panel (**b**) shows the areal density ratio (ADR): the ratio of the areal density of a corrugated film to that of a flat/planar film with the same nanolaminate thicknesses (areal density $\rho_{a,p} \approx 0.4\ \mathrm{g\,m^{-2}}$). Panel (**c**) shows the tensile stiffness reduction factor $\mathbb{T}$ and panel (**d**) provides the ratio of the bending stiffness enhancement factor $\mathbb{B}$ (Figure 3(e) in the main article) to the areal density ratio (panel (b) of this figure). The proposed optimized design, featuring $d_h = 70$ µm, $w_t = 2$ µm, $h_t = 3$ µm, $\rho_{a,c,t} \approx 0.5\ \mathrm{g\,m^{-2}}$, $\mathbb{B} \approx 890$, and $\mathbb{T} \approx 0.0033$, is shown with the white triangle. See also Figure S14.

3. *While the mechanical advantages of the corrugated geometry are discussed in detail, the manuscript does not fully address how this out-of-plane structure may influence the optical performance. Because the architecture introduces edges, trenches, and nonplanar surfaces, it would be useful for the authors to comment on whether the corrugation could introduce additional scattering or reflection losses, and whether such effects were observable or negligible in the optical measurements presented in the main manuscript.*

- We appreciate Reviewer #4's concerns. Indeed, laser light may scatter from the three-dimensional architected features of our films. This could impact both the overall reflectivity (and hence acceleration) of our sail and its beam-riding stability. To address the impact on reflectivity, we added Section S18 to the Supplementary Information. There, we estimate the fractional area of the hexagonally-patterned film that may be impacted by scattering. For a discussion of the impact of corrugation on beam-riding stability, please see our response to Reviewer #2 Response 5 on page 5 of this letter, where we introduce Section S19 in the Supplementary Information. In our optical measurements we avoided scattering losses by focusing the laser beam to a 10 µm-diameter spot in the center of the hexagonal areas.

- SI: The hexagonal corrugation in our sails may cause some photons to scatter rather than be reflected specularly. We can conservatively estimate the fraction of incident light that will be affected by examining Figures S12 and S13. Each single hexagonal unit cell shown has an area given by Equations S7 or S8 for the indented trench and protruding rib configurations, respectively. Also, each unit cell comprises six hexagon sides with length a_h (Equation S1). The lateral width impacted by each vertical wall can be estimated as being roughly proportional to the depth of the trenches h_t or height of the ribs h_r. Thus, the total area impacted by scattering (henceforth written for the trench configuration) can be approximated by

$$A_{s,t} \approx 6 a_h h_t \tag{3}$$

Combining Equations S1, S7, and S147, we find the fraction of impacted area $F_{scatter}$ to be approximately

$$F_{scatter} \approx \frac{A_{s,t}}{A_{uc,t}} = \frac{6\frac{d_h}{\sqrt{3}} h_t}{\frac{\sqrt{3}}{2}(d_h + w_t)^2} = \frac{4 d_h h_t}{(d_h + w_t)^2}. \tag{4}$$

For the fabricated prototype design with $d_h \approx 77$ µm, $w_t \approx 15$ µm, and $h_t \approx 10$ µm, we find $F_{scatter} = 0.364$. For the proposed optimized design, with $d_h = 70$ µm, $w_t = 2$ µm, and $h_t = 3$ µm, we have $F_{scatter} = 0.162$. Note that these fractions are conservative estimates, and detailed numerical simulations would be necessary to more accurately quantify the scattering. Future work would benefit from co-optimizing the mechanical aspects of corrugated sails (bending stiffness enhancement and tensile stiffness reduction) and the optical performance. We also note that, as

introduced in Section S9 and Figure S24, we designed films featuring a flat reflective layer on top of a corrugated backbone. Such designs may mitigate scattering.

4. *In realistic light-sail operation, the incident laser beam may not always be perfectly normal to the surface, and the sail may experience angular variations during acceleration. It would therefore be helpful if the authors could briefly discuss the expected angular dependence of reflectivity and whether the corrugated geometry might introduce additional angle-dependent optical effect.*

   - We thank Reviewer #4 for pointing this out. It is true that real sail designs will likely have curved shapes [19], which will impact their actual overall reflectivity. For simulations of the impact of the incident light angle on the fabricated and optimized films' reflectivity and absorptivity, please see our response to Reviewer #2 Response 9 on page 7 of this letter, where we introduce Figures S27 and S28 in the Supplementary Information. These figures contain transfer-matrix method simulations of the films' reflectivity and absorptivity as a function of angle and wavelength. For the impact of corrugation on the angle-dependence of reflectivity and absorptivity, we suggest that detailed simulations will be required that we consider to be beyond the scope of this work. However, it may be possible for future designs to account for this; see Section S18 in the Supplemental Information.
   - SI: Note that, especially in the case of curved sails [19], some light will encounter the film at off-normal angles. To estimate the change in optical properties that this could entail, we simulated the reflectivity and absorptivity of the fabricated prototype sail film using our measured indices of refraction (see Section S8). The results, shown in Figure S27, suggest that the angular dependence of the sail's reflectivity and absorptivity is only moderate; within the laser band ($\lambda = \langle 1.2, 1.4697 \rangle$ µm), the reflectivity and absorptivity change by up to 9% and 2.5%, respectively, within a 0-to-50° angular range.
   - SI: In addition, for reference and completeness, we calculated the angular dependence of the reflectivity and absorptivity of our proposed optimized sail film using the optical data listed above [3, 29]; the results are provided in Figure S29.

5. *The scalability of the fabrication process could be discussed in more detail. The current prototypes appear to be on the millimeter scale, and it would be valuable for readers if the authors could comment more explicitly on how the proposed fabrication approach could be extended to significantly larger areas and which steps might represent the main challenges/bottlenecks in scaling the process (whether materials deposition or releasing steps). And if multiple few-inch-sized lightsails are required, how would they be assembled or connected into a larger practical lightsail?*

   - We thank Reviewer #4 for this important comment. We have included in Section S21 of the Supplementary Information a brief discussion about scaling our films to larger areas. In response to their valuable questions about bottlenecks to scaling and assembly of pieces into a larger whole, we added a few additional thoughts to this section, too.
   - SI: A potential challenge to this approach is that photolithography is limited to wafer-scale substrates, implying that many films would need to be assembled to achieve a full light sail. It would be important to ensure that all of the photoresist was removed from the film in this process, which could conceivably be accomplished using O_2 plasma ashing and verified using fluorescence microscopy or related diagnostics.
   - SI: As in the previous option, it would be important to ensure that the entire sacrificial film was eliminated at the end of the process.
   - SI: A potential bottleneck of this approach is that the DRIE process is limited to wafer-scale lateral areas. In addition, it would be important to protect the corrugated film on the front side of the wafers during the backside etching process. Whereas the wafer can be suspended in mid-air in the laser rastering method (so the corrugated film is not scratched), in DRIE the wafer must be in firm thermal contact with the tool chuck to ensure sufficient cooling. One possibility would be to sputter a temporary thin aluminum coating on the corrugated film for protection, which could be wet-etched away after the DRIE step but before the final XeF_2 etch.
   - SI: To connect several films together, the pieces could be stitched to a transparent semi-rigid frame [8, 19], likely made from a ceramic material such as Al_2O_3. Alternatively, pieces could be

tiled and hooked to one another like pieces in a puzzle, potentially eliminating the need for a large frame [32]. A final option could be a form of thermal spot welding to join corners of the films together into a large array [33].

6. *Another aspect that may deserve discussion is the stability of the proposed materials in the space environment. How robust are the Al_2O_3/MoS_2 nanolaminate films against radiation damage, micrometeoroid impacts, or long-term exposure to the space environment? A brief discussion on the durability of these materials under realistic space conditions would strengthen the manuscript.*

   - We appreciate Reviewer #4's questions, as outer space poses a difficult environment for material survival [34–38]. We believe that Al_2O_3 and MoS_2 are well-suited for use in space because they are currently employed there and have been rigorously tested [39–45]. To aid readers, we included a new section to discuss these concerns in the Supplementary Information (Section S20).

   - SI: Interstellar space is a harsh environment, and during its multi-year transit to another star system the sail film will likely encounter radiation, micro-meteoroids, reactive radicals, and other hazards [34–38]. Although in principle the sail would only need to stay intact for a few minutes during its acceleration phase, it would be beneficial for it to survive the entire journey because (1) were it to disintegrate it could alter the sail's trajectory; (2) the sail could be useful for transmitting data back to Earth [25]; and (3) the sail could be used for slowing the probe as it approached a foreign star to provide more time for observation [46–48]. Several articles have discussed this problem in detail, studying the size distribution of dust particles in space [34], the potential of a foil shield to mitigate impacts from dust and gas particles [35], the impact of dust and gas bombardment on light sails [36], the impact of electromagnetic forces on light sails [37], and the potential for heating of light sails due to dust bombardment [38]. While a thorough discussion of the robustness of our class of light sails is beyond the scope of this work, we briefly offer a few thoughts here. First, as mentioned in the main article, the out-of-plane corrugated structure of our sails prevents crack propagation by deflecting cracks at the vertical trench or rib walls [31]; this may be an important factor given the inevitability of impacts from dust and gas. Second, both Al_2O_3 and MoS_2 have been proposed for use in space - the former as a coating to protect components against atomic oxygen [49] or as thermal insulation [43], and the latter as a solid-based lubricant [40, 50] or as an absorber in photovoltaic cells [41]. This suggests their viability as materials for light sails from a survivability standpoint. Third, several studies have examined the durability of these materials in space (or in vacuum on Earth) [39–42] - some for equivalent durations of months or longer [43–45]. Although this is only a fraction of the required transit time to another star system, it indicates that these materials hold promise and warrant further study.

7. *Another open question concerns the beam-riding stability of such sails during laserdriven acceleration. Since the proposed structure introduces a non-flat geometry, it would be interesting to understand whether this architecture could influence the stability of the sail within the laser beam, either positively or negatively.*

   - We thank Reviewer #4 for raising these questions. Please refer to our comments to Reviewer #2 Response 5 on page 5 of this letter, where we provide information about beam-riding stability and introduce Section S19 in the Supplementary Information.

8. *I have briefly checked the provided GitHub repository. The repository appears to include relevant datasets and scripts used in the study, along with a README file that provides basic documentation. The structure of the repository and the accompanying files seem reasonably organized and should allow interested readers to understand the computational and data analysis procedures used in the manuscript. While I did not attempt to fully run the code, the provided materials appear sufficient in principle to support reproducibility of the reported results.*

   - We thank Reviewer #4 for examining our GitHub repository. We have uploaded new versions of files that were altered as a result of this revision. For convenience, a link to the code is `https://github.com/souperman1985/starshot`.

Thank you again for considering this manuscript for *Nature Communications*. Please do not hesitate to contact me (dmj@seas.upenn.edu) with any questions or concerns you may have. We look forward to hearing from you soon.

Sincerely,

Deep Jariwala

References

[1] Jin Chang, Wenye Ji, Xiong Yao, Arnold J. van Run, and Simon Gröblacher. Broadband, high-reflectivity dielectric mirrors at wafer scale: Combining photonic crystal and metasurface architectures for advanced lightsails. *Nano Letters*, 24(22):6689–6695, 06 2024.

[2] Daniel Franta, David Nečas, Ivan Ohlídal, and Angelo Giglia. Optical characterization of SiO_2 thin films using universal dispersion model over wide spectral range. In Christophe Gorecki, Anand Krishna Asundi, and Wolfgang Osten, editors, *Optical Micro- and Nanometrology VI*, volume 9890, page 989014, Bellingham, WA, USA, 2016. SPIE.

[3] Jan Kischkat, Sven Peters, Bernd Gruska, Mykhaylo Semtsiv, Mikaela Chashnikova, Matthias Klinkmüller, Oliana Fedosenko, Stephan Machulik, Anna Aleksandrova, Gregorii Monastyrskyi, Yuri Flores, and W. Ted Masselink. Mid-infrared optical properties of thin films of aluminum oxide, titanium dioxide, silicon dioxide, aluminum nitride, and silicon nitride. *Appl. Opt.*, 51(28):6789–6798, 2012.

[4] John Brewer, Matthew F. Campbell, Pawan Kumar, Sachin Kulkarni, Deep Jariwala, Igor Bargatin, and Aaswath P. Raman. Multiscale photonic emissivity engineering for relativistic lightsail thermal regulation. *Nano Letters*, 22(2):594–601, 2022.

[5] E. Shkondin, O. Takayama, M. E. Aryaee Panah, P. Liu, P. V. Larsen, M. D. Mar, F. Jensen, and A. V. Lavrinenko. Large-scale high aspect ratio Al-doped ZnO nanopillars arrays as anisotropic metamaterials. *Opt. Mater. Express*, 7(5):1606–1627, 2017.

[6] Jonathan M. Marr and Francis P. Wilkin. A better presentation of Planck's radiation law. *American Journal of Physics*, 80(5):399–405, 2012.

[7] Binjie Huang, Minrui Zheng, Yunshan Zhao, Jing Wu, and John T. L. Thong. Atomic layer deposition of high-quality Al_2O_3 thin films on MoS_2 with water plasma treatment. *ACS Applied Materials & Interfaces*, 11(38):35438–35443, 09 2019.

[8] Harry A. Atwater, Artur R. Davoyan, Ognjen Ilic, Deep Jariwala, Michelle C. Sherrott, Cora M. Went, William S. Whitney, and Joeson Wong. Materials challenges for the Starshot lightsail. *Nat. Mater.*, 17(10):861–867, 2018.

[9] Kevin L. G. Parkin. The Breakthrough Starshot system model. *Acta Astronaut.*, 152:370–384, 2018.

[10] Kevin L. G. Parkin. Cost-optimal laser-accelerated lightsails. *arXiv*, 2023. URL: `https://arxiv.org/abs/2205.13138`.

[11] Claude Phipps, editor. *Laser Propulsion in Space: Fundamentals, Technology, and Future Missions*. Elsevier, New York, NY, USA, 1st edition, 2024.

[12] A West, M van der Schans, C Xu, M Cooke, and E Wagenaars. Fast, downstream removal of photoresist using reactive oxygen species from the effluent of an atmospheric pressure plasma jet. *Plasma Sources Science and Technology*, 25(2):02LT01, 2016.

[13] Bo Jheng Shih, Zih Yang Chen, Shie Ping Chang, Ting Yu Chen, Po Jung Sung, Nien Chih Lin, Chih Chao Yang, Po Tsang Huang, Huang Chung Cheng, Ming Yang Li, Iuliana P. Radu, and Kuan Neng Chen. Surface characteristics optimization during wafer-level backside silicon removal for SOI wafers in 3D integration. *Applied Surface Science*, 688:162366, 2025.

[14] Lucas Norder, Shunyu Yin, Matthijs H. J. de Jong, Francesco Stallone, Hande Aydogmus, Paolo M. Sberna, Miguel A. Bessa, and Richard A. Norte. Pentagonal photonic crystal mirrors: Scalable lightsails with enhanced acceleration via neural topology optimization. *Nature Communications*, 16(1):2753, 2025.

[15] Ognjen Ilic and Harry A. Atwater. Self-stabilizing photonic levitation and propulsion of nanostructured macroscopic objects. *Nature Photonics*, 13(4):289–295, 2019.

[16] Jadon Y. Lin, C. Martijn de Sterke, Michael S. Wheatland, Alex Y. Song, and Boris T. Kuhlmey. All-optical damping forces enhanced by metasurfaces for stable relativistic lightsail propulsion. *Phys. Rev. Appl.*, 22(6):064028, 2024.

[17] Elena Popova, Messoud Efendiev, and Ildar Gabitov. On the stability of a space vehicle riding on an intense laser beam. *Math. Meth. Appl. Sci.*, 40:1346–1354, 2016.

[18] Zachary Manchester and Abraham Loeb. Stability of a light sail riding on a laser beam. *Astrophys. J. Lett.*, 837:L20, 2017.

[19] Matthew F. Campbell, John Brewer, Deep Jariwala, Aaswath P. Raman, and Igor Bargatin. Relativistic light sails need to billow. *Nano Letters*, 22(1):90–96, 2022.

[20] Prateek R. Srivastava, Ying Ju Lucy Chu, and Grover A. Swartzlander. Stable diffractive beam rider. *Opt. Lett.*, 44(12):3082–3085, 2019.

[21] Prateek R. Srivastava and Grover A. Swartzlander. Optomechanics of a stable diffractive axicon light sail. *Eur. Phys. J. Plus*, 135(7):570, 2020.

[22] Mohammad Mahdi Salary and Hossein Mosallaei. Photonic metasurfaces as relativistic light sails for Doppler-broadened stable beam-riding and radiative cooling. *Laser Photonics Rev.*, 14(8):1900311, 2020.

[23] Karthik Vijay Myilswamy, Aravind Krishnan, and Michelle L. Povinelli. Photonic crystal lightsail with nonlinear reflectivity for increased stability. *Opt. Express*, 28(6):8223–8232, 2020.

[24] Niels Gieseler, Aso Rahimzadegan, and Carsten Rockstuhl. Self-stabilizing curved metasurfaces as a sail for light-propelled spacecrafts. *Opt. Express*, 29(14):21562–21575, 2021.

[25] Mohammadrasoul Taghavi, Mohammad Mahdi Salary, and Hossein Mosallaei. Multifunctional metasails for self-stabilized beam-riding and optical communication. *Nanoscale Adv.*, 4(7):1727–1740, 2022.

[26] Mohammadrasoul Taghavi and Hossein Mosallaei. Increasing the stability margins using multi-pattern metasails and multi-modal laser beams. *Scientific Reports*, 12(1):20034, 2022.

[27] Giovanni Santi, Alain J. Corso, Denis Garoli, Giuseppe Emanuele Lio, Marco Manente, Giulio Favaro, Marco Bazzan, Giampaolo

Piotto, Nicola Andriolli, Lucanos Strambini, Daniele Pavarin, Leonardo Badia, Remo Proietti Zaccaria, Philip Lubin, Roberto Ragazzoni, and Maria G. Pelizzo. Swarm of lightsail nanosatellites for Solar System exploration. *Scientific Reports*, 13(1):19583, 2023.

[28] Ramon Gao, Michael D. Kelzenberg, and Harry A. Atwater. Dynamically stable radiation pressure propulsion of flexible lightsails for interstellar exploration. *Nature Communications*, 15(1):4203, 2024.

[29] Battulga Munkhbat, Piotr Wróbel, Tomasz J. Antosiewicz, and Timur O. Shegai. Optical constants of several multilayer transition metal dichalcogenides measured by spectroscopic ellipsometry in the 300–1700 nm range: High index, anisotropy, and hyperbolicity. *ACS Photonics*, 9(7):2398–2407, 2022.

[30] S. Timoshenko and S. Woinowsky-Krieger. *Theory of plates and shells*. McGraw-Hill Book Company, New York, NY, USA, 1959.

[31] Keivan Davami, Lin Zhao, Eric Lu, John Cortes, Chen Lin, Drew E. Lilley, Prashant K. Purohit, and Igor Bargatin. Ultralight shape-recovering plate mechanical metamaterials. *Nature Communications*, 6(1):10019, 2015.

[32] Jin Ho Kang, Keith L. Gordon, Robert G. Bryant, Olive R. Stohlman, W. Keats Wilkie, Amanda E. Stark, Randall S. Barfield, Benjamin R. Sindle, Miria M. Finckenor, and Paul D. Craven. Durability characterization of mechanical interfaces in solar sail membrane structures. *Advances in Space Research*, 67(9):2643–2654, 2021.

[33] E. H. Penilla, L. F. Devia-Cruz, A. T. Wieg, P. Martinez-Torres, N. Cuando-Espitia, P. Sellappan, Y. Kodera, G. Aguilar, and J. E. Garay. Ultrafast laser welding of ceramics. *Science*, 365(6455):803–808, 2019.

[34] Joseph C. Weingartner and B. T. Draine. Dust grain-size distributions and extinction in the Milky Way, Large Magellanic Cloud, and Small Magellanic Cloud. *The Astrophysical Journal*, 548(1):296–309, 2001.

[35] James T. Early and Richard A. London. Dust grain damage to interstellar vehicles and lightsails. *J. Br. Interplanet. Soc.*, 68:205–210, 2015.

[36] Thiem Hoang, A. Lazarian, Blakesley Burkhart, and Abraham Loeb. The interaction of relativistic spacecrafts with the interstellar medium. *Astrophys. J.*, 837(1):5, 2017.

[37] Thiem Hoang and Abraham Loeb. Electromagnetic forces on a relativistic spacecraft in the interstellar medium. *Astrophys. J.*, 848(1):31, 2017.

[38] Gabriel R. Jaffe, Gregory R. Holdman, Min Seok Jang, Demeng Feng, Mikhail A. Kats, and Victor Watson Brar. Effect of dust and hot spots on the thermal stability of laser sails. *Nano Letters*, 23(15):6852–6858, 2023.

[39] E. H. Lan, C. A. Smith, and J. B. Cross. Atomic oxygen effects on candidate coatings for long-term spacecraft in low Earth orbit. Technical Report 19890003221, NASA, Goddard Space Flight Center, January 1 1988. URL: `https://ntrs.nasa.gov/citations/19890003221`.

[40] Xin Fan, Yanbin Shi, Mingjun Cui, Siming Ren, Haixin Wang, and Jibin Pu. MoS_2/WS_2 nanosheet-based composite films irradiated by atomic oxygen: Implications for lubrication in space. *ACS Applied Nano Materials*, 4(10):10307–10320, 2021.

[41] Timothy Ismael, Muhammad Aamir Abbas, Owen P. Harris, George B. Ingrish, Meghan E. Bush, Joshua M. Sasson, Jeremiah S. McNatt, and Matthew David Escarra. High-specific power flexible photovoltaics from large-area MoS_2 for space applications. *ACS Applied Energy Materials*, 8(1):87–98, 2025.

[42] Jiang Bin-zhi, Xiang Yang, Mo Chen, Peng Zhi-hang, Wen Jin, Cao Jian-ping, and Li Hai-long. Space environment properties of Al_2O_3f/Al_2O_3 composites. *Ceramics International*, 52(3):3470–3480, 2026.

[43] Péter Ádám, Levente Dudás, Ottó Temesi, András Nagy, and Katalin Sinkó. Porous aluminum oxide insulation materials tested in space mission. *CEAS Space Journal*, 15(5):671–680, 2023.

[44] Xiaoming Gao, Ming Hu, Yanlong Fu, Lijun Weng, Weimin Liu, and Jiayi Sun. MoS_2-Au/Au multilayer lubrication film with better resistance to space environment. *Journal of Alloys and Compounds*, 815:152483, 2020.

[45] Jun He, Desheng Wang, Ming Hu, Xiaoming Gao, Jiayi Sun, Zhenhua Li, Hongyu Lv, Longbang Guo, Yanlong Fu, Zhilu Liu, Dong Jiang, Qinqin Wang, Jun Yang, Xu Zhao, and Lijun Weng. Unveiling the degradation mechanisms of MoS_2 lubricating films in authentic low earth orbit *via* China space station exposure experiment. *Advanced Functional Materials*, 36(10):e23156, 2026.

[46] Robert L. Forward. Roundtrip interstellar travel using laser-pushed lightsails. *J. Spacecr. Rockets*, 21(2):187–195, 1984.

[47] René Heller, Michael Hippke, and Pierre Kervella. Optimized trajectories to the nearest stars using lightweight high-velocity photon sails. *The Astronomical Journal*, 154(3):115, 2017.

[48] André Füzfa, Williams Dhelonga-Biarufu, and Olivier Welcomme. Sailing towards the stars close to the speed of light. *Phys. Rev. Research*, 2(4):043186, 2020.

[49] Timothy K. Minton, Bohan Wu, Jianming Zhang, Ned F. Lindholm, Aziz I. Abdulagatov, Jennifer O'Patchen, Steven M. George, and Markus D. Groner. Protecting polymers in space with atomic layer deposition coatings. *ACS Applied Materials & Interfaces*, 2(9):2515–2520, 2010.

[50] Duval A. Johnson, Marcello Gori, Azhar Vellore, Andrew J. Clough, Scott D. Sitzman, Jeffrey R. Lince, and Ashlie Martini. Evaluation of a commercial MoS_2 dry film lubricant for space applications. *Lubricants*, 12(9):307, 2024.

Review Report

The manuscript by M.F. Campbell *et.al.* proposed a three-layer $Al_2O_3/MoS_2/Al_2O_3$ nanolaminate architecture combined with a hexagonally corrugated microstructure to simultaneously address the optical, mechanical, and mass constraints required for relativistic light-sail propulsion. The authors fabricate prototype films using semiconductor-compatible processes and characterize their structural and optical properties, reporting broadband reflectivity exceeding 50% and absorptivity below approximately 4% in the proposed laser band. This work also analyzes the potential propulsion performance and compares the proposed design with previously reported lightsail concepts. Overall, the manuscript is interesting and solid in terms of concept and methodology, and the idea of combining multilayer nanolaminate optics with architected corrugated mechanical structures is promising. I am generally supportive of publication if the authors could clarify several points and further discuss some aspects of the design motivation and optical implications of the system. See detailed questions below:

(1) the manuscript introduces a hexagonally corrugated architecture to enhance bending stiffness and mechanical robustness, but the motivation for choosing this corrugated geometry over a planar multilayer structure could be explained more clearly. In particular, it would be helpful if the authors could elaborate in the earlier part of the manuscript on why a corrugated architecture is preferred instead of a three-layer planar film with increased thickness or other mechanical reinforcement strategies. Quantitative comparisons between planar and corrugated configurations, for example in terms of stiffness-to-mass ratio or wrinkle suppression, would help justify the design choice.

(2) while the mechanical advantages of the corrugated geometry are discussed in detail, the manuscript does not fully address how this out-of-plane structure may influence the optical performance. Because the architecture introduces edges, trenches, and non-planar surfaces, it would be useful for the authors to comment on whether the corrugation could introduce additional scattering or reflection losses, and whether such effects were observable or negligible in the optical measurements presented in the main manuscript.

(3) In realistic light-sail operation, the incident laser beam may not always be perfectly normal to the surface, and the sail may experience angular variations during acceleration. It would therefore be helpful if the authors could briefly discuss the expected angular dependence of reflectivity and whether the corrugated geometry might introduce additional angle-dependent optical effect.

(4) the scalability of the fabrication process could be discussed in more detail. The current prototypes appear to be on the millimeter scale, and it would be valuable for readers if the authors could comment more explicitly on how the proposed fabrication approach could be extended to significantly larger areas and which steps might represent the main challenges/bottlenecks in scaling the process (whether materials deposition or releasing steps). And if multiple few-inch-sized lightsails are required, how would they be assembled or connected into a larger practical lightsail?

(5) Another aspect that may deserve discussion is the stability of the proposed materials in the space environment. How robust are the Al_2O_3/MoS_2 nanolaminate films against radiation damage, micrometeoroid impacts, or long-term exposure to the space environment? A brief discussion on the durability of these materials under realistic space conditions would strengthen the manuscript.

(6) Another open question concerns the beam-riding stability of such sails during laser-driven acceleration. Since the proposed structure introduces a non-flat geometry, it would be interesting to understand whether this architecture could influence the stability of the sail within the laser beam, either positively or negatively.
